# Antagonism Pattern Detection between MicroRNA and Target Expression in Ewing’s Sarcoma

**DOI:** 10.1371/journal.pone.0041770

**Published:** 2012-07-25

**Authors:** Loredana Martignetti, Karine Laud-Duval, Franck Tirode, Gaelle Pierron, Stéphanie Reynaud, Emmanuel Barillot, Olivier Delattre, Andrei Zinovyev

**Affiliations:** 1 Institut Curie, Paris, France; 2 INSERM U900, Paris, France; 3 Mines ParisTech, Fontainebleau, France; 4 INSERM U830, Paris, France; 5 Department of Tumor Biology, Institut Curie Hospital, Paris, France; University of Turin, Italy

## Abstract

MicroRNAs (miRNAs) have emerged as fundamental regulators that silence gene expression at the post-transcriptional and translational levels. The identification of their targets is a major challenge to elucidate the regulated biological processes. The overall effect of miRNA is reflected on target mRNA expression, suggesting the design of new investigative methods based on high-throughput experimental data such as miRNA and transcriptome profiles. We propose a novel statistical measure of non-linear dependence between miRNA and mRNA expression, in order to infer miRNA-target interactions. This approach, which we name antagonism pattern detection, is based on the statistical recognition of a triangular-shaped pattern in miRNA-target expression profiles. This pattern is observed in miRNA-target expression measurements since their simultaneously elevated expression is statistically under-represented in the case of miRNA silencing effect. The proposed method enables miRNA target prediction to strongly rely on cellular context and physiological conditions reflected by expression data. The procedure has been assessed on synthetic datasets and tested on a set of real positive controls. Then it has been applied to analyze expression data from Ewing’s sarcoma patients. The antagonism relationship is evaluated as a good indicator of real miRNA-target biological interaction. The predicted targets are consistently enriched for miRNA binding site motifs in their 3′UTR. Moreover, we reveal sets of predicted targets for each miRNA sharing important biological function. The procedure allows us to infer crucial miRNA regulators and their potential targets in Ewing’s sarcoma disease. It can be considered as a valid statistical approach to discover new insights in the miRNA regulatory mechanisms.

## Introduction

MicroRNAs (miRNAs) are single-stranded RNA molecules of 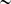
22 nucleotides recently emerged as post-transcriptional regulators of gene expression. By computational predictions, experimental approaches or combined strategies, nearly one third of human protein-coding genes are estimated to be regulated by miRNAs [1], [2]. Given the wide scope of their targeting, miRNAs might be considered as another layer of the regulatory circuitry existing in the cell. Nevertheless, compared with the regulation of transcription, the study of the regulatory mechanisms by miRNAs is only at its beginning.

Multicellular eukaryotes use miRNAs to regulate many biological processes. In animals, examples of documented miRNA functions include regulation of signaling pathways, apoptosis, metabolism, cardiogenesis and brain development [3], [4]. In addition, recent studies have shown that miRNAs may provide new insights in cancer research. Misregulation of miRNA expression has been linked to many types of cancer [5], [6]. Furthermore, miRNA expression profiles have been shown to successfully classify poorly differentiated tumors, with a higher potential of cancer diagnosis compared to mRNA profiles [7].

The molecular mechanisms of miRNA action remain intensely debated. There are evidences for multiple modes of miRNA-mediated regulation, including translational inhibition, increased mRNA de-adenylation and degradation, and/or mRNA sequestration [8]. Systematic analysis of mRNA and miRNA expression demonstrates that simultaneous profiling of miRNA and mRNA expression can be used on a large scale to identify functional miRNA-target relationships [9]. Many miRNAs cause degradation of their targets and a large number of genes are regulated in this way. Recent works addressed this problem with a high-throughput proteomic approach to quantify level of thousands of proteins in the presence or absence of a certain miRNA [10]–[12]. Results show that upon introduction (or knockdown) of a miRNA, the synthesis of hundreds of proteins is affected, but effects are mild, with few proteins decreasing by more than 50%. This implies that miRNAs fine-tune gene expression, rather than inducing dramatic changes. Furthermore, the analysis of mRNA levels allow to distinguish between two main modes of miRNA action: mRNA degradation and translational inhibition.

Since the discovery of miRNAs, the identification of genuine targets is a key issue to decipher their role in different biological processes. To date, the experimentally validated miRNA interactions are little more than 3500 in all species [13], [14]. In silico target prediction represents a fundamental step in inferring new miRNA-target interactions. Sequence based prediction algorithms are mainly based on empirically determined features of how known miRNAs bind in vivo [15]–[18]. The restricted biological knowledge makes the design and validation of novel investigative methods very difficult. Different algorithms provide different predictions, and the degree of overlap between retrieved lists of predicted targets is often poor or null [19]–[21]. Predictions by purely sequence based methods suffer from lack of information regarding the cellular context of gene regulation. A major source of information to infer the actual regulatory activity of miRNAs derives from high-throughput experimental data such as transcriptome profiles. The basic idea is that regulatory activity by miRNAs could be reflected by the expression changes of their target transcripts. Several works reported genome-wide measure of correlation between miRNA and mRNA expression to identify target genes [9], [22]–[24]. To improve the detection of reliable targets, miRNA and mRNA expression data can be integrated to sequence based predictions by a Bayesian inference method [25], by systematic correlation analysis [26]–[28] or by adopting multiple statistical measures of profile relatedness [29].

**Figure 1:**
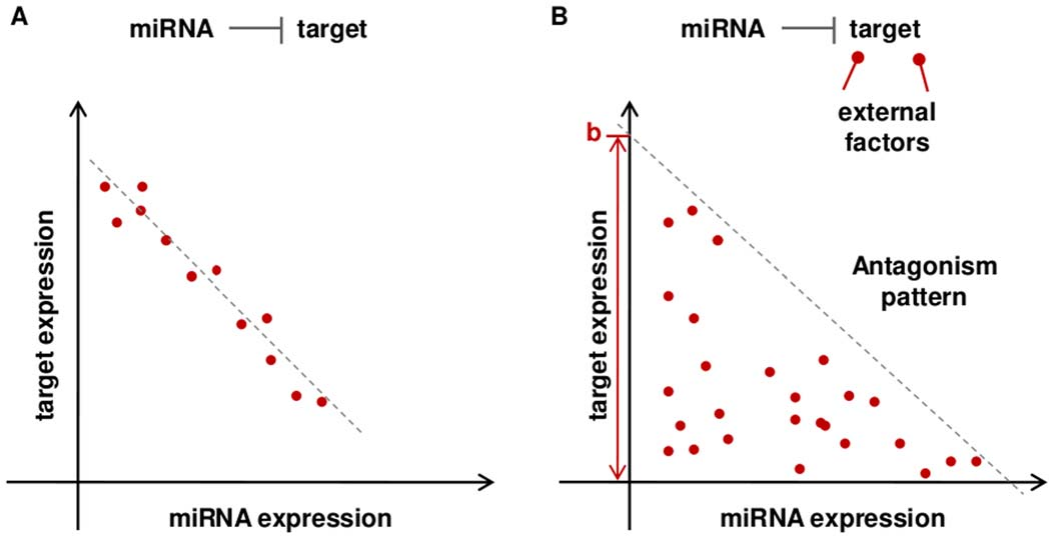
Two different relationships can be distinguished between the expression of a given miRNA and a target. In the first case, miRNA regulation is the main visible effect on target expression and the observed pattern is linear (1a). In the second situation, the effect of miRNA is modulated by other additional factors and the resulting pattern is non linear (1b).

We propose here a novel measure of dependence between miRNA and mRNA expression to infer miRNA-target interactions. We assume that miRNAs and target transcripts can create a non-linear expression pattern due to the effect of cancer specific genomic alterations, additional regulatory factors and external noise. Two different relationships can be distinguished between a given miRNA and their targets, as depicted in Fig.1. In the first case, miRNA regulation is the main visible effect on target expression and the observed pattern is linear (Fig.1a). In the second situation, the effect of miRNA is modulated by other additional factors and the resulting pattern is non linear (Fig.1b). Pair-wise measures of miRNA and target mRNA expression display different possible conditions, due to sample variation and experimental fluctuations. The measure can reflect: (1) elevated miRNA expression level associated to low target mRNA level; (2) low miRNA expression associated to high target mRNA level; (3) low expression of both miRNA and target expression. Indeed, the global miRNA-target expression profile creates a recognizable pattern, with a statistical under-represented event corresponding to the presence of both miRNA and target mRNA elevated expression. This event is statistically less expected compared to the previously described ones in the case of miRNA silencing effect on the target mRNA. We call this kind of triangular-shaped pattern an event of antagonism between miRNA and target mRNA.

**Figure 2:**
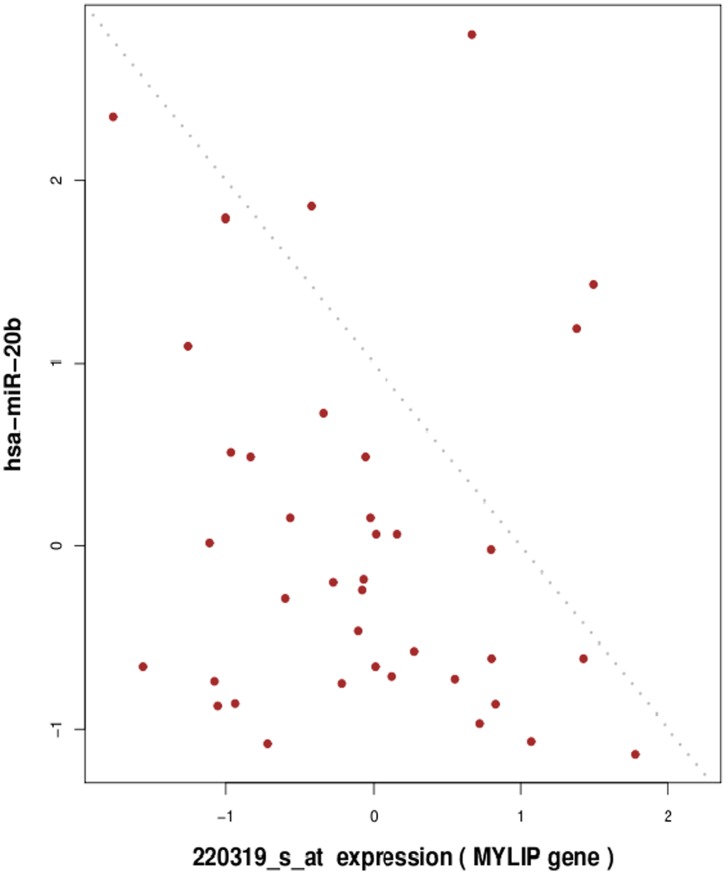
A representative example of the antagonism pattern relationship observed in real data between expression of *hsa-miR-20b* and its target gene *MYLIP* (Pearson *r* = −0.303, *PV* = 0.06, Antagonim *PV* = 0.048).

The antagonsim pattern detection described herein is based on large-scale analysis of paired miRNA and mRNA expression profiles. The procedure requires a set of coupled miRNA and mRNA expression measures from the same samples. The expression profile of each miRNA-mRNA pair across samples is scored for the presence of antagonism pattern. A representative example of antagonism pattern observed in Ewing’s sarcoma data between *hsa-miR-20b* and its target gene *MYLIP* is illustrated in Fig.2. The example, based on an experimentally validated interaction [30], also illustrates how the antagonism is not properly detectable by correlation analysis with 5% significance level. The antagonism pattern highlights an alternative relationship between miRNA and target mRNA expression with respect to linear regression. We consider this statistical signal as a good indicator of real miRNA-target biological interaction.

The measure of antagonism is a particularly suitable method to elucidate the role of miRNA regulation in tumoral diseases. In this context, physiological processes of gene regulation are widely altered. Tumor samples can present a high level of genomic heterogeneity, with cancer specific changes in miRNA expression and dysfunction of miRNA located in regions of chromosomal instability [31], [32]. To reveal how frequently the antagonism pattern is observed in cancer related validated examples, we applied our procedure to publicly available microRNA and gene expression datasets corresponding to different tumor diseases and we compared our results with a catalogue of experimentally validated targets.

Later, we applied our method to elucidate the regulatory role of miRNAs in Ewing’s sarcoma. This malignant pediatric tumor is characterized by specific fusions between *EWS* and *ETS* family genes [33], [34]. In 85% of cases, *EWS* gene is fused to *FLI1* [35]. This in-frame translocation creates the *EWS-FLI1* chimeric protein described as an aberrant transcription factor that dysregulates specific target genes involved in tumor development [36]. Since the expression of the *EWS-FLI1* gene alone can change cell phenotype from normal to tumorigenic in fully reversible and controlled manner, this is an excellent system for understanding the complex picture of deregulations happening in cancer cells. The availability of high-throughput data for Ewing patients, including transcriptome and miRNAs expression data, allows us to infer new regulatory interactions involved in the *EWS-FLI1* network and to clarify the impact of miRNA activity.

## Results

### Results Obtained on Simulated Datasets

A series of experiments was performed on simulated datasets to test the antagonism pattern detection procedure. We generated simulated expression-like datasets exhibiting a triangular-shaped pattern. The aim is to simulate a set of expression profiles 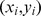, 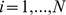, where each profile 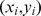 is a bidimensional vector with 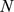 components corresponding to the gene and miRNA expression values in the 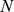 samples. The triangular-shaped structure is specified by choosing simulated values between 0 and 1, satisfying the condition 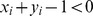. We performed 1000 estimations of antagonism pattern for each simulated dataset, varying the sample number 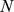 from 20 to 100 and increasing the percentage of noisy points (simulated values not satisfying the condition above) from 0% to 50% of the dataset (details about the simulation procedure in Methods section).

**Figure 3:**
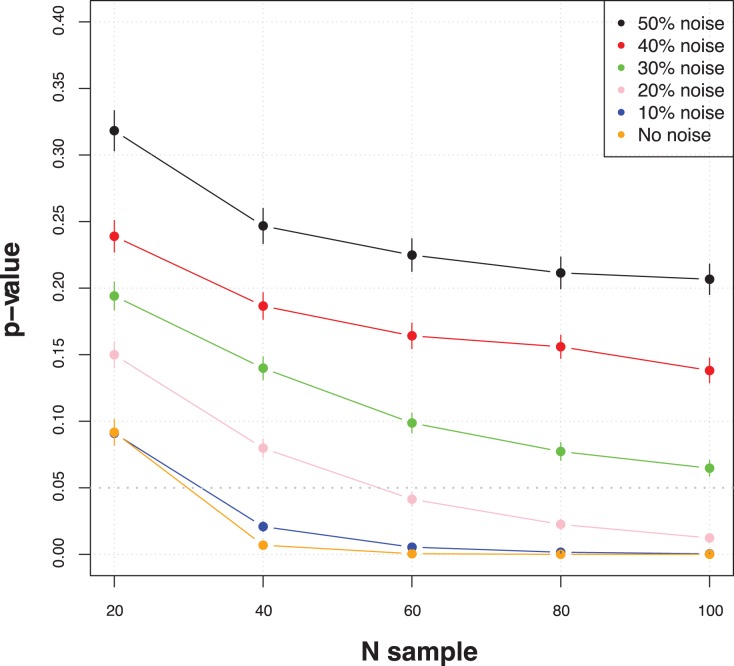
Antagonism pattern p-value variation as function of sample number for different level of noise (results obtained on simulated datasets).

The effect of two main variables was examined to determine how they influence the assigned antagonism pattern p-value (defined in Methods), namely the number of available samples 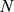 for miRNA and mRNA expression measure and the precentage of noisy data. In Fig.3 the p-value variation was plotted as function of sample number for different noise levels. The described procedure is able to recognize the antagonism pattern with decreasing p-value according to the greater number of available measures and to the decreasing noise level. The number of samples is critical to detect the pattern. With less than 40 samples, the antagonism relationship can not be determined with significant p-value. With a sample number higher than 40, the antagonism pattern can be detected with p-value 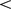 0.05, tolerating up to 10% of noisy data.

### Test on Experimentally Validated miRNA Targets

Appropriate evaluation of our proposed measure requires a set of real positive controls (known and experimentally validated targets). However, a limited number of bona fide miRNA targets have been experimentally validated so far and they have been verified in various organisms and physiological conditions. We collected a catalogue of experimentally validated miRNA-target interactions from two main manually curated resources, namely miRecords and miRTarBase [13], [37]. Merging miRNA-target pairs from these two databases we extracted a list of 1400 interactions verified in human, 265 of them associated to cancer phenotype. We applied the antagonism pattern detection to multiple available datasets of miRNA and gene expression profiles from matched tumor samples and stem cell samples [38]–[41] (Table S1) in order to investigate whether this pattern is observed in a certain number of experimentally validated cancer related examples. We also checked for linear anti-correlation pattern by systematic correlation analysis applied to the same datasets.

**Figure 4:**
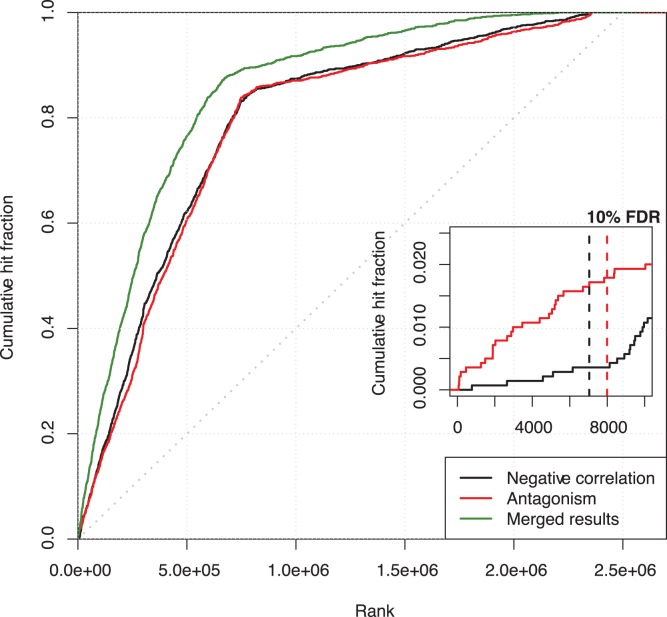
The cumulative frequence of predicted true interactions (hits) plotted as a function of the prediction rank obtained by antagonism pattern and by linear anti-correlation. Merged results show the benefit obtained by the combination of the two methods, obtained by taking the best rank between the two methods for each predicted miRNA-target pair.

To compare results of the two methods, a ranking based evaluation was applied. We plotted the cumulative frequence of true predicted targets (hits) as a function of the prediction rank for the two methods (Fig.4). Results from different datasets have been combined by taking the best rank for each predicted miRNA-target pair. In the resulting plot, antagonism pattern outperforms linear correlation at 10% FDR detecting roughly 4-times more hits compared to linear correlation. Interestingly, a significant benefit is observable for the combination of the two methods (merged results), made by taking the best rank between the two methods for each predicted miRNA-target pair. This shows that the two patterns are likely to identify quite distinct sets of targets. Detailed results obtained for experimentally validated targets are reported in Table S2.

### Antagonism Pattern Detection in Ewing’s Sarcoma

This study includes miRNA and mRNA expression data from 40 samples of Ewing’s sarcoma patients. We collected expression levels of 267 miRNAs and 15651 mRNAs in 40 tumor samples using Illumina human-6 V2 BeadChip and Affymetrix GeneChip HG-U133A/HG-U133Plus2 oligonucleotide microarrays respectively. Normalization and pre-filtering procedures have been applied as described in Methods.

We performed pair-wise antagonism pattern detection to evaluate potential regulatory interaction between each miRNA and each transcript. To determine whether the observed antagonism pattern is significant, the p-value was obtained by using permutation method described in Methods section. In simulated experiments with 40 samples, p-values range between 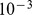 and 0.25 depending on the noise level. In the analysis of Ewing’s sarcoma data, we evaluated the False Discovery Rate (FDR) by using Benjamini-Hochberg method [42], setting a FDR threshold of 2% (see Fig.S1). Once the antagonism pattern p-value between the miRNA 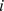 and the transcript 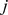 was lower then the fixed threshold (p-value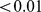), the regulatory relationship between 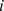 and 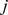 has been inferred.

Significant antagonism pattern has been detected for 7994 miRNA-mRNA pairs, creating a network linking 264 miRNA with 3747 different genes. Almost every miRNA in the initial dataset presents at least one link in the network. Hence, the combinatorial interactions among miRNAs and their targets are probably necessary to specify more precisely the co-regulating nodes and the set of affected targets of each miRNA. We characterized the reconstructed network by the connectivity distribution of miRNAs and genes, defining a list of miRNA hubs and a list of target hubs (details in Methods). According to the outlined distributions, the list of miRNA hubs and target hubs have been compiled and reported in Tables S3A and S3B respectively.

The observation of significant antagonism pattern in expression data can rise from secondary effects rather than from direct regulatory relationships. As most expression-based inference methods, the antagonism pattern detection cannot distinguish between miRNAs that actually regulate a gene (that is, that have a direct causal effect) and miRNAs that show significant antagonism pattern with a gene due to indirect effects. To address this issue and verify that the antagonism pattern captures information about miRNA-gene reliable interactions, two validation analysis have been carried out. In the first one, we verified that miRNA targets identified by antagonism pattern are enriched for binding sites with extensive 5′ miRNA seed pairing in their 3′UTR. A second independent strategy relates to evaluate significantly enriched GO categories for the target sets associated to each miRNA.

### Seed Enrichment

**Figure 5:**
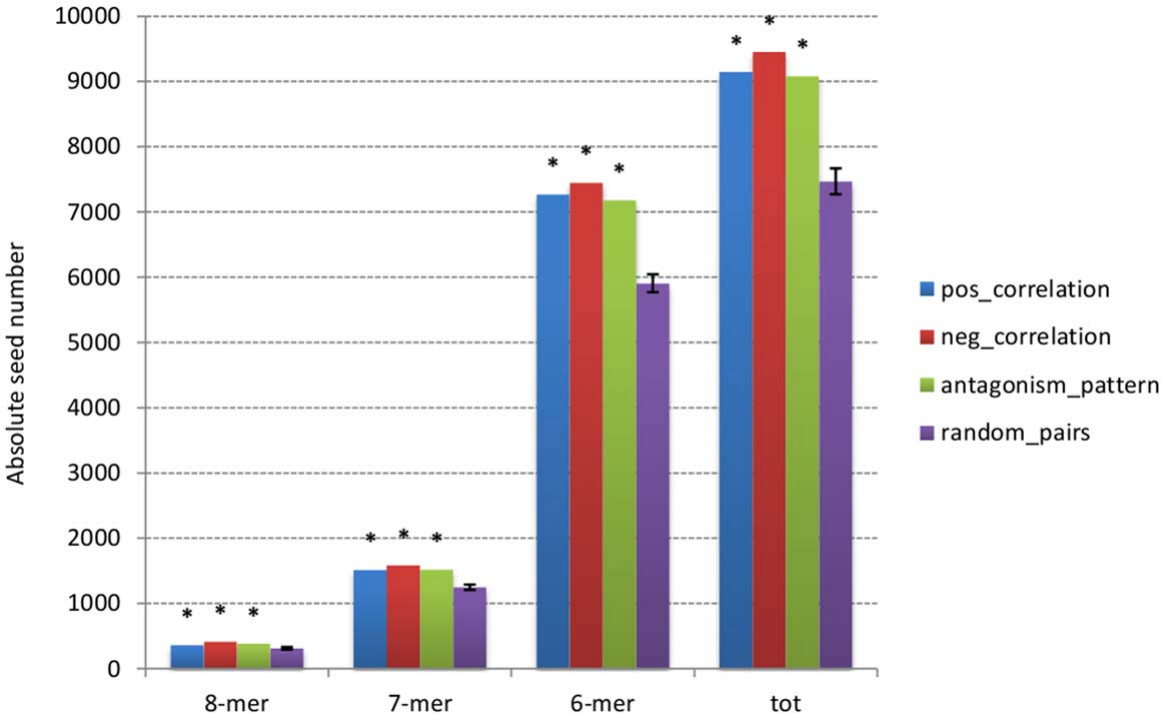
Absolute seed number observed in antagonism pattern predicted targets, in positively and negatively correlated targets and in randomized data sets.

The principle of 5′ seed pairing in miRNA-target binding is well supported by experimental data. Large scale transcriptomics and proteomics studies have recovered gene sets that are enriched in seed matches [10]–[12], [43]. We verified that miRNA targets identified by antagonism pattern are enriched for binding sites with extensive 5′ miRNA seed pairing in their 3′UTR. All possible 8-mer, 7-mer and 6-mer seeds complementary to the first eight nucleotides of the mature miRNA sequence were tested. The seed enrichment is illustrated by the histogram of absolute seed number observed in antagonism pattern predicted targets compared to the randomized miRNA-target pairs (Fig.5). The horizontal axis shows total enrichment for different seed length definition. Randomization of the predicted miRNA-target pairs, performed by shuffling the assignment of miRNAs to their antagonism-based targets, allows the 3′UTR nucleotide composition and length to be preserved (details about the randomization procedure in Methods section). Significant enrichment is obtained for every seed definition (p-values reported in Table S4). This implies that the antagonism pattern captures information about miRNA-genes potentially linked by post-transcriptional regulatory interactions.

### Validation with Gene Ontology

To assess the biological relevance of the identified interactions, we tested for enrichment of miRNA targets involved in a specific process or pathway. The assumption that some miRNAs function as master regulators by downregulating a dense network of genes in the same pathway is supported by several experimental works [44]–. This assumption was first used to investigate changes in mRNA after overexpression of muscle-specific miR-1 and brain-specific miR-124 [43]. Remarkably, transfection of miR-1 into HeLa cells shifted their gene expression profile toward that of muscle cells, whereas transfection of miR-124 shifted the profile toward that of brain cells. Gene ontology and interactome analysis can be used to analyze lists of candidate target genes and some bioinformatics tools have been developed for this purpose [27], [47]. following the reasoning in [9], [48], if the antagonism pattern can successfully identify functional miRNA targets, then the set of predicted targets for each miRNA should have more consistent Gene Ontology (GO) annotations. To verify this, we downloaded annotations from the GO Database [49]. For each miRNA, we created a target set based on antagonism pattern. We scored the GO enrichment within the target sets using hypergeometric test for all GO categories and reported statistically significant results in Table S5. To exclude the hypothesis that our over-represented GO terms can derive from spurious results, we repeated the analysis using random target sets obtained by shuffling miRNA-target relationships. To randomize the original newtork, we randomly permuted target node labels, keeping the miRNA node connectivity unchanged. P-value histogram reported in Fig.S2 shows that no GO term is enriched in random target sets according to the p-value threshold set in the real case.

In a subsequent step, we reconstructed the target sets associated to each miRNA taking into account both antagonism expression pattern and miRNA seed occurrence. Combining these two independent criteria, based respectively on expression profile analysis and DNA sequence information, we expect to increase the reliability of predicted direct targets and to reduce false positive predictions. We scored GO enrichment within the newly defined target sets and report statistically significant results in Table S5. The fact that a large part of GO terms were confirmed by this combined analysis is an important argument in favor of their potential biological relevance. The predicted new regulatory interactions represent reliable miRNA targets for experimental validation. A comprehensive table with results of antagonism detection and seed enrichment for all genes and miRNAs included in the analysis is provided in Table S6.

### Comparison of Antagonism Pattern Detection to Correlation Analysis

Multiple studies report systematic correlation between expression level of miRNAs and their target genes. Correlation of expression profiles is helpfully used to assist identification of miRNA-mRNA regulatory relationships [9], [22]–[24]. Antagonism pattern is expected to point out a different relationship between miRNA and target mRNA expression compared to linear regression. To evaluate the peculiarity of antagonism detection method, the reconstructed network obtained for Ewing’s sarcoma expression data has been compared to the correlation-based network inferred from the same dataset.

Pair-wise correlation coefficients have been computed for the same dataset of 267 miRNAs and 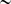
15000 transcripts previously analyzed by antagonism pattern procedure. The procedure for calculating the correlation is described in Methods.

Since antagonism pattern is expected to particularly highlight miRNA degradation effect on the target mRNA, we compared the reconstructed network based on antagonism pattern to correlation-based network, only considering the negatively correlated component. In order to obtain two networks of comparable size, we increased correlation p-value threshold up to 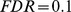. The result included 7040 negatively correlated miRNA-mRNA pairs, with 246 miRNA linked to 3335 different genes. The common links with the antagonism-based network are 1245 (18%). Following the same procedure used for antagonism network, we identified miRNA hubs and target hubs in the correlation network. A set of 22 miRNA hubs (61%) are in common between the two networks (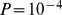, Fisher’s exact test) and it has been reported in Table S7. On the contrary, common target hubs for the two networks are less then 18% (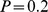, Fisher’s exact test). The two networks are likely to share the same miRNA hub regulators, while identifying largely distinct set of targets.

GO enrichment analysis has been repeated for correlation-based target sets, to evaluate the hypothesis that the two compared methods reveal complementary target sets consistently associated to common biological processes. The results of GO analysis for correlation-based targets are reported in Table S5.

We completed the comparison between antagonism-pattern and correlation-based miRNA-mRNA association, analyzing the seed enrichment for miRNA targets identified by correlation. The histogram in Fig.5 shows the number of seeds for targets predicted by the two different methods and for a randomly created target set. A comparable seed enrichment is obtained for targets predicted by the two methods. Each group significantly differs from the random group, while there is no significant difference in the enrichment obtained by the two compared methods. Interestingly, seed enrichment for positively correlated targets is equally significant and comparable with negatively correlated targets and antagonism-based targets.

## Discussion

In this study we propose a novel statistical measure to integrate large-scale expression data of miRNAs and protein-coding mRNAs, in order to infer miRNA-target interactions. The basic assumption is that miRNA-target expression profile can create a recognizable non-linear pattern corresponding to the statistically under-represented event of simultaneous miRNA and target mRNA elevated expression. This triangular-shaped pattern, that we defined as antagonism pattern, points out an alternative and complementary relationship between miRNA and target mRNA expression with respect to linear regression. The measure of antagonism can be considered a particularly suitable method to elucidate the role of miRNA regulation in the context of tumor diseases, where samples can present a high level of genomic heterogeneity and cancer specific changes in miRNA expression. Here we demonstrated the effectiveness of this statistical measure as a good indicator of real miRNA-target biological interaction. A dedicated computational procedure for antagonism pattern detection has been developed and tested using realistic synthetic datasets. The performance of the provided procedure has been assessed, taking into account the variable sample size as well as noise level in expression measures. Once the conditions to successfully evaluate the antagonism pattern have been determined, we tested the performance of the proposed measure on a catalogue of experimentally validated miRNA-target interactions and we compared it to the linear anti-correlation measure. The percentage of real interactions detected by the antagonism pattern is slightly higher than linear anti-correlation, supporting the effectiveness of this novel measure. Furthermore, we observed that the two patterns are likely to identify quite distinct sets of targets. MiRNA-target networks can be viewed as composed of interactions reflecting two alternative patterns in their expression profiles, depending on the influence of additional factors. An interesting work direction can be to give a mathematical description of these two observed patterns showing that the linear relationship between miRNA-target expression can change towards a triangle-shaped function depending on the strength of additional influence factors. Another direction to explore is how to combine the antagonism pattern detection with the linear correlation. An option would be to extend the antagonism pattern detection procedure including the measure of the opposite “agonist” pattern with a new parameter. The distance between the two parameters (antagonist versus “agonist” pattern) could provide a measure of the linear dependence.

We used the antagonism pattern detection to analyze miRNA and mRNA expression profiles from Ewing’s sarcoma patient samples. Through the antagonism pattern detection, the global miRNA-gene regulatory network has been inferred. Connectivity properties of this reconstructed network allow us to specify the co-regulating nodes and the set of affected targets of each miRNA. To verify that the antagonism pattern captures information about miRNA-gene reliable interactions, two validation analysis have been carried out. In the first one, we verified that miRNA targets identified by antagonism pattern are enriched for binding sites with extensive 5′ miRNA seed pairing in their 3′UTR. Results show highly significant seed enrichment compared to the randomly permuted miRNA-target dataset. This implies that the antagonism-based predicted pairs are consistently enriched for potential post-transcriptional regulatory motifs. A second independent validation has been performed by analyzing significantly enriched GO categories for the target sets associated to each miRNA. Results allow us to determine important biological functions potentially mediated by miRNA regulation in Ewing’s sarcoma.

We observed over-representation of GO terms describing several fundamental processes involving miRNA regulation. The highest enrichment was obtained for the target sets associated to *hsa-miR-1* and *hsa-miR-206*. GO enriched terms for these target sets are related to sarcomere, myofibril and contractil fiber part. Consistently, *hsa-miR-1* and *hsa-miR-206*, together with *hsa-miR-133* (also included in our GO analysis results) are known to be muscle specific expressed, to regulate sarcomere organization and to contribute to rhabdomyosarcoma development [50]–[52]. Since the Ewing’s sarcoma samples are surgically extracted from patient bones, the observed results could suggest contamination of patient derived samples by tumor neighbouring tissues and non-tumoral cells. The Lim et al study [43] showed that *hsa-miR-1* preferentially downregulate nonmuscle genes in HeLa cells. The observation of muscle-related gene enrichment in *hsa-miR-1* and *hsa-miR-133* targets can be explained by a second mode of target regulation in which some muscle genes primarily regulated at the transcriptional level may be tuned by functional miRNA target sites [53]. In agreement with previous studies showing that *hsa-miR-1* and *hsa-miR-133* actively shape gene expression patterns in muscle tissue regulating sarcomeric actin organization [52], our GO analysis pointed out a number of actin-related and actin-binding proteins among *hsa-miR-1* and *hsa-miR-133* targets.

Besides muscle specific terms, GO annotations strictly related to basal mechanisms of cancer were identified. Three miRNAs (*hsa-miR-29b*, *hsa-miR-127* and *hsa-miR-369-3p*) were linked to targets involved in cell cycle and mitotic spindle organization. The involvement of *miRNA-29b* in different human cancers is reported in multiple studies [54]–[56]. The described regulatory circuitry *NF-kappaB*-*YY1*-*miR-29*, whose disruption contributes to rhabdomyosarcoma, suggests that *miR-29* acts as a tumor suppressor. The *miR-127* also exhibits tumor suppressor activity by targeting *BCL6* proto-oncogene and it is silenced in various cancer cell lines [57]. According to our knowledge, *hsa-miR-369* involvement in cancer is not yet documented, while it is known to play a peculiar role in translational efficiency on cell cycle exit under growth arrest condition [58]. It is interesting to notice that *hsa-miR-127* and *hsa-miR-369* are expressed as part of the same miRNA cluster in the human *Dlk1/Gtl2* domain at chromosome *14q32*. This domain is expressed in a large non-coding transcriptional unit which is altered in different tumor diseases and in aggressive ovarian cancer [59], [60]. Two other miRNAs included in our GO results belong to the same genomic domain (*hsa-miR-379* and *hsa-miR-410*). Their targets are associated to extracellular-matrix (ECM) and collagen. Matrix remodeling and collagen protein accumulation are important mechanisms during bone tissue formation from mesenchymal cells. Numerous gene products involved in the cytoskeleton and the ECM contribute to describe mesenchymal stem cell features of Ewing tumors [61]. Recent studies describe miRNA involvement in osteoblast phenotype regulation and they point out miR-29b role in osteoblasts differentiation [46], [62]. A large group of miRNAs in our results were associated to ECM related functions, with highest enrichment for the target sets associated to *hsa-miR-148b*, *hsa-miR-30d*, *hsa-miR-324-3p*, *hsa-miR-379*, *hsa-miR-410*. The role of *miR-30* in matrix remodeling is already confirmed by experimental evidences and one predicted target gene (*CTGF*) has been validated as direct target of this miRNA [63]. Finally a group of miRNAs is involved, according to our GO analysis, in regulatory mechanisms such as RNA-processing, RNA-splicing, protein ubiquitination.

In the final part of this work, we compared antagonism-based results obtained in Ewing’s sarcoma study with results obtained by linear regression analysis. The antagonism-based network obtained from Ewing’s sarcoma data has been compared to the correlation-based network inferred from the same dataset. This comparison points out that the two methods are likely to reconstruct networks with the same miRNA hub regulators but largely distinct target genes. A list of common miRNA hubs, predicted as relevant regulators in Ewing’s sarcoma, has been provided. Results of GO analysis for correlation-based targets are consistent with those obtained from antagonism pattern predictions. As for antagonism-based gene sets, significant association to sarcomere-related terms is obtained for *hsa-miR-1*, *hsa-miR-206* and *hsa-miR-133*. In common with antagonism-based GO analysis, we found strong association of *hsa-miR-30d* target set to ECM related terms. Interestingly, the experimentally validated target *CTGF*, identified by antagonism pattern, is not included in the correlated gene set. Other three miRNA target sets are strongly enriched for ECM and collagen terms: *hsa-miR-152*, *hsa-miR-199b* and *hsa-miR-145*. The latter, together with the co-transcribed *hsa-miR-143*, are known to be deregulated in several tumor types and they both act as tumor suppressor genes in Ewing’s sarcoma as well as in human gastric cancer [64], [65]. The correlation analysis reveal some miRNAs strongly enriched for cell-cycle related terms: *hsa-miR-17*, *hsa-miR-484*, *hsa-miR-93* and *hsa-miR-9*. The *hsa-miR-17* is part of the well studied *mir-17–92* cluster described as human oncogene. The *hsa-miR-9* is known to be involved in human gastric carcinoma as well as in Huntington’s disease [66], [67].

In agreement with analogous large-scale analysis of miRNA-mRNA correlation, we found that positive correlations account for the majority of significantly correlated pairs (67% of links). This effect can be partially explained by miRNA-mRNA genomic co-localization as well as by intronic miRNAs showing correlated expression pattern with their host genes [68]. Another intriguing hypothesis suggests that high positive correlation could be an effect of spatially reciprocal expression domains of miRNAs and their targets in various types of tissues composing patient samples. Recent analysis of large collections of miRNA and gene expression profiles from different types of tissues support the evidence that miRNA and their targets are expressed in a largely non-overlapping manners (spatially or temporally) [69], [70]. This mutually exclusive expression, coordinated by common transcriptional regulators, confer robustness to gene expression program, ensuring tissue identity. Consistently, architectural features of the mammalian miRNA regulatory network reveal that the coordinated transcriptional regulation of a miRNA and its targets is an abundant motif in gene networks [71]–[73].

The antagonism based network has been compared only with the Pearson correlation network. There are methods that use non-parametric correlation or mutual information for assessing non-linear dependences in expression data. The major problem in mutual information analysis of biological data is the reliable estimation of entropy-like quantities from small datasets. Mutual information is difficult to estimate accurately with limited amount of noisy samples. The impact of the entropy estimation on the quality of the inferred transcriptional networks has been recently studied taking a minimum of 50 required samples [74]. Although this type of analysis is more and more used with the availability of large biological datasets, it is not applicable to study non-linear relationships in datasets of limited size.

In summary, the antagonism pattern detection can be successfully used to integrate large-scale expression data of miRNA and protein-coding mRNAs, in order to infer crucial miRNA regulators and miRNA-target interactions. The antagonism pattern can identify alternative relationship between miRNA and target mRNA expression not properly detectable by correlation analysis. Indeed, it can be considered a valid statistical approach to discover new insights in the miRNA regulatory mechanisms.

## Materials and Methods

### Ethics Statement

Ethics approval was provided by the locally appointed ethics committee from the Institut Curie, Paris, France. All participants involved in the present study provided written informed consent.

### miRNA and mRNA Expression Data

Total RNAs issued of 40 Ewing tumors were used for mRNA and miRNA microarray analyses. The mRNA data were collected using the Affymetrix GeneChip HG-U133A and Affymetrix GeneChip HG-U133Plus2. We normalized and combined information across different platforms using the standard functions provided by Bioconductor packages. We used the combineAffyBatch function in the matchprobes library to merge data from HG-U133A and HG-U133Plus2 GeneChips and we applied RMA normalization to the merged dataset. The miRNA expression profiling panel was performed by Integragen (Integragen SA, Evry, France) using Illumina human-6 V1 BeadChip (based on miRbase release 9.1) and average normalization method have been applied according to the manufacturer’s suggested procedure [75]. The average normalization method computes a global scaling factor that is applied to all probes and all arrays. The normalized intensities and detection p-values were exported and further analyzed using Bioconductor packages. MiRNA and gene expression data are available from the NCBI Gene Expression Omnibus (GEO), accession number GSE37372.

### Data Filtering

Before running antagonism pattern detection and correlation analysis, we filtered low abundant miRNAs and transcripts in order to gain more confidence in the results. We also filtered those data with little variation across the 40 samples because the absence of high variation will result in correlations mainly due to noise. Variation across samples has been evaluated by IQR, using the median IQR as cutoff. These criteria left 267 miRNAs and 15651 probe sets for the analysis.

### Algorithm to Detect the Antagonism Pattern between miRNA and Target mRNA

The proposed approach requires as input the genome-wide expression profile of miRNAs and mRNAs from the same set of samples. As final result of our analysis we obtain miRNA-mRNA pairs showing statistically significant antagonism pattern, which we consider as potential miRNA-target interactions.

The antagonism detection procedure can be described as follows. Once converted miRNA and mRNA expression values to 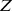-scores using mean and standard deviation across samples to make expression measures comparable, we count the number of points in the region below the diagonal identified by the estimated intercept parameter 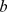 as in equation 1:
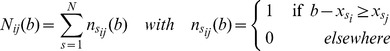
(1)where 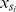 and 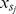 are 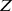-scores transformed expression intensities of miRNA 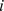 and mRNA 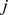 in the sample 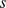, 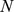 is the total number of samples and 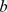 the estimated intercept parameter value.

We define the antagonism coefficient 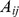 between miRNA 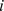 and mRNA 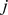 as such 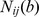 with intercept parameter value 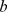 maximazing the Kolmogorov-Smirnov statistic:
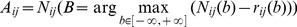
(2)where 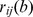 is the reference antagonism coefficient obtained by randomly shuffling sample values (null distribution). This null distribution has been generated by randomly shuffling one hundred times the components of miRNA 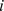 and mRNA 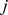 expression vectors. In the null model defined in this way, the data has the same distribution of values as the original one but the association between the miRNA and the mRNA expression is random. This randomization is applied for every possible value of the intercept parameter 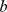 in an exhaustive search space to find the best value 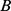. Setting of the parameter 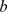 is specific for each 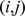.

The statistical significance of the antagonism coefficient 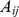 is assessed by permutation-based p-value (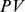). The antagonism coefficient of randomly shuffled sample values 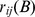 has been computed for 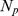 = 1000 permutation rounds and the empirical distribution of 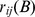 is used to approximate the null distribution. The antagonism pattern 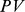 has been obtained as the following ratio:
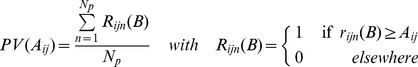
(3)


Once 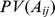 is below the fixed threshold, the antagonism relationship between miRNA 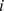 and mRNA 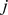 is inferred. PV threshold is set according to the FDR estimated by Benjamini-Hochberg method [42].

The JAVA code implementing the algorithm is available on request from the authors.

### Simulation of Expression Data

To generate expression-like datasets exhibiting a triangular-shaped pattern, we created bidimensional vectors 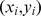, 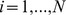, with 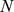 corresponding to the number of samples, 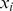, 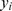 uniformly drawn from the triangular region where 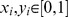 and 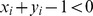. One thousand simulated datasets have been generated for each sample size 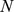 from 20 to 100. We tested six noise levels ranging from not-noisy to very noisy data. Noise has been introduced as an increasing percentage of points not satisfying the condition above. These noisy points are uniformly drawn from the triangular region where 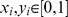 and 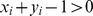. For each noise levels, we simulated 1000 datasets and detected the antagonism pattern using procedure identical to the actual data analysis.

### Randomization Procedure for Seed Enrichment Analysis

To generate randomized miRNA-target pairs, the assignment of miRNAs to their antagonism-based targets has been randomly permuted. This procedure allows us to obtain randomized miRNA-target pairs with the same 3′UTR nucleotide composition and length as the original pairs. MiRNA-target assignments has been permuted by a shuffling ubiased algorithm implemented in PERL, so that every permutation is equally likely. Randomized miRNA-target pairs have been generated 1000 times and 8-mer, 7-mer and 6-mer seed counts have been computed at each time. We tested the significance of the 8-mer, 7-mer, and 6-mer counts obtained for real data using as null model a normal distribution with mean and variance obtained in randomized count data.

### Correlation Analysis

The expression profiles of 267 miRNAs have been correlated with changes in mRNA expression for all 15651 transcripts according to Pearson coefficient. When dealing with a limited group of 40 patient derived samples, the presence of outliers could be a crucial problem affecting the correlation analysis. We applied a leave-one-out strategy to identify frequent non-consistent measures in transcripts and miRNA expression data. Outliers are recognized as measures that dramatically change the correlation estimation. The correlation coefficient between the miRNA 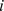 and the transcript 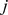 is evaluated 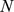 times, eliminating at each step one sample. Assuming normally distributed coefficient values, we consider as outliers that samples that, if eliminated, give a coefficient observation that deviates by twice the standard deviation or more from the mean. This procedure allows us to correct 90% of the correlation coefficients, obtaining more robust correlation estimations. We evaluated the 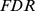 by using the Benjamini-Hochberg method [42]. Fixing a 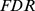 level of 0.1, we obtained a correlation network including 3335 different genes and 264 miRNAs (only negative correlation has been considered in this network).

### Hub Analysis

The reconstructed antagonism-based network has been characterized by the connectivity distribution of genes and miRNAs, in order to identify miRNA hubs (miRNAs linked to a high number of genes in the networks) and target hubs (genes linked to a high number of miRNAs). Using a thresholding and unweighted procedure to define the connectivity, we sum the number of statistically significant links assigned to each node 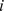 in the network (according to previously fixed threshold):
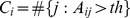
(4)where 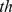 is the fixed 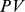 threshold. Then we reconstructed the connectivity distributions of miRNA nodes and target nodes separately. We compared the real distributions with those obtained by the antagonism-based network computed by randomly shuffling sample values (randomized data network). MiRNA hubs were defined as miRNA nodes with 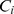 greater than the 75th percentile of the maximal value in the randomized data network distribution. Target hubs have been extracted in analogous way. According to this procedure, miRNA hubs were determined as miRNAs targeting more than 614 genes (providing a list of 82 miRNA hubs) and target hubs were determined as genes which are targeted by more than 16 miRNAs (there were 3631 such genes).

## Supporting Information

Figure S1 False discovery rate (FDR) as a function of the Antagonism p-value obtained in Ewing’s sarcoma study.
(PDF)

Figure S2 GO enrichment p-value distribution for randomized target sets obtained by shuffling real miRNA-target relationships. The dotted vertical line is drawn at the p-value threshold used for GO analysis in real data.
(PDF)

Table S1 Public datasets used for the test on experimentally validated miRNA targets.
(PDF)

Table S2 Detailed results obtained for the test on experimentally validated targets.
(PDF)

Table S3 (A) List of miRNA hubs in the antagonism based network of Ewing’s sarcoma. (B) List of target hubs in the antagonism based network of Ewing’s sarcoma.
(PDF)

Table S4 P-values obtained for the seed enrichment analysis of the antagonism based network.
(PDF)

Table S5 (A) GO analysis results for antagonism based target sets. (B) GO analysis results for target sets based on both antagonism and miRNA seed occurrence. (C) GO analysis results for correlation based target sets. (D) GO analysis results for target sets based on both correlation and miRNA seed occurrence.
(PDF)

Table S6 Comprehensive table with results of antagonism detection and seed enrichment for all genes and miRNAs included in the Ewing’s sarcoma study.
(TXT.GZ)

Table S7 List of miRNA hubs in common between the antagonism-based network and the correlation-based network.
(PDF)
