# Supplementary material for: Antagonism Pattern Detection between MicroRNA and Target Expression in Ewing’s Sarcoma
Source: PLoS One. 2012 Jul 25;7(7):e41770. doi: 10.1371/journal.pone.0041770 (PMC3404966; doi:10.1371/journal.pone.0041770)
Supplement: Figure S1 — False discovery rate (FDR) as a function of the Antagonism p-value obtained in Ewing’s sarcoma study. (PDF) [file pone.0041770.s001.pdf]

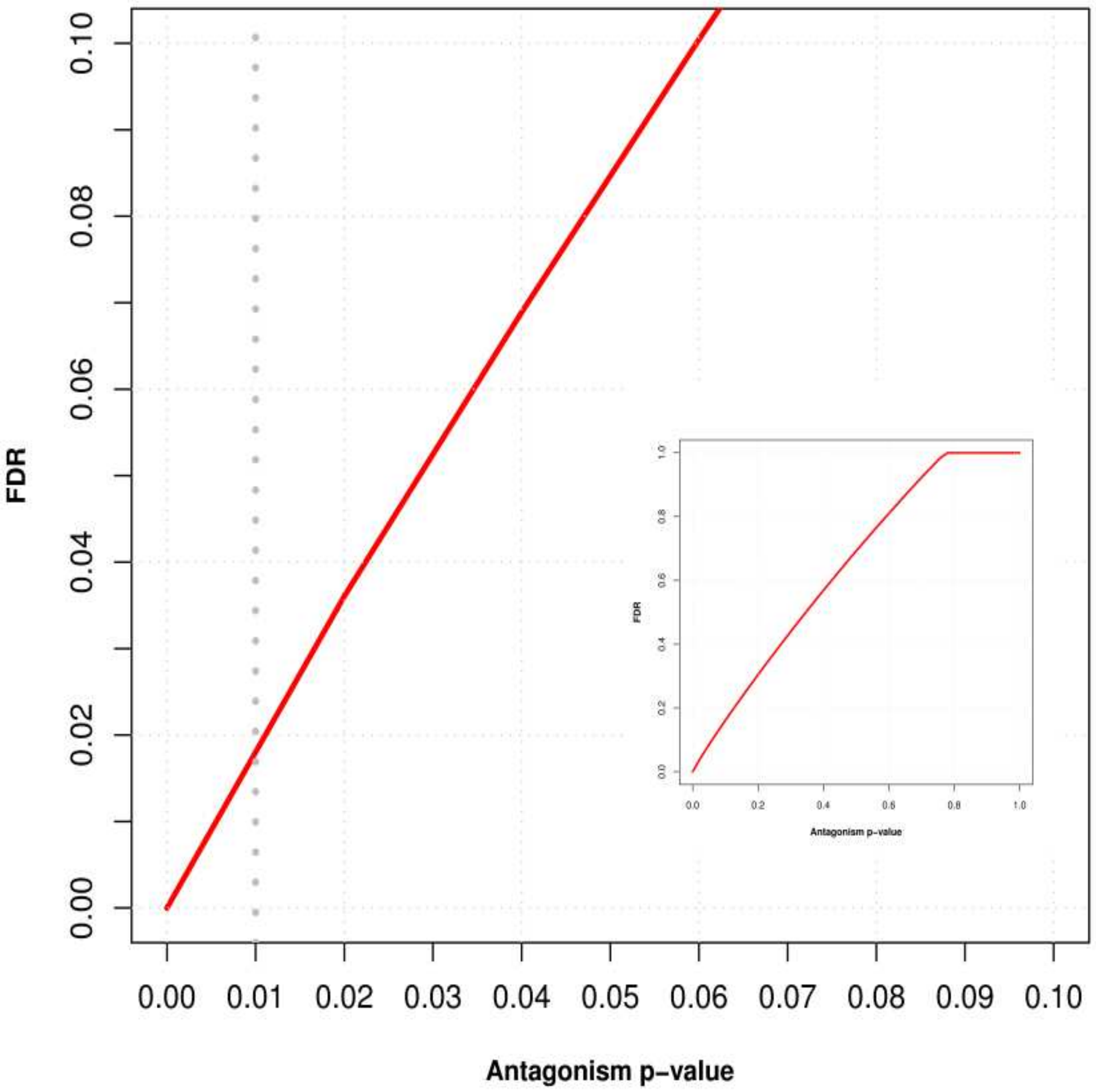

Supplementary Figure 1. Plot of the Antagonism p-value versus FDR used to pick the desired FDR level in Ewing's sarcoma study.
