# Supplementary figures and images for: Antagonism Pattern Detection between MicroRNA and Target Expression in Ewing’s Sarcoma
Source: PLoS One. 2012 Jul 25;7(7):e41770. doi: 10.1371/journal.pone.0041770 (PMC3404966; doi:10.1371/journal.pone.0041770)

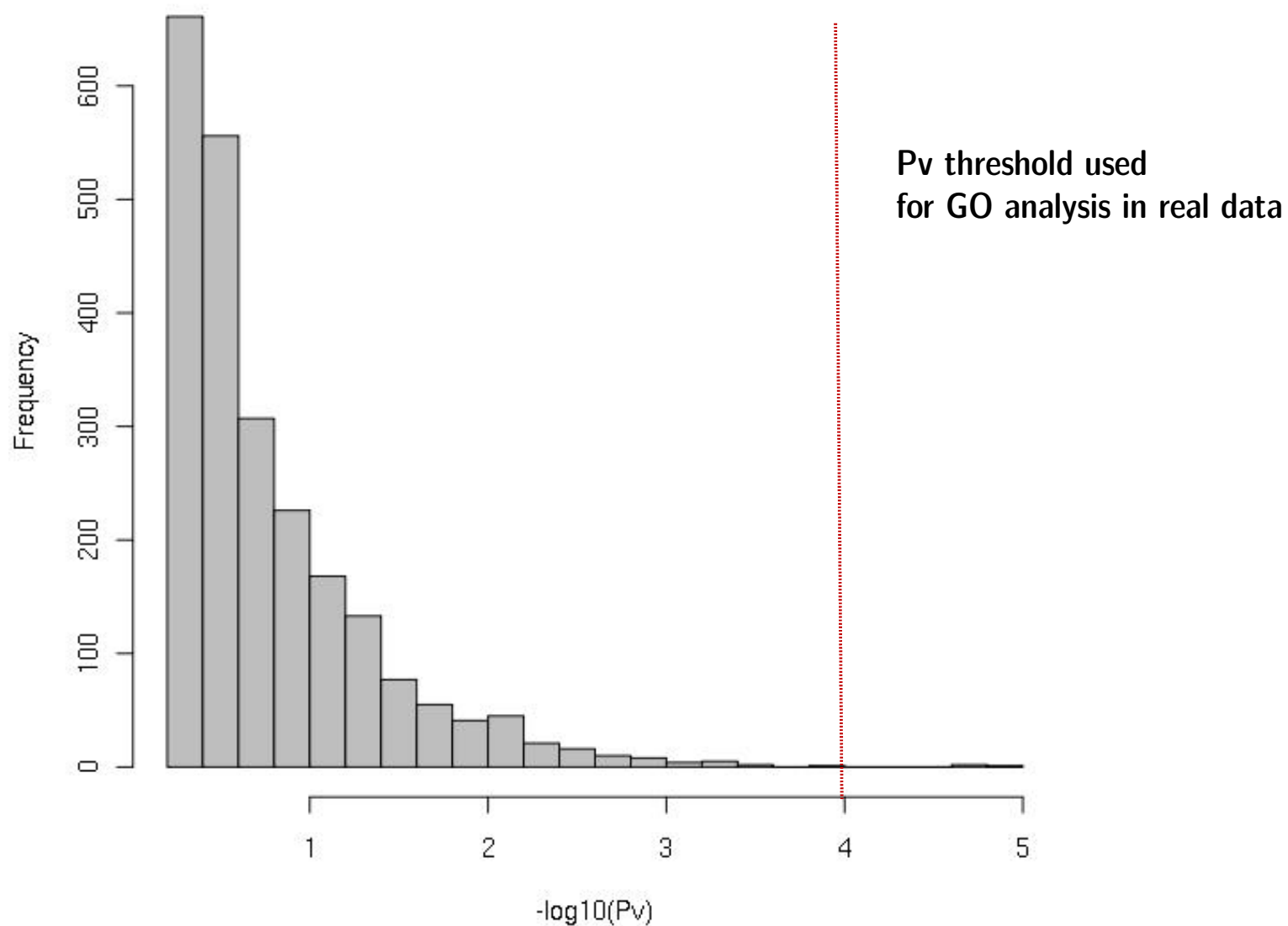

Supplementary Figure 2. GO enrichment p-value distribution in randomized target sets

Supplement: Figure S2 — GO enrichment p-value distribution for randomized target sets obtained by shuffling real miRNA-target relationships. The dotted vertical line is drawn at the p-value threshold used for GO analysis in real data. (PDF) [file pone.0041770.s002.pdf]
