# Supplementary material for: Antagonism Pattern Detection between MicroRNA and Target Expression in Ewing’s Sarcoma
Source: PLoS One. 2012 Jul 25;7(7):e41770. doi: 10.1371/journal.pone.0041770 (PMC3404966; doi:10.1371/journal.pone.0041770)
Supplement: Table S1 — Public datasets used for the test on experimentally validated miRNA targets. (PDF) [file pone.0041770.s003.pdf]

Data Source	Ref (PMID)	Description
GSE19536	21364938	miRNA-mRNA in primary breast tumors
GSE22058	20739924	miRNA-mRNA in Hepatocellular Carcinoma
GSE11508	18724358	miRNA-mRNA in Human Stem cells
GSE7055	18459106	miRNA-mRNA in Prostate cancer

Table S1. Public datasets used for the test on experimentally validated miRNA targets.
