# Supplementary material for: Antagonism Pattern Detection between MicroRNA and Target Expression in Ewing’s Sarcoma
Source: PLoS One. 2012 Jul 25;7(7):e41770. doi: 10.1371/journal.pone.0041770 (PMC3404966; doi:10.1371/journal.pone.0041770)
Supplement: Table S2 — Detailed results obtained for the test on experimentally validated targets. (PDF) [file pone.0041770.s004.pdf]

Table S2.Results obtained for the test on experimentally validated targets

miRNA|target	Pearson r	Correlation Pv	Antagonism Pv
hsa-let-7a|ACP1	-0.6231581	1.73453e-06	0.015
hsa-let-7a|BCL2	0.2486819	0.04576699	0.153
hsa-let-7a|CDC25A	0.1499639	0.2331259	0.139
hsa-let-7a|DAD1	0.4048024	0.003913119	0.149
hsa-let-7a|DICER1	-0.5424829	5.659068e-05	0.031
hsa-let-7a|HMGA1	-0.7058605	1.472359e-08	0.002
hsa-let-7a|HMGA2	-0.6106758	3.169111e-06	0.011
hsa-let-7a|KRAS	-0.6515853	3.965013e-07	0.057
hsa-let-7a|MED28	-0.3396184	0.005644565	0.088
hsa-let-7a|MYC	-0.1108327	0.3794358	0.132
hsa-let-7a|NKIRAS2	0.5095615	0.000184358	0.554
hsa-let-7a|NRAS	0.2388212	0.09841185	0.368
hsa-let-7a|RTCD1	0.05335366	0.715788	0.303
hsa-let-7a|SMOX	0.2716814	0.02858071	0.091
hsa-let-7a|TRIM71	-0.8048728	3.17775e-12	0.002
hsa-let-7a|VDR	0.2817042	0.02300467	0.212
hsa-let-7b|AARSD1	-0.4299215	0.002049008	0.123
hsa-let-7b|ACTG1	0.4905331	0.0003460044	0.219
hsa-let-7b|AKAP8	-0.4762661	0.000541851	0.166
hsa-let-7b|ANAPC1	-0.8425689	3.187934e-14	0.013
hsa-let-7b|ATP6V0A1	0.02082315	0.8692258	0.251
hsa-let-7b|ATP6V1F	0.4845633	0.0004184316	0.277
hsa-let-7b|AURKB	-0.7369839	1.562205e-09	0.001
hsa-let-7b|BAT2	0.1157728	0.2489509	0.175
hsa-let-7b|BCL7A	0.02568973	0.8390319	0.016
hsa-let-7b|CALCOCO2	0.1464228	0.2444731	0.403
hsa-let-7b|CAPG	-0.21248	0.1255239	0.048
hsa-let-7b|CARHSP1	-0.5281623	9.602204e-05	0.016
hsa-let-7b|CCND1	0.4569926	0.0001298562	0.024
hsa-let-7b|CDC25A	0.1728297	0.1685908	0.305
hsa-let-7b|CDC34	-0.1696679	0.2438211	0.048
hsa-let-7b|CDIPT	0.3402458	0.01675284	0.139
hsa-let-7b|CHMP2A	0.6359415	9.09921e-07	0.745
hsa-let-7b|COIL	-0.4415157	0.001494564	0.092
hsa-let-7b|COMMD9	0.6082117	3.558705e-06	0.79
hsa-let-7b|CSNK1D	0.2535318	0.07878956	0.283
hsa-let-7b|DSP	0.06182393	0.5390984	0.09
hsa-let-7b|DUSP12	-0.188746	0.1940043	0.159
hsa-let-7b|DUSP23	-0.3286795	0.02111988	0.124
hsa-let-7b|FADS2	0.161388	0.2679337	0.01
hsa-let-7b|FAM105A	-0.09866934	0.3262626	0.195
hsa-let-7b|FAM96A	-0.5907846	7.864086e-06	0.002
hsa-let-7b|FNDC3A	0.2192553	0.1301143	0.278
hsa-let-7b|GAK	-0.04136183	0.7778061	0.113
hsa-let-7b|GPR56	0.210882	0.08348758	0.084
hsa-let-7b|GRPEL2	-0.5426301	5.627699e-05	0.001
hsa-let-7b|GTPBP3	-0.674228	1.090983e-07	0.027
hsa-let-7b|GYS1	0.4436447	0.001408732	0.124
hsa-let-7b|HMGA1	-0.6355105	9.30374e-07	0.007
hsa-let-7b|HMGA2	-0.690907	3.915952e-08	0.008
hsa-let-7b|IFIT5	-0.1951339	0.1193021	0.212
hsa-let-7b|IFRD1	-0.1317821	0.3667351	0.018

hsa-let-7b|IPO4	-0.4465575	0.001298427	0.038
hsa-let-7b|KRAS	-0.5678222	2.088788e-05	0.032
hsa-let-7b|MLLT1	0.3920537	0.005335897	0.041
hsa-let-7b|MRPS24	0.06386812	0.6628505	0.268
hsa-let-7b|MRPS33	0.01062645	0.9422304	0.342
hsa-let-7b|MTPN	0.153691	0.2917255	0.152
hsa-let-7b|MTRR	-0.5289712	9.325548e-05	0.045
hsa-let-7b|NEDD4	0.1038191	0.4777693	0.205
hsa-let-7b|NXN	-0.4586419	0.0009186272	0.097
hsa-let-7b|PGRMC1	-0.4524381	0.0001544305	0.035
hsa-let-7b|POLD2	-0.08616277	0.5560856	0.039
hsa-let-7b|POLR2C	0.1588886	0.2755137	0.092
hsa-let-7b|POM121	-0.5518096	3.958203e-05	0.075
hsa-let-7b|PPP1R7	0.5888693	8.556173e-06	0.932
hsa-let-7b|PRIM1	-0.7258323	3.613743e-09	0.001
hsa-let-7b|PTGS2	0.06637355	0.6504617	0.129
hsa-let-7b|PXDN	-0.1918958	0.1835513	0.002
hsa-let-7b|RDH10	-0.1600528	0.1098617	0.099
hsa-let-7b|RHOB	0.11382	0.3666431	0.05
hsa-let-7b|RHOG	0.4743881	0.0005739804	0.915
hsa-let-7b|RPIA	-0.2696862	0.0609365	0.039
hsa-let-7b|SCYL1	0.2739952	0.05676855	0.448
hsa-let-7b|SLC1A4	0.45157	0.0001595735	0.058
hsa-let-7b|SLC25A1	0.3210604	0.02449207	0.283
hsa-let-7b|SLC25A13	-0.743042	9.730502e-10	0.003
hsa-let-7b|SLC25A24	0.02880255	0.7749407	0.261
hsa-let-7b|SLC25A32	-0.3755973	0.002048293	0.063
hsa-let-7b|SLC38A1	-0.8381271	5.82116e-14	0.011
hsa-let-7b|SNAP23	-0.2468753	0.08724803	0.046
hsa-let-7b|SPCS3	0.1782256	0.2204958	0.242
hsa-let-7b|SYPL1	-0.08897851	0.4809046	0.101
hsa-let-7b|TAF9B	0.4107805	0.003369495	0.126
hsa-let-7b|THBS1	0.1751004	0.2288275	0.033
hsa-let-7b|TMEM2	-0.5466819	4.824157e-05	0.001
hsa-let-7b|TPM2	0.2927338	0.02295941	0.14
hsa-let-7b|TRMT1	-0.6725198	1.207225e-07	0.021
hsa-let-7b|TYMS	-0.1112316	0.4467249	0.172
hsa-let-7b|UHRF1	-0.2161677	0.1357484	0.044
hsa-let-7b|VPS39	0.4516472	0.001124064	0.69
hsa-let-7c|BCL2	-0.01658407	0.8956806	0.169
hsa-let-7c|BCL2L1	0.1631323	0.2627268	0.215
hsa-let-7c|CDC25A	0.1221216	0.3324927	0.081
hsa-let-7c|HMGA2	-0.4896264	0.0003562174	0.031
hsa-let-7c|MED28	-0.03946782	0.7549286	0.231
hsa-let-7c|MYC	-0.07187291	0.5693893	0.059
hsa-let-7c|RTCD1	-0.2336847	0.1060884	0.025
hsa-let-7c|TRIM71	-0.5443716	5.268387e-05	0.007
hsa-let-7d|BCL2	0.1731053	0.1679024	0.047
hsa-let-7d|BDNF	-0.07065691	0.6294978	0.132
hsa-let-7d|CDC25A	0.171538	0.1718445	0.047
hsa-let-7d|DAD1	0.1509467	0.3005303	0.183
hsa-let-7d|DICER1	-0.4086574	0.003554423	0.029
hsa-let-7d|EIF4G2	0.2850334	0.04713135	0.288

Table S2.Results obtained for the test on experimentally validated targets

hsa-let-7d|HMGA2	-0.6109063	3.134776e-06	0.013
hsa-let-7d|KRAS	-0.3660493	0.009691195	0.036
hsa-let-7d|RABGAP1L	0.1662569	0.1856255	0.032
hsa-let-7d|SMOX	-0.1446392	0.2503297	0.077
hsa-let-7e|DAD1	0.378149	0.007384475	0.45
hsa-let-7e|HMGA2	-0.5492061	4.378243e-05	0.095
hsa-let-7e|RABGAP1L	-0.2289401	0.06659361	0.091
hsa-let-7f|ASS1	-0.3126599	0.02872207	0.061
hsa-let-7f|EEF1A2	0.211677	0.1442652	0.13
hsa-let-7f|ESM1	0.2317013	0.1091725	0.035
hsa-let-7f|FDPS	-0.2411962	0.09501088	0.046
hsa-let-7f|NTS	-0.1584335	0.2769087	0.096
hsa-let-7f|VIM	0.5453625	5.073465e-05	0.014
hsa-let-7g|BCL2L1	-0.03575803	0.8072909	0.03
hsa-let-7g|CDC25A	0.03991758	0.7522248	0.094
hsa-let-7g|COL1A2	-0.2228753	0.07433714	0.022
hsa-let-7g|DAD1	0.2986642	0.0371131	0.378
hsa-let-7g|EIF4G2	0.04769798	0.7448391	0.167
hsa-let-7g|HMGA2	-0.6924704	3.54496e-08	0.002
hsa-let-7g|KRAS	-0.3957401	0.004884194	0.028
hsa-let-7g|MYC	-0.09076987	0.5110749	0.101
hsa-let-7i|DAD1	0.3634173	0.01026808	0.313
hsa-let-7i|EIF4G2	0.158574	0.2764775	0.252
hsa-miR-100|EGR2	0.196477	0.1685211	0.125
hsa-miR-100|FGFR3	-0.6839836	6.402719e-09	0.001
hsa-miR-100|ID1	-0.7504285	5.365552e-10	0.013
hsa-miR-100|IGF1R	0.259781	0.036635	0.026
hsa-miR-100|PLK1	-0.6646694	1.90644e-07	0.006
hsa-miR-101|APP	0.3368028	0.01796431	0.112
hsa-miR-101|ATXN1	-0.2341479	0.0604741	0.024
hsa-miR-101|EZH2	0.2414747	0.05264294	0.031
hsa-miR-101|FOS	0.305379	0.01337316	0.114
hsa-miR-101|MCL1	0.3142049	0.0108045	0.048
hsa-miR-101|MYCN	0.427625	0.0009290042	0.021
hsa-miR-103|GPD1	-0.04752446	0.7457363	0.409
hsa-miR-103|SERBP1	0.4538573	0.0001463499	0.048
hsa-miR-105|TLR2	-0.1048829	0.1476713	0.05
hsa-miR-106a|APP	0.4182501	0.002784466	0.594
hsa-miR-106a|CDKN1A	-0.6188423	2.142738e-06	0.005
hsa-miR-106a|RB1	-0.1525473	0.2953743	0.179
hsa-miR-106b|APP	0.3553162	0.01223298	0.001
hsa-miR-106b|CDKN1A	-0.5259601	0.000103938	0.006
hsa-miR-106b|ITCH	0.4990773	0.0002619916	0.001
hsa-miR-106b|PCAF	-0.2740491	0.0001198467	0.003
hsa-miR-107|BACE1	-0.04100973	0.7796501	0.062
hsa-miR-107|CCNE1	-0.1790289	0.2183887	0.187
hsa-miR-107|CDCA4	-0.2066477	0.1542669	0.257
hsa-miR-107|HIF1A	-0.2083714	0.1507832	0.063
hsa-miR-107|RAB1B	-0.2889339	0.02927235	0.121
hsa-miR-107|SERBP1	-0.2420301	0.09383859	0.018
hsa-miR-10a|USF2	0.2489055	0.000498861	0.142
hsa-miR-10b|HOXD10	-0.3754918	0.002054735	0.043
hsa-miR-10b|KLF4	0.1602604	0.02638452	0.04

Table S2.Results obtained for the test on experimentally validated targets

hsa-miR-124a|CDK2	0.2485469	0.08506008	0.261
hsa-miR-124a|NR3C1	-0.3263581	0.02210314	0.182
hsa-miR-125a|ARID3B	-0.7109822	1.038554e-08	0.035
hsa-miR-125a|ERBB2	-0.05958108	0.5208977	0.273
hsa-miR-125a|ERBB3	-0.7265441	4.284935e-12	0.001
hsa-miR-125a|LIF	0.3179173	0.02600911	0.475
hsa-miR-125a|RARA	0.354925	0.01233551	0.31
hsa-miR-125b|ADAMTS1	0.5258407	4.884981e-15	0.182
hsa-miR-125b|BAK1	-0.02426557	0.8685518	0.198
hsa-miR-125b|CASP6	-0.2369892	0.05733215	0.046
hsa-miR-125b|CASP7	-0.2787363	0.02455045	0.021
hsa-miR-125b|CBX7	0.1033495	0.1537171	0.061
hsa-miR-125b|CDC25A	0.08806913	0.4854172	0.254
hsa-miR-125b|CDK6	-0.07836291	0.279964	0.096
hsa-miR-125b|CDKN2A	-0.5016648	1.241865e-13	0.004
hsa-miR-125b|CEBPG	-0.4504165	8.032627e-10	0.003
hsa-miR-125b|CLU	0.4050517	5.62243e-09	0.239
hsa-miR-125b|CYP1A1	0.2573451	0.0003139886	0.351
hsa-miR-125b|DICER1	-0.5581528	3.084876e-05	0.006
hsa-miR-125b|EIF4EBP1	-0.3045566	0.009769531	0.021
hsa-miR-125b|ERBB2	0.06835033	0.3461887	0.522
hsa-miR-125b|ERBB3	-0.6663427	3.549734e-20	0.001
hsa-miR-125b|GPR160	-0.7859238	2.238764e-11	0.025
hsa-miR-125b|H3F3B	0.1950851	0.1791779	0.046
hsa-miR-125b|HMGA1	-0.5778549	1.702192e-18	0.001
hsa-miR-125b|HMGA2	-0.4876672	0.0003792243	0.043
hsa-miR-125b|HOMER2	0.1156929	0.1100452	0.071
hsa-miR-125b|ID1	-0.7558865	5.444978e-12	0.025
hsa-miR-125b|ID2	0.4890366	6.148415e-13	0.015
hsa-miR-125b|ID3	0.5102647	4.019007e-14	0.106
hsa-miR-125b|IGFBP3	0.4436844	1.152158e-10	0.044
hsa-miR-125b|IL1RN	0.2360832	0.0009785309	0.116
hsa-miR-125b|KLF13	-0.4239697	8.907559e-10	0.001
hsa-miR-125b|LIF	0.2509828	0.08194883	0.391
hsa-miR-125b|MAN1A1	0.2395233	0.0008195596	0.129
hsa-miR-125b|NKIRAS2	0.5438102	5.381848e-05	0.398
hsa-miR-125b|ODZ2	0.183365	0.01090243	0.409
hsa-miR-125b|PERP	0.1906387	0.008081323	0.181
hsa-miR-125b|PIGR	0.2672777	0.0001784376	0.001
hsa-miR-125b|RBM8A	-0.3636073	2.172585e-07	0.008
hsa-miR-125b|SGPL1	-0.1390221	0.05446545	0.083
hsa-miR-125b|SMO	-0.2497887	0.0004756154	0.001
hsa-miR-125b|TP53	-0.1949933	0.1195737	0.096
hsa-miR-125b|TSPAN8	-0.3176039	0.009936101	0.007
hsa-miR-125b|UBE2I	-0.4146308	0.002064801	0.051
hsa-miR-125b|UGT2B15	0.4537997	3.829737e-11	0.115
hsa-miR-125b|VDR	-0.3884284	0.001386728	0.043
hsa-miR-126|CCNE2	-0.1053023	0.4038157	0.026
hsa-miR-126|CRK	0.3346693	0.01094159	0.069
hsa-miR-126|HOXA9	-0.2578389	0.0381135	0.045
hsa-miR-126|IRS1	0.1842435	0.1417768	0.076
hsa-miR-126|PLK2	0.5403142	6.140316e-05	0.072
hsa-miR-126|RGS3	0.3280045	0.02140193	0.028

Table S2.Results obtained for the test on experimentally validated targets

hsa-miR-126*|SLC45A3	-0.2253663	0.0234547	0.162
hsa-miR-126|SPRED1	-0.6110765	3.109628e-06	0.068
hsa-miR-126|TOM1	-0.1851059	0.2029014	0.228
hsa-miR-126|VCAM1	0.1290987	0.3385201	0.07
hsa-miR-126|VEGFA	-0.2121863	0.08971762	0.041
hsa-miR-127|BCL6	0.3676859	0.009346755	0.11
hsa-miR-127|XBP1	0.09023832	0.2768541	0.248
hsa-miR-128b|CDKN1B	0.4520484	0.001111255	0.164
hsa-miR-128b|EGFR	-0.08582012	0.4966727	0.021
hsa-miR-128b|MLL	-0.3419436	0.00530477	0.007
hsa-miR-129|GALNT1	0.3531607	0.003906228	0.282
hsa-miR-129|NOTCH1	0.1674018	0.1825723	0.06
hsa-miR-129|SOX4	-0.5438752	2.836423e-06	0.001
hsa-miR-130a|ATXN1	0.1423217	0.06587022	0.018
hsa-miR-130a|HOXA5	0.07495037	0.5795058	0.19
hsa-miR-130a|MAFB	0.2234703	0.07354707	0.108
hsa-miR-130a|NR3C2	0.1775533	0.02477174	0.041
hsa-miR-130a|TAC1	-0.1454153	0.2477698	0.52
hsa-miR-130b|RUNX3	-0.1961121	0.1174249	0.087
hsa-miR-130b|TP53INP1	0.11121	0.3894189	0.05
hsa-miR-132|CDKN1A	0.310672	0.02980746	0.204
hsa-miR-132|MECP2	-0.3219216	0.008920927	0.215
hsa-miR-132|RFX4	0.5219887	0.0001197348	0.047
hsa-miR-132|SIRT1	-0.1618853	0.2664422	0.158
hsa-miR-133a|CDC42	0.1000841	0.1672111	0.121
hsa-miR-133a|ERG	0.3586912	3.243089e-07	0.426
hsa-miR-133a|FSCN1	-0.09086345	0.2100552	0.136
hsa-miR-133a|KLF15	-0.01505819	0.8357743	0.068
hsa-miR-133a|KRT7	0.2299344	0.001334952	0.44
hsa-miR-133a|PKM2	-0.1885394	0.008820102	0.217
hsa-miR-133a|RHOA	-0.1982613	0.005839345	0.035
hsa-miR-133a|UCP2	0.0779702	0.2823885	0.204
hsa-miR-133b|BCL2L2	-0.2822754	7.285828e-05	0.065
hsa-miR-133b|KLF15	-0.05265717	0.4682201	0.061
hsa-miR-133b|MCL1	0.5463091	2.508197e-06	0.19
hsa-miR-133b|PKM2	-0.1603728	0.0262777	0.123
hsa-miR-135a|APC	0.1601558	0.2716531	0.067
hsa-miR-135a|NR3C2	-0.3370263	1.752329e-06	0.001
hsa-miR-135b|APC	0.6254547	1.547999e-06	0.027
hsa-miR-137|CDK6	-0.0131021	0.8568619	0.046
hsa-miR-137|NCOA2	0.3945184	0.00114602	0.058
hsa-miR-138|ARHGEF3	0.2382757	0.0008742412	0.165
hsa-miR-138|RHOC	-0.229659	0.001353402	0.003
hsa-miR-138|ROCK2	-0.1059507	0.143569	0.243
hsa-miR-140|CXCL12	-0.5206943	0.0001253385	0.069
hsa-miR-140|HDAC4	0.1176058	0.4209428	0.149
hsa-miR-141|BAP1	-0.09748347	0.1785748	0.001
hsa-miR-141|BRD3	-0.09753425	0.5049593	0.044
hsa-miR-141|DLX5	-0.1769701	0.2238175	0.269
hsa-miR-141|ELMO2	0.1038903	0.151564	0.255
hsa-miR-141|ERBB2IP	-0.7593544	2.541148e-10	0.001
hsa-miR-141|HMGB1	-0.09293136	0.1998208	0.041
hsa-miR-141|KLF5	0.355797	4.092884e-07	0.163

Table S2.Results obtained for the test on experimentally validated targets

hsa-miR-141|PTEN	-0.0555048	0.4444713	0.142
hsa-miR-141|SERBP1	0.5947104	6.604362e-06	0.027
hsa-miR-141|SFPQ	0.2625168	0.0684116	0.033
hsa-miR-141|STK3	-0.02533947	0.862789	0.112
hsa-miR-141|TGFB2	-0.3503037	0.01360354	0.101
hsa-miR-141|UBAP1	-0.5366336	7.043539e-05	0.013
hsa-miR-141|ZEB1	-0.3559014	0.01208096	0.071
hsa-miR-141|ZEB2	-0.1808074	0.02548856	0.105
hsa-miR-141|ZFHX1B	-0.1018293	0.3109312	0.061
hsa-miR-143|DNMT3A	-0.3506425	6.159756e-07	0.182
hsa-miR-143|ELK1	0.06402099	0.662092	0.381
hsa-miR-143|FNDC3B	0.5833707	1.08681e-05	0.056
hsa-miR-143|KLF4	0.2989176	0.0009660595	0.277
hsa-miR-143|KRAS	0.1714961	0.2305563	0.097
hsa-miR-143|MYO6	-0.2324533	0.0309112	0.033
hsa-miR-145|ACBD3	0.6569536	2.948682e-07	0.128
hsa-miR-145|BNIP3	0.2833011	0.04854898	0.003
hsa-miR-145|CBFB	0.3692756	1.357465e-07	0.157
hsa-miR-145|CCDC25	-0.4548044	0.001026731	0.034
hsa-miR-145|CCNA2	-0.3494647	0.0138453	0.023
hsa-miR-145|CDKN1A	0.3724567	0.002104753	0.129
hsa-miR-145|CLINT1	0.638011	8.174064e-07	0.068
hsa-miR-145|ELK1	0.4893025	0.0003599316	0.143
hsa-miR-145|FBXO28	-0.3215282	0.02427276	0.015
hsa-miR-145|FLI1	0.5773037	9.960166e-11	0.008
hsa-miR-145|FSCN1	0.1606758	0.2010378	0.071
hsa-miR-145|HOXA9	-0.2016059	0.1073009	0.069
hsa-miR-145|IGF1R	0.1875219	0.1624678	0.135
hsa-miR-145|IRS1	-0.198779	0.005709503	0.026
hsa-miR-145|KLF4	0.6794414	7.976702e-08	0.023
hsa-miR-145|KLF5	0.161362	0.08188985	0.073
hsa-miR-145|KRT7	0.1744363	0.01552653	0.283
hsa-miR-145|MUC1	-0.07557581	0.5496092	0.333
hsa-miR-145|MYC	0.4894228	0.0003585481	0.02
hsa-miR-145|MYO6	-0.2510273	0.0004447019	0.032
hsa-miR-145|PPP3CA	0.3792073	5.824831e-08	0.054
hsa-miR-145|RASA1	0.6502812	4.257028e-07	0.201
hsa-miR-145|RTKN	-0.328272	0.0001936076	0.002
hsa-miR-145|SOX2	-0.01248093	0.9213956	0.246
hsa-miR-145|STAT1	0.6185424	2.174181e-06	0.041
hsa-miR-145|TMOD3	-0.1421678	0.1079645	0.237
hsa-miR-145|USP46	0.113604	0.3675589	0.069
hsa-miR-145|YES1	-0.2485956	0.06222843	0.045
hsa-miR-146a|ATOH8	0.2847596	0.001874313	0.055
hsa-miR-146a|BLMH	0.08837268	0.2228754	0.125
hsa-miR-146a|BRCA1	0.1933897	0.1227056	0.102
hsa-miR-146a|BRCA2	0.1033395	0.4126837	0.2
hsa-miR-146a|CCNA2	0.2959142	0.01669698	0.054
hsa-miR-146a|CDKN3	-0.2515377	0.0004325132	0.014
hsa-miR-146a|CFH	0.1619444	0.2662653	0.022
hsa-miR-146a|COL13A1	-0.1113235	0.2677278	0.225
hsa-miR-146a|CXCR4	0.4334456	3.387988e-10	0.027
hsa-miR-146a|ELL3	-0.3464057	8.573364e-07	0.019

Table S2.Results obtained for the test on experimentally validated targets

hsa-miR-146a|FADD	-0.003001539	0.9670415	0.024
hsa-miR-146a|FAF1	-0.2869068	5.467421e-05	0.061
hsa-miR-146a|IL8	0.7103814	1.082376e-08	0.105
hsa-miR-146a|IRAK1	0.5268933	0.0001005135	0.001
hsa-miR-146a|IRAK2	-0.03998668	0.5818572	0.223
hsa-miR-146a|KIF22	0.2159961	0.083972	0.048
hsa-miR-146a|KIT	0.08606238	0.3921418	0.252
hsa-miR-146a|LTB	0.1066202	0.2885943	0.223
hsa-miR-146a|MCM10	-0.276103	0.05481505	0.246
hsa-miR-146a|METTL7A	-0.3426984	1.14045e-06	0.001
hsa-miR-146a|MR1	-0.2028753	0.1050606	0.108
hsa-miR-146a|NFIX	0.1421369	0.1562235	0.041
hsa-miR-146a|PA2G4	-0.1755517	0.02012274	0.014
hsa-miR-146a|PBLD	0.1439221	0.04641482	0.019
hsa-miR-146a|PDIK1L	-0.2837648	0.04816616	0.133
hsa-miR-146a|PEX11G	0.1053686	0.1457942	0.08
hsa-miR-146a|PLEKHA4	-0.1482018	0.3095068	0.02
hsa-miR-146a|POLE2	-0.3247429	4.31604e-06	0.028
hsa-miR-146a|RAD54L	-0.154775	0.1371114	0.082
hsa-miR-146a|ROCK1	0.02202528	0.8617483	0.242
hsa-miR-146a|SDCBP2	0.07306168	0.4677821	0.165
hsa-miR-146a|STAT1	0.5986779	1.338272e-10	0.029
hsa-miR-146a|TBP	-0.3343969	2.132286e-06	0.03
hsa-miR-146a|TIMELESS	-0.2198068	0.002188389	0.001
hsa-miR-146a|TLR2	0.3419869	1.204147e-06	0.82
hsa-miR-146a|TMSL8	-0.01356267	0.892918	0.06
hsa-miR-146a|TRIM14	0.336592	1.810294e-06	0.213
hsa-miR-146a|UHRF1	-0.4064178	0.003759119	0.12
hsa-miR-146a|VWCE	-0.08306131	0.4089211	0.256
hsa-miR-146b|MMP16	-0.04303823	0.7335474	0.119
hsa-miR-148a|DNMT1	0.6248662	7.92931e-11	0.004
hsa-miR-148a|DNMT3B	0.668204	1.554565e-07	0.05
hsa-miR-148a|TGIF2	0.4885141	2.191562e-07	0.03
hsa-miR-150|CCNE1	-0.2890209	4.787799e-05	0.016
hsa-miR-150|EGR2	0.437572	2.203091e-10	0.113
hsa-miR-150|MYB	-0.02614408	0.795235	0.147
hsa-miR-152|DNMT1	-0.4906874	0.0003442931	0.02
hsa-miR-152|FOXO1	-0.3488771	0.01401682	0.074
hsa-miR-153|FOXO1	-0.2211483	0.126748	0.179
hsa-miR-153|MCL1	-0.1271706	0.3838871	0.192
hsa-miR-155|AGTR1	-0.09403039	0.4562471	0.038
hsa-miR-155|AMIGO2	0.2906589	0.04275849	0.063
hsa-miR-155|ANKFY1	-0.1497142	0.3045402	0.124
hsa-miR-155|APC	-0.2525928	0.07994202	0.149
hsa-miR-155|ARFIP1	-0.03627361	0.774214	0.196
hsa-miR-155|ARFIP2	-0.02943504	0.770134	0.196
hsa-miR-155|ARID2	-0.5381865	6.648567e-05	0.015
hsa-miR-155|ARNTL	-0.4791963	0.0004949488	0.032
hsa-miR-155|ATG3	0.2752449	0.05560361	0.207
hsa-miR-155|ATP6V1C1	-0.101918	0.4191754	0.054
hsa-miR-155|BACH1	0.02330387	0.8737182	0.126
hsa-miR-155|BET1	0.04893475	0.7384546	0.276
hsa-miR-155|BRPF3	0.04157671	0.7766814	0.155

Table S2.Results obtained for the test on experimentally validated targets

hsa-miR-155|CBFB	0.5015867	0.0002411035	0.298
hsa-miR-155|CCND1	0.1676901	0.1073844	0.181
hsa-miR-155|CDK5RAP3	-0.2071263	0.1532936	0.271
hsa-miR-155|CEBPB	-0.07737411	0.5401239	0.113
hsa-miR-155|CHAF1A	-0.4327653	0.001898347	0.067
hsa-miR-155|CLDN1	0.1831291	0.1442444	0.111
hsa-miR-155|CSF1R	0.2352882	0.01785853	0.21
hsa-miR-155|CTNNB1	-0.3050201	0.03308129	0.093
hsa-miR-155|CUL4B	0.4206671	0.002615413	0.111
hsa-miR-155|CYP51A1	-0.2881957	0.04463052	0.091
hsa-miR-155|CYR61	0.557439	3.173444e-05	0.154
hsa-miR-155|DHX40	-0.3327499	0.0194848	0.125
hsa-miR-155|DNAJB1	0.04308275	0.7688111	0.154
hsa-miR-155|DNAJC19	-0.4107226	0.003374427	0.049
hsa-miR-155|DPP7	0.1118124	0.4443404	0.242
hsa-miR-155|DSG2	-0.3900856	0.005591681	0.14
hsa-miR-155|FADS1	-0.1246549	0.2915977	0.049
hsa-miR-155|FADS3	0.4920713	0.0003292821	0.191
hsa-miR-155|FMNL2	-0.1618763	0.2664692	0.11
hsa-miR-155|FOXO3	-0.3207154	0.02465491	0.057
hsa-miR-155|GNA13	-0.4238596	0.002406067	0.03
hsa-miR-155|HIF1A	-0.08460686	0.5632662	0.191
hsa-miR-155|HIVEP2	-0.06980698	0.4878917	0.054
hsa-miR-155|HSD17B12	-0.2744379	0.05635362	0.061
hsa-miR-155|JARID2	-0.5177574	0.0001389492	0.015
hsa-miR-155|KIAA0776	0.08800514	0.4857355	0.024
hsa-miR-155|LDOC1	0.2855967	0.04667777	0.039
hsa-miR-155|LPL	-0.2820316	0.04960969	0.095
hsa-miR-155|LY6K	0.03532605	0.7258038	0.224
hsa-miR-155|MATR3	-0.5232042	0.0001146817	0.028
hsa-miR-155|MECP2	0.1048064	0.4060456	0.312
hsa-miR-155|MEIS1	-0.1616237	0.1063723	0.044
hsa-miR-155|MET	0.2778624	0.02502201	0.117
hsa-miR-155|METTL7A	-0.3603769	0.01097106	0.093
hsa-miR-155|MLH1	-0.3000942	0.03617238	0.212
hsa-miR-155|MOSC1	-0.6301582	1.22272e-06	0.035
hsa-miR-155|MPZL1	0.3030854	0.03426818	0.169
hsa-miR-155|MSH2	-0.422895	0.0024677	0.165
hsa-miR-155|MSH6	-0.4993733	0.0002594447	0.027
hsa-miR-155|MSI2	0.06636399	0.5096546	0.112
hsa-miR-155|MYO10	0.09191875	0.4664684	0.141
hsa-miR-155|NARS	-0.5418426	5.797436e-05	0.04
hsa-miR-155|NT5E	0.3119921	0.01140458	0.111
hsa-miR-155|PDLIM5	0.1317045	0.295655	0.134
hsa-miR-155|PHC2	0.07565672	0.6053887	0.329
hsa-miR-155|PHF17	-0.3299902	0.02058118	0.18
hsa-miR-155|PICALM	0.3062056	0.03237102	0.038
hsa-miR-155|PLXND1	0.7086313	1.220132e-08	0.071
hsa-miR-155|PODXL	-0.349007	0.01397874	0.179
hsa-miR-155|POLE3	-0.01907271	0.8965078	0.022
hsa-miR-155|POLE4	0.3147075	0.02763867	0.523
hsa-miR-155|PPL	0.2224291	0.0749341	0.193
hsa-miR-155|PRAF2	0.1511075	0.3000099	0.029

hsa-miR-155|RAB23	0.1182886	0.418232	0.109
hsa-miR-155|RAB34	0.1264249	0.2100639	0.051
hsa-miR-155|RAB5C	0.1265608	0.3861902	0.217
hsa-miR-155|RAB6A	-0.03066003	0.8343477	0.156
hsa-miR-155|RAI14	0.6122904	2.935704e-06	0.09
hsa-miR-155|RCN2	-0.4369425	0.001694872	0.04
hsa-miR-155|RCOR1	-0.03916061	0.7567772	0.25
hsa-miR-155|RHEB	-0.07351535	0.6156655	0.332
hsa-miR-155|RHOA	0.5387719	6.505025e-05	0.583
hsa-miR-155|SDCBP	-0.01813255	0.9015835	0.06
hsa-miR-155|SH3BP4	0.2602205	0.03630711	0.122
hsa-miR-155|SLA	-0.129819	0.3739794	0.197
hsa-miR-155|SMAD1	-0.06188117	0.6727396	0.229
hsa-miR-155|SMAD5	-0.1036844	0.4111171	0.283
hsa-miR-155|SOCS1	-0.3221705	0.02397435	0.124
hsa-miR-155|SYPL1	-0.1050102	0.4051282	0.105
hsa-miR-155|TACSTD2	0.1545049	0.1229019	0.581
hsa-miR-155|TBCA	-0.2077991	0.1519334	0.151
hsa-miR-155|TM6SF1	0.6114133	3.060444e-06	0.06
hsa-miR-155|TP53INP1	0.08661912	0.5539878	0.075
hsa-miR-155|TRAM1	0.2954821	0.03927876	0.005
hsa-miR-155|TRIP13	-0.2051985	0.1572413	0.105
hsa-miR-155|TXNDC12	-0.3632924	0.01029618	0.104
hsa-miR-155|UBE2J1	0.7149884	7.862794e-09	0.269
hsa-miR-155|VAMP3	0.5994744	5.326874e-06	0.055
hsa-miR-155|WDFY1	0.2727117	0.05798528	0.122
hsa-miR-155|ZIC3	-0.4456102	0.001333423	0.092
hsa-miR-155|ZNF652	-0.1949509	0.1794832	0.081
hsa-miR-15a|ACTR1A	0.08883	0.5438777	0.053
hsa-miR-15a|BACE1	0.02922633	0.8419925	0.155
hsa-miR-15a|BCL2	0.1050918	0.4047612	0.104
hsa-miR-15a|BMI1	0.5203866	0.000126705	0.242
hsa-miR-15a|BRCA1	-0.116257	0.3564034	0.194
hsa-miR-15a|CCND1	-0.6366112	8.789675e-07	0.009
hsa-miR-15a|CCND2	0.4342908	0.001821649	0.008
hsa-miR-15a|CCNE1	0.1522423	0.2260208	0.125
hsa-miR-15a|CDC14B	-0.3575799	0.01165392	0.042
hsa-miR-15a|CDC25A	-0.1797909	0.1518287	0.132
hsa-miR-15a|CENPJ	0.08023591	0.5836664	0.153
hsa-miR-15a|DMTF1	0.1022852	0.4843332	0.195
hsa-miR-15a|ECHDC1	-0.1144846	0.4334599	0.395
hsa-miR-15a|GOLGA5	-0.1683462	0.2475678	0.212
hsa-miR-15a|GOLPH3L	-0.1623302	0.2651127	0.084
hsa-miR-15a|H3F3B	0.37756	0.007484577	0.052
hsa-miR-15a|HDHD2	0.3529504	0.01286433	0.241
hsa-miR-15a|HERC6	0.02957679	0.8150781	0.07
hsa-miR-15a|HRSP12	0.1253111	0.3909355	0.345
hsa-miR-15a|HSDL2	0.6560741	3.096522e-07	0.044
hsa-miR-15a|HSPA1A	0.1654295	0.1878549	0.414
hsa-miR-15a|JUN	0.07498899	0.5801002	0.074
hsa-miR-15a|MCL1	-0.3901632	0.005581402	0.024
hsa-miR-15a|MSH2	-0.1800174	0.2158152	0.154
hsa-miR-15a|PDCD4	0.1470254	0.3134059	0.201

Table S2.Results obtained for the test on experimentally validated targets

hsa-miR-15a|PNN	0.05449326	0.6663704	0.162
hsa-miR-15a|PRIM1	-0.08842667	0.5457154	0.125
hsa-miR-15a|RAB21	0.3398401	0.01689188	0.115
hsa-miR-15a|RAD51C	0.3406888	0.01660213	0.183
hsa-miR-15a|SLC35A1	0.08766914	0.5491751	0.199
hsa-miR-15a|SLC35B3	-0.2069095	0.153734	0.072
hsa-miR-15a|TIA1	0.4920698	0.0003292984	0.155
hsa-miR-15a|TPI1	-0.3598963	0.0110859	0.079
hsa-miR-15a|UGDH	-0.1860567	0.2005503	0.016
hsa-miR-15a|UGP2	-0.3967717	0.004763938	0.036
hsa-miR-15b|BCL2	-0.1753626	0.1623415	0.032
hsa-miR-15b|CASP8	0.08552242	0.4981726	0.027
hsa-miR-15b|CCNE1	-0.2236573	0.122388	0.101
hsa-miR-15b|RECK	-0.4366234	0.0017097	0.033
hsa-miR-16|ABHD10	0.2414288	0.09468274	0.142
hsa-miR-16|ACTR1A	-0.03937656	0.7882187	0.031
hsa-miR-16|ACVR2A	-0.1189218	0.3454107	0.036
hsa-miR-16|ARHGDIA	-0.5483799	4.519843e-05	0.038
hsa-miR-16|ARL2	-0.1365916	0.3493481	0.068
hsa-miR-16|ATG9A	0.4358114	0.0002849144	0.382
hsa-miR-16|BCL2	-0.2134465	0.08778383	0.009
hsa-miR-16|BMI1	0.3099004	0.03023786	0.267
hsa-miR-16|BRCA1	-0.04596773	0.716153	0.214
hsa-miR-16|CA12	-0.0892593	0.541925	0.083
hsa-miR-16|CAPRIN1	0.4469253	0.0001898617	0.159
hsa-miR-16|CASP8	0.1832258	0.1440289	0.366
hsa-miR-16|CCND1	-0.7950686	8.950798e-12	0.001
hsa-miR-16|CCND3	-0.312367	0.02887989	0.082
hsa-miR-16|CCNE1	-0.2128561	0.0886857	0.161
hsa-miR-16|CCNT2	0.4651008	0.0007595191	0.617
hsa-miR-16|CDC14B	-0.2501997	0.08293912	0.181
hsa-miR-16|CDK1	0.2076667	0.1522003	0.117
hsa-miR-16|CDK2	0.4719117	0.0006189677	0.355
hsa-miR-16|CDK5RAP1	0.2007377	0.1666609	0.234
hsa-miR-16|CENPJ	0.2887924	0.04417099	0.303
hsa-miR-16|CFL2	0.2106717	0.1462249	0.17
hsa-miR-16|CHORDC1	0.1524681	0.2956281	0.287
hsa-miR-16|CRHBP	-0.6911752	3.84982e-08	0.094
hsa-miR-16|DNAJB4	0.0159794	0.899464	0.225
hsa-miR-16|ECHDC1	-0.002402306	0.9869296	0.237
hsa-miR-16|EGFR	-0.04575031	0.7174391	0.254
hsa-miR-16|EIF4E	-0.3617536	0.00306696	0.049
hsa-miR-16|FGF2	-0.3737242	0.002165506	0.028
hsa-miR-16|FNDC3B	0.09752969	0.5049793	0.21
hsa-miR-16|GALNT7	0.133658	0.2884864	0.158
hsa-miR-16|GFM1	0.3518491	0.01316765	0.531
hsa-miR-16|GFPT1	-0.1616051	0.267282	0.014
hsa-miR-16|GNL3L	0.2868471	0.04568342	0.208
hsa-miR-16|GOLGA5	-0.274048	0.05671894	0.047
hsa-miR-16|GOLPH3L	0.04891314	0.738566	0.137
hsa-miR-16|GSTM4	0.3296078	0.007335984	0.344
hsa-miR-16|H3F3B	0.1968858	0.1751192	0.033
hsa-miR-16|HARS	-0.1933217	0.1832179	0.04

Table S2.Results obtained for the test on experimentally validated targets

hsa-miR-16|HARS2	0.6322824	1.097742e-06	0.875
hsa-miR-16|HBXIP	0.2561913	0.07559634	0.756
hsa-miR-16|HDHD2	0.3341921	0.01893171	0.583
hsa-miR-16|HERC6	-0.006811877	0.9570514	0.25
hsa-miR-16|HMGA1	-0.2493162	0.04519902	0.016
hsa-miR-16|HMOX1	0.1263783	0.386881	0.221
hsa-miR-16|HRSP12	0.1964326	0.1761345	0.276
hsa-miR-16|HSDL2	0.4340485	0.001833644	0.019
hsa-miR-16|HSPA1A	0.1851529	0.2027848	0.172
hsa-miR-16|HSPA1B	0.3767001	0.007632842	0.135
hsa-miR-16|IFRD1	-0.2530299	0.04198863	0.057
hsa-miR-16|IFRD2	-0.3628225	0.01040248	0.129
hsa-miR-16|IGF2R	0.2890356	0.01953584	0.137
hsa-miR-16|IPO4	-0.2856802	0.04661078	0.158
hsa-miR-16|ITGA2	-0.3701028	0.008857434	0.049
hsa-miR-16|JUN	-0.2983417	0.01578332	0.046
hsa-miR-16|KCNN4	-0.2734562	0.02751779	0.208
hsa-miR-16|KPNA3	0.4950386	0.0002990744	0.265
hsa-miR-16|LAMC1	0.109312	0.3860499	0.082
hsa-miR-16|LYPLA2	-0.3033397	0.03411023	0.034
hsa-miR-16|MCL1	-0.5420518	5.751881e-05	0.005
hsa-miR-16|MLLT1	-0.7650918	1.545014e-10	0.011
hsa-miR-16|MLLT11	0.4240566	0.002393652	0.008
hsa-miR-16|MRPL20	0.281913	0.04970976	0.114
hsa-miR-16|MSH2	0.06050431	0.6796247	0.208
hsa-miR-16|NOTCH2	0.1121927	0.3735788	0.272
hsa-miR-16|PDCD4	0.08578305	0.557834	0.161
hsa-miR-16|PHLDB2	-0.1169422	0.4235868	0.05
hsa-miR-16|PLK1	0.2331566	0.1069029	0.266
hsa-miR-16|PNN	0.3452566	0.004851802	0.183
hsa-miR-16|PPIF	0.2134294	0.0878099	0.1
hsa-miR-16|PRIM1	0.1719043	0.2375699	0.198
hsa-miR-16|PSAT1	0.3816306	0.006816172	0.2
hsa-miR-16|PTGS2	0.01484186	0.9103825	0.281
hsa-miR-16|RAB21	0.3086343	0.03095522	0.672
hsa-miR-16|RAD51C	0.2455341	0.08903508	0.635
hsa-miR-16|RARS	-0.07011123	0.6321529	0.001
hsa-miR-16|RECK	0.02103964	0.8859022	0.037
hsa-miR-16|RTN4	0.4062695	0.003773024	0.065
hsa-miR-16|SEC24A	0.1586915	0.2067312	0.216
hsa-miR-16|SERPINE2	0.3123041	0.02891384	0.143
hsa-miR-16|SHOC2	0.1443432	0.3224123	0.276
hsa-miR-16|SLC16A3	-0.1408233	0.2631782	0.17
hsa-miR-16|SLC35A1	-0.09132086	0.5325949	0.219
hsa-miR-16|SLC35B3	-0.2456611	0.08886468	0.013
hsa-miR-16|SLC38A1	-0.2856855	0.02106128	0.001
hsa-miR-16|SLC7A1	-0.4619961	0.0001070391	0.001
hsa-miR-16|SNX15	0.1890288	0.1933247	0.241
hsa-miR-16|SPTLC1	-0.1049511	0.4729559	0.26
hsa-miR-16|SQSTM1	-0.0767887	0.5432029	0.183
hsa-miR-16|SRPR	-0.5174594	0.0001404029	0.017
hsa-miR-16|SRPRB	-0.1847602	0.2037611	0.022
hsa-miR-16|TIA1	0.522962	0.0001156729	0.031

Table S2.Results obtained for the test on experimentally validated targets

hsa-miR-16|TMEM109	-0.3677277	0.009338103	0.052
hsa-miR-16|TMEM43	0.1707703	0.2407259	0.104
hsa-miR-16|TOMM34	0.2766981	0.05427342	0.457
hsa-miR-16|TPI1	-0.2398914	0.09686794	0.116
hsa-miR-16|TPM3	-0.1347407	0.3559785	0.223
hsa-miR-16|TXN2	0.3136902	0.02817257	0.483
hsa-miR-16|UBE2S	0.2462861	0.0880296	0.374
hsa-miR-16|UBE2V1	-0.1799604	0.215963	0.058
hsa-miR-16|UBE4A	0.05150746	0.7252298	0.143
hsa-miR-16|UGDH	-0.2915344	0.01846029	0.003
hsa-miR-16|UGP2	-0.05915358	0.6864043	0.31
hsa-miR-16|VTI1B	-0.2553805	0.07655886	0.157
hsa-miR-16|YIF1B	-0.3395605	0.01698828	0.054
hsa-miR-16|ZNF622	-0.4749991	0.000563343	0.134
hsa-miR-17-3p|VIM	-0.2877132	0.04500495	0.269
hsa-miR-17-5p|BMPR2	0.1022628	0.4175956	0.186
hsa-miR-17-5p|CCND1	-0.7916036	1.273634e-11	0.001
hsa-miR-17-5p|PTPRO	-0.2193438	0.0791668	0.188
hsa-miR-181a|BCL2	0.2142582	0.08655574	0.295
hsa-miR-181a|BDNF	0.1415086	0.2608386	0.038
hsa-miR-181a|GATA6	0.2069207	0.09816192	0.026
hsa-miR-181a|HOXA11	-0.1338781	0.3590947	0.027
hsa-miR-181a|NLK	-0.6343105	9.89604e-07	0.005
hsa-miR-181a|PLAG1	0.02216077	0.8609063	0.024
hsa-miR-181b|BCL2	0.2270442	0.06894131	0.112
hsa-miR-181b|GATA6	0.4178924	0.0005327353	0.016
hsa-miR-181b|GRIA2	0.4442284	0.001385981	0.018
hsa-miR-181b|NLK	-0.5451199	5.120555e-05	0.038
hsa-miR-181b|PLAG1	-0.0253037	0.8414191	0.043
hsa-miR-181b|TIMP3	-0.2430529	0.05106939	0.086
hsa-miR-181b|VSNL1	0.2474067	0.04692676	0.008
hsa-miR-181c|BCL2	0.3697074	1.309202e-07	0.02
hsa-miR-181c|GATA6	0.1601449	0.02649464	0.048
hsa-miR-181c|KRAS	-0.6930753	3.410467e-08	0.001
hsa-miR-181c|NLK	-0.5572725	3.194439e-05	0.004
hsa-miR-181c|NOTCH2	-0.118053	0.2035926	0.073
hsa-miR-181c|NOTCH4	0.109331	0.385967	0.246
hsa-miR-182|ADCY6	-0.4068969	0.002166153	0.118
hsa-miR-182|CDKN1A	-0.6432831	6.19783e-07	0.003
hsa-miR-182|EP300	-0.05754756	0.4278637	0.153
hsa-miR-182|FOXO1	0.4274447	0.002188782	0.011
hsa-miR-182|IGF1R	-0.03091992	0.806839	0.108
hsa-miR-182|MITF	-0.04835116	0.5054187	0.179
hsa-miR-182|RARG	0.02221907	0.8795521	0.174
hsa-miR-183|BTRC	0.2225151	0.001921455	0.135
hsa-miR-183|FOXO1	0.4412576	1.507293e-09	0.002
hsa-miR-183|VIL2	-0.09235567	0.202633	0.006
hsa-miR-184|AKT2	0.08631515	0.2338771	0.038
hsa-miR-185|CCNE1	-0.1885458	0.1944863	0.07
hsa-miR-185|CORO2B	0.1461462	0.3163405	0.01
hsa-miR-185|HMGA2	-0.367108	0.00946715	0.028
hsa-miR-186|FOXO1	0.3079957	0.0313224	0.144
hsa-miR-18a|CTGF	-0.1442449	0.3227452	0.022

Table S2.Results obtained for the test on experimentally validated targets

hsa-miR-18a|ESR1	-0.6049967	1.491125e-20	0.001
hsa-miR-18a*|KRAS	0.6736914	1.126317e-07	0.181
hsa-miR-18a|PTEN	-0.320267	5.936783e-06	0.002
hsa-miR-18a|THBS1	-0.2336803	0.001105869	0.054
hsa-miR-18b|ESR1	-0.1639119	0.101445	0.106
hsa-miR-192|CDKN1A	-0.3188589	0.0255466	0.141
hsa-miR-192|DHFR	0.210777	0.1460187	0.246
hsa-miR-193b|CCND1	0.6521497	3.844518e-07	0.081
hsa-miR-193b|MCL1	0.4053876	0.0038567	0.041
hsa-miR-195|BCL2L11	-0.08353807	0.2493237	0.003
hsa-miR-195|CCND1	0.4163453	0.0005614115	0.157
hsa-miR-195|CDK6	-0.2553006	0.0003517622	0.023
hsa-miR-195|E2F3	-0.4168126	1.812742e-09	0.036
hsa-miR-195|MECP2	0.3279221	0.007660723	0.199
hsa-miR-195|WEE1	-0.1218181	0.3337045	0.062
hsa-miR-196a|ANXA1	0.4050955	0.003884777	0.231
hsa-miR-196a|BMP4	0.2508021	0.05876419	0.1
hsa-miR-196a|HOXA7	0.5008894	0.0002467503	0.017
hsa-miR-196a|HOXB8	0.2100843	0.1473791	0.016
hsa-miR-196a|HOXD8	0.4090203	0.003522196	0.054
hsa-miR-196a|KRT5	0.01767231	0.8607594	0.168
hsa-miR-196a|S100A9	0.09467472	0.346324	0.2
hsa-miR-196b|HOXB8	0.09489672	0.5166004	0.003
hsa-miR-197|ACVR1	0.4668491	0.0007209425	0.139
hsa-miR-197|TSPAN3	0.4792992	0.0004933696	0.305
hsa-miR-199a|ETS2	0.4640636	1.206368e-11	0.136
hsa-miR-199a*|ETS2	0.4459366	9.044032e-11	0.012
hsa-miR-199a|JUNB	0.5292794	3.108624e-15	0.106
hsa-miR-199a*|JUNB	0.5224726	7.549517e-15	0.311
hsa-miR-199a|LIF	0.3641415	0.01010648	0.48
hsa-miR-199a|MECP2	0.3267275	0.007898407	0.558
hsa-miR-199a*|MECP2	0.2365961	0.05775885	0.44
hsa-miR-199a*|MET	-0.3262122	3.882811e-06	0.001
hsa-miR-199a*|SMAD1	-0.6898517	4.186608e-08	0.014
hsa-miR-199b-5p|HES1	-0.1002708	0.3184331	0.187
hsa-miR-199b-5p|LAMC2	-0.03809603	0.7052581	0.081
hsa-miR-199b|LAMC2	0.3196532	0.02516186	0.072
hsa-miR-199b|SET	-0.05798355	0.424366	0.064
hsa-miR-19a|ATXN1	-0.1414372	0.2610816	0.073
hsa-miR-19a|CCND1	-0.59669	6.042548e-06	0.004
hsa-miR-19a|CTGF	-0.04928396	0.6966346	0.231
hsa-miR-19a|ERBB4	-0.1674716	0.1823873	0.045
hsa-miR-19a|HOXA5	0.1994113	0.1695392	0.094
hsa-miR-19a|MECP2	-0.07508941	0.5521885	0.104
hsa-miR-19a|NR4A2	0.114753	0.362702	0.18
hsa-miR-19a|PTEN	-0.1832248	0.1440311	0.044
hsa-miR-19a|SOCS1	0.4689715	0.0006764836	0.168
hsa-miR-19a|THBS1	0.2164954	0.1351419	0.084
hsa-miR-19b|BACE1	-0.6083468	3.536246e-06	0.053
hsa-miR-19b|CTGF	-0.1419958	0.3304272	0.051
hsa-miR-19b|NR3C2	0.2009965	0.1083894	0.259
hsa-miR-19b|PTEN	-0.05281908	0.676042	0.07
hsa-miR-19b|SOCS1	0.4168482	0.002886873	0.093

Table S2.Results obtained for the test on experimentally validated targets

hsa-miR-1|ABHD11	0.0004149727	0.996714	0.223
hsa-miR-1|ACPL2	-0.2236025	0.001822895	0.012
hsa-miR-1|ADAR	-0.4063773	8.426272e-05	0.001
hsa-miR-1|ADPGK	-0.0742451	0.3060877	0.078
hsa-miR-1|AGMAT	0.2171797	0.002479118	0.319
hsa-miR-1|AGRN	-0.1565291	0.03014724	0.087
hsa-miR-1|ANKIB1	-0.004260144	0.9768237	0.099
hsa-miR-1|ANP32B	-0.1858702	0.009845526	0.052
hsa-miR-1|ANPEP	-0.2924539	0.04143508	0.167
hsa-miR-1|ANXA2	0.2965701	0.0385269	0.019
hsa-miR-1|AP3B1	0.5158682	1.520011e-05	0.001
hsa-miR-1|AP3D1	-0.1704763	0.01807416	0.008
hsa-miR-1|ARCN1	0.4424394	0.001456767	0.019
hsa-miR-1|ARF3	-0.1671343	0.02049938	0.154
hsa-miR-1|ARF4	0.3793174	0.007189338	0.013
hsa-miR-1|ARHGEF18	-0.2024455	0.00486153	0.123
hsa-miR-1|ARID1A	0.05498905	0.5849506	0.269
hsa-miR-1|ARID2	-0.1864647	0.009608443	0.043
hsa-miR-1|ASH2L	-0.1802443	0.2152274	0.006
hsa-miR-1|ATP6V0A1	-0.2003955	0.005320568	0.012
hsa-miR-1|ATP6V1B2	-0.01720207	0.9066108	0.408
hsa-miR-1|AXL	0.44855	6.813683e-11	0.033
hsa-miR-1|BCKDHB	0.2023232	0.004887895	0.298
hsa-miR-1|BCL2	0.4444575	1.060489e-10	0.222
hsa-miR-1|BDNF	0.6026488	4.607104e-06	0.033
hsa-miR-1|BLCAP	0.06366843	0.3802991	0.016
hsa-miR-1|BRI3BP	-0.3390895	1.500349e-06	0.068
hsa-miR-1|BRWD2	-0.1398474	0.05303368	0.001
hsa-miR-1|CAND1	-0.1855007	0.009995489	0.044
hsa-miR-1|CAP1	0.5021872	0.0002363351	0.011
hsa-miR-1|CDCP1	-0.04620234	0.7525827	0.25
hsa-miR-1|CDK9	0.1463627	0.04279144	0.115
hsa-miR-1|CEBPA	-0.2927599	3.776235e-05	0.007
hsa-miR-1|CHST11	-0.153504	0.1103169	0.174
hsa-miR-1|CHSY1	0.4645657	1.138956e-11	0.118
hsa-miR-1|CLCN3	-0.1505475	0.03713111	0.073
hsa-miR-1|CNN3	-0.0638043	0.5261525	0.078
hsa-miR-1|COIL	-0.3439714	1.034438e-06	0.001
hsa-miR-1|CORO1C	0.3958874	0.004866854	0.007
hsa-miR-1|CPOX	-0.1482212	0.04019412	0.114
hsa-miR-1|CSRP1	0.2963168	3.003727e-05	0.145
hsa-miR-1|CTSC	0.2545105	0.07760237	0.093
hsa-miR-1|DDX5	-0.1454804	0.318574	0.146
hsa-miR-1|DHX15	0.08545204	0.3072278	0.195
hsa-miR-1|DNAJB1	0.07259346	0.6201125	0.139
hsa-miR-1|EGFR	-0.01242735	0.8641604	0.032
hsa-miR-1|EHMT1	-0.1699164	0.01846219	0.058
hsa-miR-1|EHMT2	-0.3574644	3.580315e-07	0.013
hsa-miR-1|EML4	-0.3250272	4.228762e-06	0.001
hsa-miR-1|EPB41L4B	0.1645451	0.02256722	0.086
hsa-miR-1|F2	0.1741686	0.0156883	0.39
hsa-miR-1|FBLN2	-0.3157049	0.02712344	0.02
hsa-miR-1|FN1	0.3654889	0.009811622	0.034

Table S2.Results obtained for the test on experimentally validated targets

hsa-miR-1|FOXP1	0.2121226	0.01822052	0.004
hsa-miR-1|G6PD	-0.1940106	0.181632	0.183
hsa-miR-1|GAK	-0.2258217	0.1187188	0.132
hsa-miR-1|GATA4	-0.03377627	0.6418639	0.38
hsa-miR-1|GCH1	0.3619825	2.481994e-07	0.351
hsa-miR-1|GJA1	0.3623395	0.0105127	0.114
hsa-miR-1|GNPDA2	0.2165039	0.1351262	0.211
hsa-miR-1|GNPNAT1	0.1870438	0.009382365	0.257
hsa-miR-1|GOLGA7	0.1381459	0.0560203	0.155
hsa-miR-1|H3F3B	-0.2958255	0.03904019	0.018
hsa-miR-1|HAND2	0.4779138	2.384981e-12	0.636
hsa-miR-1|HDAC4	-0.3285245	0.0002534439	0.007
hsa-miR-1|HIST1H3B	-0.06212926	0.5370922	0.006
hsa-miR-1|HIST1H3I	0.02523437	0.8022119	0.079
hsa-miR-1|IGF1	0.1937766	0.00970258	0.042
hsa-miR-1|IHPK2	-0.1501209	0.03767751	0.082
hsa-miR-1|IQGAP3	-0.06868992	0.6390902	0.127
hsa-miR-1|ITGB4	-0.09066694	0.3672117	0.207
hsa-miR-1|KCNJ2	0.1916577	0.1870895	0.034
hsa-miR-1|KIAA1598	-0.1137318	0.1162468	0.025
hsa-miR-1|KIAA1618	-0.2348701	0.001041031	0.126
hsa-miR-1|LARP4	0.3569376	0.01181578	0.035
hsa-miR-1|LASP1	0.4093163	1.571463e-06	0.024
hsa-miR-1|LASS2	0.168957	0.02945875	0.038
hsa-miR-1|LIN7C	0.2190358	0.002270325	0.294
hsa-miR-1|LRP1	0.1308517	0.3701578	0.101
hsa-miR-1|LRRC8A	-0.02899908	0.8432057	0.204
hsa-miR-1|LZTFL1	0.0995818	0.3217867	0.004
hsa-miR-1|MEF2A	0.1318589	0.06828394	0.474
hsa-miR-1|MET	-0.1402093	0.05241572	0.01
hsa-miR-1|MMD	-0.1582729	0.1376308	0.001
hsa-miR-1|MOV10	-0.4195145	1.388973e-09	0.001
hsa-miR-1|MRC2	0.2463478	0.0005722368	0.033
hsa-miR-1|MTHFD2	0.4518535	0.0002680659	0.133
hsa-miR-1|MTX1	-0.3248108	4.295039e-06	0.053
hsa-miR-1|MXD4	-0.2569913	0.0746561	0.073
hsa-miR-1|NETO2	-0.2306635	0.001614768	0.133
hsa-miR-1|NOTCH2	-0.03337374	0.6458404	0.143
hsa-miR-1|NP	0.142479	0.04867528	0.062
hsa-miR-1|NRP1	0.2635437	0.0002212465	0.156
hsa-miR-1|OAT	0.338692	0.0172907	0.085
hsa-miR-1|PDCD4	0.2208581	0.03058893	0.089
hsa-miR-1|PDLIM7	0.3043177	0.03350821	0.094
hsa-miR-1|PFTK1	-0.1856446	0.009936855	0.127
hsa-miR-1|PGM2	0.119987	0.09737261	0.026
hsa-miR-1|PICALM	0.5322116	8.28873e-05	0.053
hsa-miR-1|PIM1	0.2196723	0.002202491	0.031
hsa-miR-1|PLEKHB2	-0.2079522	0.1516251	0.004
hsa-miR-1|PLEKHC1	0.1970002	0.006166731	0.073
hsa-miR-1|POGK	-0.2016364	0.005038286	0.104
hsa-miR-1|POLA2	0.0529955	0.7176165	0.413
hsa-miR-1|POLR2K	-0.2876528	5.217877e-05	0.025
hsa-miR-1|POM121	-0.09190452	0.5299677	0.115

Table S2.Results obtained for the test on experimentally validated targets

hsa-miR-1|PPIB	-0.1829946	0.01106689	0.051
hsa-miR-1|PREX1	-0.343885	0.01554846	0.151
hsa-miR-1|PTBP1	-0.241233	0.0007497336	0.081
hsa-miR-1|PTBP2	-0.2613487	0.0002506922	0.009
hsa-miR-1|PTMA	-0.06028783	0.593985	0.162
hsa-miR-1|PTPLAD1	0.09114402	0.3121887	0.078
hsa-miR-1|PTPLB	0.5595577	2.917169e-05	0.007
hsa-miR-1|PTPRF	-0.423132	9.688395e-10	0.002
hsa-miR-1|PWP1	-0.3025813	1.992436e-05	0.063
hsa-miR-1|RAB11FIP2	0.2034709	0.1608419	0.186
hsa-miR-1|RABGAP1L	0.09151269	0.2068022	0.101
hsa-miR-1|RFT1	-0.2562811	0.0003331499	0.005
hsa-miR-1|RHEB	-0.2495968	0.0004805786	0.007
hsa-miR-1|RNF138	0.05367044	0.4596906	0.238
hsa-miR-1|SAC3D1	-0.1714985	0.238696	0.042
hsa-miR-1|SDC4	0.4368412	6.655093e-05	0.146
hsa-miR-1|SERP1	0.4797235	0.0004869091	0.228
hsa-miR-1|SFRS9	-0.338762	1.53789e-06	0.001
hsa-miR-1|SFXN1	0.1624973	0.02432772	0.116
hsa-miR-1|SH2D4A	0.09021128	0.3696344	0.367
hsa-miR-1|SH3BGRL3	0.1100678	0.1708943	0.03
hsa-miR-1|SLC16A9	-0.1608232	0.04560135	0.156
hsa-miR-1|SLC25A1	-0.2165463	0.01803146	0.002
hsa-miR-1|SLC25A30	-0.02914006	0.6882544	0.124
hsa-miR-1|SNX6	0.2225554	0.1242887	0.044
hsa-miR-1|SSNA1	-0.01990105	0.8920391	0.324
hsa-miR-1|SYNE1	0.209288	0.01185639	0.059
hsa-miR-1|TAGLN2	0.2597428	0.07149245	0.093
hsa-miR-1|TDP1	0.2592471	0.07205444	0.273
hsa-miR-1|TH1L	-0.3253208	4.140424e-06	0.001
hsa-miR-1|THBS1	0.5161098	7.586909e-11	0.004
hsa-miR-1|TIMP3	0.4631816	1.33431e-11	0.321
hsa-miR-1|TMSB4X	0.2020266	0.163898	0.201
hsa-miR-1|TPM1	0.4252216	0.002321364	0.027
hsa-miR-1|TPM2	0.5526779	7.694049e-07	0.036
hsa-miR-1|TPM3	-0.224363	0.001756723	0.029
hsa-miR-1|TPM4	0.2807124	8.018072e-05	0.157
hsa-miR-1|TRAPPC3	0.349393	0.01386614	0.009
hsa-miR-1|TRIM2	-0.04422394	0.7628627	0.077
hsa-miR-1|UHRF1	-0.231543	0.01981836	0.068
hsa-miR-1|UST	0.2639724	0.06683772	0.207
hsa-miR-1|WDFY1	0.4161478	0.002939275	0.016
hsa-miR-1|YWHAQ	-0.296123	3.041644e-05	0.019
hsa-miR-200a|BAP1	-0.4369672	2.347399e-10	0.003
hsa-miR-200a|CTNNB1	0.3028042	0.0101809	0.089
hsa-miR-200a|CTNNBIP1	-0.0875912	0.2270099	0.007
hsa-miR-200a|DLX5	-0.01982825	0.8924317	0.155
hsa-miR-200a|ELMO2	-0.02533727	0.7272042	0.156
hsa-miR-200a|ERBB2IP	-0.303993	0.002494101	0.003
hsa-miR-200a|G3BP2	0.348771	0.001729019	0.161
hsa-miR-200a|RAB30	0.1795727	0.01269268	0.584
hsa-miR-200a|ZEB1	-0.2262021	0.1180826	0.025
hsa-miR-200a|ZEB2	-0.4753922	1.221689e-11	0.021

Table S2.Results obtained for the test on experimentally validated targets

hsa-miR-200b|BAP1	-0.4782304	2.296251e-12	0.001
hsa-miR-200b|ELMO2	-0.04651004	0.5217823	0.04
hsa-miR-200b|EP300	-0.09892783	0.1721952	0.009
hsa-miR-200b|ERBB2IP	-0.3221592	0.000869146	0.001
hsa-miR-200b|ERRFI1	0.1596401	0.02698068	0.001
hsa-miR-200b|PTPN12	0.1054792	0.1453693	0.127
hsa-miR-200b|RERE	0.1416182	0.3317279	0.067
hsa-miR-200b|ZEB1	-0.233829	0.1058665	0.041
hsa-miR-200b|ZEB2	-0.4821091	2.607736e-11	0.018
hsa-miR-200c|BMI1	-0.6689967	1.484469e-07	0.001
hsa-miR-200c|CDH1	0.9052645	4.867469e-09	0.022
hsa-miR-200c|ERBB2IP	-0.5299189	9.010697e-05	0.003
hsa-miR-200c|ERRFI1	-0.04761796	0.7452528	0.186
hsa-miR-200c|KLHL20	-0.1608318	0.2005951	0.122
hsa-miR-200c|PTPRD	-0.02379924	0.8507361	0.136
hsa-miR-200c|TUBB3	-0.1269799	0.3134696	0.204
hsa-miR-200c|ZEB1	-0.3579593	0.01155921	0.048
hsa-miR-200c|ZEB2	-0.518053	0.0001375208	0.012
hsa-miR-200c|ZFHX1B	-0.03928278	0.6965191	0.057
hsa-miR-203|SOCS3	0.3115206	1.09095e-05	0.274
hsa-miR-204|ARPC1B	-0.6957815	2.865266e-08	0.004
hsa-miR-204|AURKB	0.1898666	0.1913219	0.128
hsa-miR-204|BCL2	-0.1293899	0.0736635	0.044
hsa-miR-204|BMP1	-0.4292749	0.002084713	0.066
hsa-miR-204|CDC25B	-0.3946376	1.477695e-08	0.001
hsa-miR-204|CDH11	-0.4130245	0.00318331	0.042
hsa-miR-204|CTSC	-0.564193	2.421451e-05	0.009
hsa-miR-204|EFNB1	0.06138441	0.5419927	0.112
hsa-miR-204|FBN2	-0.1202326	0.410569	0.174
hsa-miR-204|HMGA2	-0.2288309	0.1137567	0.246
hsa-miR-204|HOXA10	-0.3250532	0.02267258	0.062
hsa-miR-204|HOXB7	-0.3403621	0.01671317	0.112
hsa-miR-204|ITGB4	-0.07368781	0.463966	0.168
hsa-miR-204|MCL1	-0.2341792	0.02122147	0.082
hsa-miR-204|MEIS1	0.0827154	0.4108818	0.004
hsa-miR-204|MEIS2	0.5676273	2.105525e-05	0.008
hsa-miR-204|MMP3	-0.2609561	0.07013168	0.125
hsa-miR-204|MMP9	0.13919	0.3401691	0.106
hsa-miR-204|SHC1	-0.7519884	4.719252e-10	0.001
hsa-miR-204|SNAI2	-0.5486866	4.466804e-05	0.003
hsa-miR-204|SOX4	0.7585761	2.715734e-10	0.007
hsa-miR-204|SPARC	-0.2646404	0.0002077731	0.011
hsa-miR-204|TGFBR2	0.1126658	0.1197301	0.243
hsa-miR-205|DDX5	-0.3224622	0.01443162	0.003
hsa-miR-205|ERBB3	0.7563462	3.280687e-10	0.014
hsa-miR-205|INPPL1	-0.3448983	0.01522653	0.074
hsa-miR-205|LRP1	-0.2905397	0.04284755	0.149
hsa-miR-205|MED1	0.561987	2.646808e-05	0.091
hsa-miR-205|SIP1	0.00486114	0.9713717	0.134
hsa-miR-205|VEGF	0.0318737	0.7516838	0.04
hsa-miR-205|VEGFA	0.4337939	0.0007487833	0.149
hsa-miR-205|ZEB1	-0.32447	0.02293101	0.077
hsa-miR-205|ZEB2	-0.5421268	5.735634e-05	0.008

Table S2.Results obtained for the test on experimentally validated targets

hsa-miR-206|ESR1	-0.2560538	0.05454041	0.132
hsa-miR-206|FSTL1	-0.1075823	0.4257128	0.199
hsa-miR-206|GJA1	0.06142554	0.6498955	0.416
hsa-miR-206|NOTCH3	-0.3003711	0.02319468	0.048
hsa-miR-206|UTRN	-0.005269598	0.9689674	0.181
hsa-miR-20a|APP	0.4247232	0.002352048	0.185
hsa-miR-20a|BCL2	0.1914648	0.1265471	0.094
hsa-miR-20a|BMPR2	-0.0261956	0.8359057	0.15
hsa-miR-20a|CCND1	-0.8047805	3.209752e-12	0.001
hsa-miR-20a|CDKN1A	-0.4864824	0.0003937801	0.062
hsa-miR-20a|HIF1A	-0.1545565	0.2889837	0.127
hsa-miR-20a|IL8	0.1787051	0.1543581	0.093
hsa-miR-20a|JAK1	-0.2226467	0.07464243	0.085
hsa-miR-20a|MEF2D	-0.37748	0.007498267	0.01
hsa-miR-20a|PTEN	0.03050368	0.8093902	0.174
hsa-miR-20a|TGFBR2	-0.204408	0.1024039	0.083
hsa-miR-20b|CDKN1A	-0.8066458	2.61869e-12	0.001
hsa-miR-20b|HIF1A	-0.372737	0.008349502	0.082
hsa-miR-20b|HIPK3	-0.0698035	0.580586	0.276
hsa-miR-20b|MYLIP	0.02269228	0.8576046	0.42
hsa-miR-20b|STAT3	-0.3678682	0.009309058	0.077
hsa-miR-210|ACVR1B	0.4543772	3.592415e-11	0.001
hsa-miR-210|ATP11C	0.04683139	0.5189068	0.09
hsa-miR-210|BDNF	0.1561419	0.2142124	0.101
hsa-miR-210|CBX1	0.3208663	5.690327e-06	0.001
hsa-miR-210|CDK10	-0.06216177	0.3916975	0.023
hsa-miR-210|CHD9	0.09300243	0.3784968	0.01
hsa-miR-210|CLASP2	0.2290825	0.01743747	0.032
hsa-miR-210|CPEB2	0.005564651	0.9389392	0.028
hsa-miR-210|DDAH1	0.2250876	0.001695726	0.076
hsa-miR-210|E2F3	0.3365777	1.812229e-06	0.004
hsa-miR-210|EFNA3	0.2238969	0.07298463	0.122
hsa-miR-210|ELK3	-0.3787278	6.071684e-08	0.026
hsa-miR-210|FAM116A	0.1454856	0.04406523	0.001
hsa-miR-210|GPD1L	0.04980392	0.6935916	0.124
hsa-miR-210|HECTD1	-0.2370839	0.0009295891	0.034
hsa-miR-210|HOXA1	-0.05572147	0.6593094	0.303
hsa-miR-210|HOXA3	0.05179412	0.4755531	0.017
hsa-miR-210|HOXA9	0.1141944	0.3650583	0.067
hsa-miR-210|ISCU	-0.1881718	0.008955458	0.001
hsa-miR-210|KIAA1161	0.08840526	0.2227042	0.187
hsa-miR-210|MDGA1	0.02325458	0.7488439	0.121
hsa-miR-210|MIB1	0.207912	0.003806205	0.019
hsa-miR-210|MID1IP1	0.1869553	0.009416601	0.116
hsa-miR-210|MNT	0.009570077	0.8951868	0.067
hsa-miR-210|NCAM1	-0.2335169	0.001115054	0.008
hsa-miR-210|NIPBL	-0.2272077	0.006737404	0.021
hsa-miR-210|NPTX1	-0.04304536	0.733505	0.135
hsa-miR-210|P4HB	0.414028	2.379308e-09	0.054
hsa-miR-210|PIM1	-0.09432053	0.2938245	0.063
hsa-miR-210|PTAR1	0.09855822	0.1738114	0.142
hsa-miR-210|PTPN1	0.04354292	0.5487108	0.02
hsa-miR-210|RAD52	-0.01562517	0.829683	0.088

Table S2.Results obtained for the test on experimentally validated targets

hsa-miR-210|SEH1L	0.1018435	0.1598347	0.085
hsa-miR-210|SERTAD2	0.207306	0.003912047	0.045
hsa-miR-210|SMCHD1	-0.4601079	0.0001151765	0.025
hsa-miR-210|TNPO1	0.2235827	0.001824646	0.004
hsa-miR-210|UBQLN1	0.1502034	0.0375713	0.038
hsa-miR-210|XIST	-0.05875784	0.5594468	0.053
hsa-miR-214|EZH2	-0.5143276	2.334448e-14	0.001
hsa-miR-214|PTEN	0.363742	2.148661e-07	0.08
hsa-miR-215|CDKN1A	-0.02536348	0.7269332	0.025
hsa-miR-215|DHFR	0.1745262	0.01547255	0.323
hsa-miR-215|DTL	-0.1612789	0.02543025	0.017
hsa-miR-217|IRS1	0.06582976	0.3643052	0.209
hsa-miR-217|NR4A2	-0.08553076	0.2381702	0.092
hsa-miR-217|PTEN	-0.1558276	0.03090354	0.012
hsa-miR-217|ROBO1	-0.0974758	0.4255552	0.028
hsa-miR-217|SIRT1	-0.251029	0.08189079	0.278
hsa-miR-218|COL1A1	0.4989299	1.767475e-13	0.022
hsa-miR-218|EBP	-0.2248502	0.01003739	0.025
hsa-miR-218|EFNA1	0.2482575	0.01219976	0.058
hsa-miR-218|LAMB3	-0.3460493	0.01368899	0.056
hsa-miR-218|LASP1	0.3701105	0.008855925	0.193
hsa-miR-218|MAFG	-0.01213756	0.9340324	0.363
hsa-miR-218|MBNL2	0.357065	3.697181e-07	0.001
hsa-miR-218|MRPS27	-0.3031663	0.0003875869	0.079
hsa-miR-218|NFE2L1	-0.1897292	0.008394367	0.041
hsa-miR-218|NUP93	-0.1926007	0.007441062	0.079
hsa-miR-218|SP1	-0.3542801	0.01250612	0.084
hsa-miR-219|CAMK2G	0.4222622	0.002508883	0.295
hsa-miR-21|ACTA2	0.1368721	0.05834583	0.069
hsa-miR-21|APAF1	0.2871315	0.04545975	0.886
hsa-miR-21|BASP1	0.2804058	0.02367046	0.009
hsa-miR-21|BCL2	0.1540598	0.2204621	0.188
hsa-miR-21|BMPR2	-0.1891674	0.04191554	0.036
hsa-miR-21|BTG2	0.1345358	0.3567173	0.157
hsa-miR-21|CDC25A	0.1728786	0.1684685	0.376
hsa-miR-21|CDK6	0.07925694	0.274497	0.063
hsa-miR-21|CDKN1A	-0.140864	0.3343355	0.122
hsa-miR-21|CFL2	-0.4004545	8.648986e-09	0.001
hsa-miR-21|DAXX	0.3308238	2.77592e-06	0.09
hsa-miR-21|DERL1	0.1372157	0.05771081	0.029
hsa-miR-21|E2F2	-0.08043069	0.5827503	0.309
hsa-miR-21|FAM3C	-0.156738	0.02992512	0.118
hsa-miR-21|FAS	-0.3988874	0.0009972922	0.004
hsa-miR-21|FMOD	0.1557831	0.1576351	0.01
hsa-miR-21|GLCCI1	0.03775951	0.6030775	0.044
hsa-miR-21|HIPK3	-0.3401621	0.005563445	0.075
hsa-miR-21|HNRNPK	0.1817436	0.2113719	0.531
hsa-miR-21|HNRPK	-0.1552335	0.03155657	0.05
hsa-miR-21|IL6R	-0.04389285	0.5454999	0.039
hsa-miR-21|JAG1	0.3342452	2.156433e-06	0.046
hsa-miR-21|LRRFIP1	-0.2726171	0.0001304844	0.028
hsa-miR-21|MARCKS	0.2856323	5.919934e-05	0.078
hsa-miR-21|MTAP	-0.1412032	0.2618793	0.273

Table S2.Results obtained for the test on experimentally validated targets

hsa-miR-21|MYC	-0.1863468	0.1372056	0.043
hsa-miR-21|NCAPG	0.3766006	7.293208e-08	0.119
hsa-miR-21|NFIB	-0.1998023	0.05854097	0.03
hsa-miR-21|PDCD4	0.1837764	0.2062217	0.036
hsa-miR-21|PLOD3	0.3177079	7.107684e-06	0.013
hsa-miR-21|PPIF	-0.2861295	5.73938e-05	0.003
hsa-miR-21|PTEN	-0.2338963	0.001093825	0.027
hsa-miR-21|RASA1	0.02848026	0.8459766	0.285
hsa-miR-21|RASGRP1	-0.3156621	0.01042446	0.158
hsa-miR-21|RECK	-0.1484446	0.039891	0.134
hsa-miR-21|REST	-0.2323615	0.02179049	0.056
hsa-miR-21|RHOB	-0.2792877	8.745146e-05	0.002
hsa-miR-21|RPS7	0.3207146	5.751791e-06	0.089
hsa-miR-21|RTN4	-0.1773472	0.01385939	0.006
hsa-miR-21|SESN1	0.1944074	0.1207108	0.104
hsa-miR-21|SGK3	0.2587325	0.07264158	0.063
hsa-miR-21|SLC16A10	-0.2024913	0.004851685	0.001
hsa-miR-21|SOCS5	0.04875366	0.699743	0.252
hsa-miR-21|SPRY1	-0.1318743	0.2135632	0.051
hsa-miR-21|SPRY2	-0.338703	1.544753e-06	0.007
hsa-miR-21|TGFB1	0.1625088	0.1958788	0.357
hsa-miR-21|TGFBI	0.1984184	0.1130892	0.254
hsa-miR-21|TGFBR2	-0.1648252	0.02233516	0.008
hsa-miR-21|TGFBR3	-0.304839	1.714445e-05	0.001
hsa-miR-21|TIMP3	-0.2639213	0.06689251	0.14
hsa-miR-21|TM9SF3	0.2604504	0.07069635	0.192
hsa-miR-21|TOPORS	-0.3280966	3.387516e-06	0.001
hsa-miR-21|TP53BP2	0.2316541	0.001224868	0.199
hsa-miR-21|TPM1	0.286699	5.538911e-05	0.028
hsa-miR-21|WFS1	0.2186082	0.002316951	0.067
hsa-miR-21|WIBG	-0.0911089	0.4169376	0.029
hsa-miR-221|ARIH2	-0.477028	2.6355e-06	0.001
hsa-miR-221|BNIP3L	0.3310785	4.544524e-06	0.001
hsa-miR-221|BRAP	0.2356066	0.05884413	0.302
hsa-miR-221|CDKN1B	-0.3271335	9.300527e-05	0.056
hsa-miR-221|CDKN1C	0.6062254	5.805859e-07	0.176
hsa-miR-221|CREBZF	0.246544	0.0005662748	0.018
hsa-miR-221|DDIT4	-0.3254409	0.02250214	0.117
hsa-miR-221|DKK2	-0.1155748	0.3592521	0.13
hsa-miR-221|FOS	-0.3792853	5.785622e-08	0.001
hsa-miR-221|FOXO3	0.3281346	3.378181e-06	0.105
hsa-miR-221|HMG2L1	0.4136693	2.463694e-09	0.003
hsa-miR-221|HOXB5	-0.3368548	0.006073228	0.023
hsa-miR-221|ICAM1	-0.1662119	0.02121639	0.023
hsa-miR-221|KIT	-0.3499982	0.01369114	0.094
hsa-miR-221|MYBL1	0.2315446	0.001231623	0.151
hsa-miR-221|TBK1	0.4400895	0.001554642	0.099
hsa-miR-221|USP18	-0.1361094	0.1227691	0.308
hsa-miR-222|CDKN1B	-0.2758957	0.0001073239	0.039
hsa-miR-222|CDKN1C	0.5946838	1.0725e-06	0.238
hsa-miR-222|FOS	-0.4083731	4.102206e-09	0.001
hsa-miR-222|FOXO3	0.3051513	1.679014e-05	0.072
hsa-miR-222|KIT	0.03002086	0.8123519	0.189

Table S2.Results obtained for the test on experimentally validated targets

hsa-miR-222|PPP2R2A	-0.4260093	7.252525e-10	0.001
hsa-miR-222|SOD2	0.08195289	0.5064705	0.113
hsa-miR-222|STAT5A	0.2857615	0.03117682	0.258
hsa-miR-223|E2F1	0.1266372	0.2069725	0.27
hsa-miR-223|IRS1	-0.3365583	1.814863e-06	0.009
hsa-miR-223|LMO2	0.2881788	5.0484e-05	0.06
hsa-miR-223|MEF2C	0.1842486	0.01051877	0.026
hsa-miR-223|NFIA	-0.04541626	0.6520045	0.001
hsa-miR-223|RHOB	0.2539878	0.0003781945	0.037
hsa-miR-223|STMN1	0.1434296	0.152454	0.266
hsa-miR-224|AP2M1	0.5816416	1.170653e-05	0.195
hsa-miR-224|API5	-0.5464392	4.869162e-05	0.018
hsa-miR-224|ATF2	0.298084	0.0009052375	0.001
hsa-miR-224|CD40	0.1674433	0.04758399	0.15
hsa-miR-224|CDC42	0.3085788	0.01238682	0.012
hsa-miR-224|CXCR4	-0.522938	0.0001157716	0.015
hsa-miR-224|FOSB	-0.2446156	0.004411841	0.005
hsa-miR-224|JAG1	-0.2354819	0.05898208	0.039
hsa-miR-224|KLK10	0.03189528	0.7515211	0.161
hsa-miR-224|NCOA6	-0.2524354	0.003188189	0.004
hsa-miR-224|NIT1	-0.08822947	0.5139917	0.006
hsa-miR-224|PDGFRB	0.6218863	1.846601e-06	0.052
hsa-miR-22|BMP7	0.09040808	0.4738561	0.085
hsa-miR-22|PTEN	0.07367746	0.5597084	0.066
hsa-miR-23a|CXCL12	0.1733062	0.1674018	0.019
hsa-miR-23a|HES1	-0.4021107	0.000898996	0.008
hsa-miR-23a|POU4F2	-0.1093483	0.3858911	0.344
hsa-miR-23a|RING1	0.4406884	0.001529155	0.422
hsa-miR-23b|MET	0.2683599	0.03066268	0.158
hsa-miR-23b|NOTCH1	0.1730998	0.2342736	0.066
hsa-miR-23b|RB1	0.5665941	2.196332e-05	0.409
hsa-miR-24|AURKB	-0.5457465	4.99971e-05	0.027
hsa-miR-24|BRCA1	0.01412869	0.9110577	0.033
hsa-miR-24|CCNA2	-0.5293296	9.20532e-05	0.032
hsa-miR-24|CDK4	-0.3929704	0.005220292	0.007
hsa-miR-24|DHFR	-0.5051218	0.0002142383	0.026
hsa-miR-24|E2F2	-0.2708819	0.05975613	0.075
hsa-miR-24|FEN1	-0.5236741	0.0001127812	0.023
hsa-miR-24|MAPK14	-0.385981	0.001495639	0.067
hsa-miR-24|MYC	-0.3382969	0.005846101	0.031
hsa-miR-24|NOTCH1	-0.2256538	0.07070485	0.028
hsa-miR-24|TRIB3	0.2357767	0.1029082	0.174
hsa-miR-25|BCL2L11	0.2074431	0.003887868	0.059
hsa-miR-25|KLF4	-0.3969388	1.196998e-08	0.001
hsa-miR-25|PCAF	-0.3108972	1.138475e-05	0.002
hsa-miR-26a|CCND2	0.4771781	0.0005268406	0.001
hsa-miR-26a|CCNE2	-0.1035227	0.4118513	0.041
hsa-miR-26a|CDC6	-0.2295165	0.06589276	0.119
hsa-miR-26a|EZH2	0.294313	0.01732411	0.33
hsa-miR-26a|HMGA1	-0.4644113	0.0007752352	0.067
hsa-miR-26a|HMGA2	-0.4378026	0.001655467	0.025
hsa-miR-26a|LIF	-0.4705751	0.0006445455	0.071
hsa-miR-26a|PLAG1	0.04811524	0.7034918	0.094

Table S2.Results obtained for the test on experimentally validated targets

hsa-miR-26a|PTEN	-0.4299159	0.0003513986	0.05
hsa-miR-26a|RB1	0.4918679	0.0003314499	0.415
hsa-miR-26a|SERBP1	-0.438876	0.001607441	0.02
hsa-miR-26a|SMAD1	0.6046653	4.19784e-06	0.148
hsa-miR-26a|TGFBR2	-0.1384868	0.2712607	0.028
hsa-miR-26b|SERBP1	-0.2202225	0.07794238	0.016
hsa-miR-27a|FOXO1	-0.04257679	0.7714526	0.2
hsa-miR-27a|GCA	-0.3188393	0.0255562	0.162
hsa-miR-27a|MYT1	0.2688642	0.03033858	0.139
hsa-miR-27a|PHB	-0.1827471	0.2088183	0.293
hsa-miR-27a|RUNX1	0.4646662	0.0007693913	0.005
hsa-miR-27a|SP1	-0.3097087	0.03034556	0.042
hsa-miR-27a|SP3	0.004030917	0.9780704	0.319
hsa-miR-27a|ZBTB10	0.1149183	0.3620062	0.021
hsa-miR-27b|CYP1B1	-0.0411866	0.7787236	0.177
hsa-miR-27b|NOTCH1	-0.2901897	0.01903265	0.042
hsa-miR-27b|PPARG	-0.2047431	0.1581848	0.06
hsa-miR-296|HGS	-0.08402946	0.5659419	0.172
hsa-miR-299-5p|CDKN1A	0.2843713	0.0003330722	0.025
hsa-miR-29a|BACE1	0.005688144	0.9690583	0.033
hsa-miR-29a|BCL2	0.2163019	0.08352374	0.143
hsa-miR-29a|CDC42	0.3204151	0.02479735	0.091
hsa-miR-29a|COL3A1	-0.1607375	0.2008626	0.084
hsa-miR-29a|COL4A1	-0.1837902	0.1427765	0.088
hsa-miR-29a|COL4A2	-0.1606311	0.2011647	0.125
hsa-miR-29a|DKK1	0.5957952	6.290765e-06	0.077
hsa-miR-29a|DNMT3B	-0.4204661	0.002629116	0.147
hsa-miR-29a|MCL1	-0.4345014	0.0002986047	0.009
hsa-miR-29a|PIK3R1	0.1554438	0.2162938	0.013
hsa-miR-29a|RAN	-0.2627641	0.0681421	0.259
hsa-miR-29a|SFRP2	-0.4564873	0.0009779933	0.252
hsa-miR-29a|SPARC	0.1707133	0.2408854	0.092
hsa-miR-29b|ACVR2A	-0.1977239	0.005976908	0.051
hsa-miR-29b|BACE1	-0.3227347	0.0003600074	0.022
hsa-miR-29b|BCL2	0.1181263	0.1027139	0.036
hsa-miR-29b|CDC42	0.3521618	0.01308091	0.037
hsa-miR-29b|CDK6	0.04144637	0.56814	0.106
hsa-miR-29b|COL1A1	0.328664	0.02112631	0.06
hsa-miR-29b|COL3A1	-0.1587659	0.2758893	0.092
hsa-miR-29b|COL4A2	-0.2916658	0.04201195	0.119
hsa-miR-29b|COL5A3	-0.5485122	4.49689e-05	0.015
hsa-miR-29b|CTNNBIP1	-0.2875649	0.04512049	0.037
hsa-miR-29b|DNAJB11	0.4028661	0.004105006	0.315
hsa-miR-29b|DNMT1	-0.1220345	0.09175237	0.022
hsa-miR-29b|DNMT3A	-0.194369	0.006903106	0.03
hsa-miR-29b|DNMT3B	-0.26065	0.03598912	0.1
hsa-miR-29b|HDAC4	-0.290535	0.04285107	0.087
hsa-miR-29b|MCL1	-0.3099762	0.01197618	0.001
hsa-miR-29b|PIK3R1	0.2330692	0.002147469	0.035
hsa-miR-29b|SFPQ	0.2230813	0.1233789	0.152
hsa-miR-29b|SP1	-0.249811	0.0834342	0.055
hsa-miR-29b|TGFB3	-0.5495958	4.312872e-05	0.054
hsa-miR-29c|BCL2	0.2114353	0.03379514	0.146

Table S2.Results obtained for the test on experimentally validated targets

hsa-miR-29c|CDC42	0.4322345	0.001925694	0.011
hsa-miR-29c|COL15A1	-0.4295183	0.002071208	0.038
hsa-miR-29c|COL1A1	-0.152656	0.2247469	0.066
hsa-miR-29c|COL1A2	-0.1927993	0.1781917	0.088
hsa-miR-29c|COL3A1	-0.3701212	0.008853811	0.022
hsa-miR-29c|COL4A1	-0.3728387	0.008330404	0.03
hsa-miR-29c|COL4A2	-0.3532737	0.01277645	0.01
hsa-miR-29c|DNMT3B	-0.3528007	0.01290521	0.043
hsa-miR-29c|FBN1	-0.1853234	0.1394157	0.077
hsa-miR-29c|LAMC1	-0.2767546	0.02563082	0.027
hsa-miR-29c|MCL1	-0.3619706	0.003048026	0.014
hsa-miR-29c|PIK3R1	0.2008376	0.1664455	0.084
hsa-miR-29c|SPARC	0.2375257	0.1003062	0.086
hsa-miR-29c|TDG	-0.1720822	0.2370772	0.075
hsa-miR-302a|CCND1	-0.2672673	0.0374732	0.018
hsa-miR-302a|CDK4	0.1658783	0.2546676	0.072
hsa-miR-302a|LEFTY1	0.8063267	2.712053e-12	0.012
hsa-miR-302a|LEFTY2	0.7759577	5.787415e-11	0.005
hsa-miR-302b|CCND2	-0.1746214	0.01541552	0.015
hsa-miR-302d|KLF13	-0.5574549	3.17145e-05	0.002
hsa-miR-302d|MBNL2	-0.3941983	0.0003725192	0.001
hsa-miR-302d|NR4A2	-0.09801668	0.1761996	0.313
hsa-miR-30a*|CYR61	-0.03725584	0.7114685	0.258
hsa-miR-30a|KRT7	0.05347925	0.5953146	0.332
hsa-miR-30a*|THBS1	0.01154148	0.9088015	0.333
hsa-miR-30d|GNAI2	0.1967845	0.1753457	0.281
hsa-miR-31|LATS2	-0.09658818	0.1826159	0.199
hsa-miR-31|PPP2R2A	0.3324205	0.01961299	0.11
hsa-miR-31|RHOA	0.3114412	0.0004426419	0.067
hsa-miR-320|BCL2	0.4230684	0.0004462083	0.011
hsa-miR-320|MCL1	-0.3117714	0.01146599	0.026
hsa-miR-324-5p|SMO	0.2892536	4.718068e-05	0.006
hsa-miR-326|NOTCH1	-0.3264654	0.02205686	0.032
hsa-miR-326|NOTCH2	0.02594718	0.7209068	0.04
hsa-miR-326|SMO	-0.1135905	0.1167037	0.083
hsa-miR-328|ABCG2	0.1483411	0.04003122	0.733
hsa-miR-328|BACE1	0.7317312	2.330946e-09	0.355
hsa-miR-328|CD44	0.335545	0.01842503	0.096
hsa-miR-335|ARPC5L	-0.1421769	0.3298044	0.072
hsa-miR-335|MERTK	0.1756426	0.01481549	0.061
hsa-miR-335|PTPRN2	0.4117967	0.003284025	0.16
hsa-miR-335|RASA1	-0.1440771	0.3233145	0.017
hsa-miR-335|RB1	0.4165065	0.002912338	0.009
hsa-miR-335|SOX4	0.4754582	0.000555468	0.05
hsa-miR-33b|BCL2	0.1669995	0.09508217	0.175
hsa-miR-345|CDKN1A	-0.09253317	0.2017629	0.038
hsa-miR-346|EFEMP2	-0.1454556	0.3186574	0.093
hsa-miR-346|IL18	-0.05377889	0.7136192	0.237
hsa-miR-346|LIF	-0.1105039	0.4497224	0.43
hsa-miR-34a|BCL2	-0.09934163	0.170399	0.012
hsa-miR-34a|BIRC3	0.2389185	0.0008456621	0.091
hsa-miR-34a|CCND1	0.6945342	3.105519e-08	0.109
hsa-miR-34a|CCND3	0.6483929	4.715584e-07	0.135

Table S2.Results obtained for the test on experimentally validated targets

hsa-miR-34a|CCNE2	-0.1856168	0.1387795	0.173
hsa-miR-34a|CDC25A	-0.02475439	0.8448185	0.131
hsa-miR-34a|CDC25C	-0.1624155	0.1961389	0.188
hsa-miR-34a|CDK4	-0.6022348	4.695562e-06	0.001
hsa-miR-34a|CDK6	0.3167885	7.579533e-06	0.021
hsa-miR-34a|DLL1	-0.3648483	0.009950864	0.061
hsa-miR-34a|E2F3	-0.7614087	2.129842e-10	0.012
hsa-miR-34a|E2F5	-0.6993629	2.268693e-08	0.001
hsa-miR-34a|HMGA2	-0.1651462	0.1886227	0.088
hsa-miR-34a|HNF4A	-0.02267606	0.7548908	0.016
hsa-miR-34a|JAG1	-0.1805851	0.1499978	0.163
hsa-miR-34a|MET	0.07408595	0.3071285	0.065
hsa-miR-34a|MYC	0.4455287	0.001336474	0.065
hsa-miR-34a|MYCN	-0.6220314	1.833482e-06	0.002
hsa-miR-34a|NOTCH1	-0.5051819	0.0002138054	0.037
hsa-miR-34a|NOTCH2	-0.1589942	0.2058551	0.054
hsa-miR-34a|SIRT1	-0.4439793	0.001395651	0.01
hsa-miR-34a|VAMP2	-0.1315116	0.06902067	0.002
hsa-miR-34b|BCL2	-0.2735451	0.0001234946	0.015
hsa-miR-34b|HMGA2	0.1862702	0.200025	0.048
hsa-miR-34b|MYC	-0.2695259	0.0001565296	0.004
hsa-miR-34b|NOTCH1	-0.3490926	0.01395371	0.129
hsa-miR-34b|NOTCH2	0.08845895	0.2224222	0.152
hsa-miR-34c|BCL2	0.1549267	0.03189845	0.271
hsa-miR-34c|E2F3	0.4772287	2.588596e-12	0.094
hsa-miR-34c|HMGA2	0.2622848	0.0686651	0.086
hsa-miR-34c|MET	-0.08193922	0.2585309	0.114
hsa-miR-34c|MYC	0.2700193	0.06060578	0.011
hsa-miR-34c|NOTCH1	-0.2765163	0.0544384	0.13
hsa-miR-34c|NOTCH2	0.08995691	0.2146584	0.194
hsa-miR-34c|NOTCH3	0.468383	7.333467e-12	0.018
hsa-miR-363|CDKN1A	-0.764649	1.606299e-10	0.012
hsa-miR-370|HMGA2	-0.2912589	0.04231234	0.059
hsa-miR-370|MAP3K8	-0.0714455	0.5716936	0.414
hsa-miR-372|CDKN1A	-0.5670822	2.152992e-05	0.027
hsa-miR-372|KLF13	-0.3697105	0.008935324	0.061
hsa-miR-372|MBNL2	-0.2620029	0.06897421	0.14
hsa-miR-372|RECK	-0.04784716	0.7440681	0.346
hsa-miR-372|WEE1	0.6088781	3.449189e-06	0.133
hsa-miR-373|ADAM9	-0.5565265	3.290064e-05	0.01
hsa-miR-373|ARHGEF3	-0.3263938	0.02208773	0.012
hsa-miR-373|BAZ1A	-0.4526891	0.001091067	0.017
hsa-miR-373|CD44	-0.5710039	1.832321e-05	0.008
hsa-miR-373|CD83	0.3208197	0.01496726	0.015
hsa-miR-373|CENPF	0.3378385	0.01759232	0.061
hsa-miR-373|CFL2	-0.5099098	0.0001821826	0.023
hsa-miR-373|FYCO1	-0.5563075	3.318633e-05	0.007
hsa-miR-373|GBAS	-0.4986044	0.0002661066	0.013
hsa-miR-373|GBP3	-0.351264	0.01333124	0.025
hsa-miR-373|GLTP	-0.2783792	0.05276674	0.038
hsa-miR-373|GPSM2	-0.3034564	0.03403792	0.136
hsa-miR-373|HERPUD1	-0.4519879	0.001113178	0.024
hsa-miR-373|HSPA14	0.8493778	1.221245e-14	0.008

hsa-miR-373|INSIG2	-0.5537462	3.670108e-05	0.011
hsa-miR-373|KIF23	0.7082419	1.252947e-08	0.019
hsa-miR-373|KLHL12	0.08329419	0.5693577	0.142
hsa-miR-373|LMNB1	0.4093287	0.00349501	0.022
hsa-miR-373|LUC7L2	-0.2213286	0.0980129	0.03
hsa-miR-373|MICA	-0.3163603	0.02678923	0.025
hsa-miR-373|MKRN1	0.1716636	0.206964	0.058
hsa-miR-373|NIN	-0.6365416	8.821398e-07	0.018
hsa-miR-373|NUPL1	0.2845671	0.03192011	0.016
hsa-miR-373|PHC2	-0.5923192	7.347342e-06	0.003
hsa-miR-373|PRC1	-0.1085937	0.4576436	0.135
hsa-miR-373|RAD23B	0.1914115	0.1537716	0.053
hsa-miR-373|RECK	0.3878623	0.0028724	0.101
hsa-miR-373|RELA	-0.6696358	1.430124e-07	0.002
hsa-miR-373|RNF149	-0.296434	0.03862031	0.103
hsa-miR-373|RPIA	-0.2486734	0.06214394	0.092
hsa-miR-373|STK4	-0.2805841	0.05084202	0.13
hsa-miR-373|TMEM14A	-0.3758691	0.007778522	0.024
hsa-miR-373|TNFAIP1	-0.6586611	2.680286e-07	0.03
hsa-miR-373|TTC8	-0.5520491	3.921491e-05	0.015
hsa-miR-373|TUSC2	-0.1112197	0.4101399	0.023
hsa-miR-373|USP12	0.1177748	0.3829232	0.074
hsa-miR-373|ZHX1	-0.735595	1.738153e-09	0.004
hsa-miR-373|ZNF226	-0.5066572	0.0002034403	0.005
hsa-miR-375|ADIPOR2	0.1492445	0.03882152	0.005
hsa-miR-375|AHR	0.3526464	0.008492691	0.001
hsa-miR-375|C1QBP	0.7467787	7.218683e-10	0.046
hsa-miR-375|INSM1	-0.1471285	0.313063	0.237
hsa-miR-375|MTPN	-0.2735746	0.0001232778	0.042
hsa-miR-375|MXI1	-0.3574628	0.0002895206	0.175
hsa-miR-375|PDK1	-0.3767964	7.171526e-08	0.016
hsa-miR-375|TIMM8A	-0.2276922	0.00149208	0.024
hsa-miR-375|USP1	-0.4230425	9.775618e-10	0.006
hsa-miR-375|YWHAZ	0.3467654	0.006128643	0.011
hsa-miR-376a*|PRPS1	0.2194553	0.07901059	0.249
hsa-miR-376a*|SLC16A1	-0.06988816	0.580126	0.194
hsa-miR-376a*|SNX19	0.1235271	0.3977687	0.286
hsa-miR-376a*|TTK	-0.2733567	0.02757653	0.035
hsa-miR-377|PAK1	-0.0980312	0.502781	0.073
hsa-miR-377|SOD1	0.1484146	0.3088048	0.318
hsa-miR-377|SOD2	0.2994086	0.03662093	0.097
hsa-miR-424|ANLN	-0.5776743	1.754141e-18	0.001
hsa-miR-424|ATF6	0.2182288	0.08172536	0.017
hsa-miR-424|CCND1	-0.4185318	0.002764278	0.025
hsa-miR-424|CCND3	-0.3393005	0.01707834	0.064
hsa-miR-424|CCNE1	-0.3714042	1.135029e-07	0.005
hsa-miR-424|CCNF	0.3887736	0.005768082	0.082
hsa-miR-424|CDC25A	0.09860838	0.4345156	0.159
hsa-miR-424|CDK6	-0.1901501	0.008248175	0.014
hsa-miR-424|CHEK1	0.3167971	0.02656847	0.181
hsa-miR-424|FGFR1	-0.05860338	0.6428578	0.244
hsa-miR-424|ITPR1	-0.529214	8.25478e-08	0.03
hsa-miR-424|KIF23	0.466014	0.0007391446	0.132

Table S2.Results obtained for the test on experimentally validated targets

hsa-miR-424|MAP2K1	0.4911719	4.711787e-13	0.115
hsa-miR-424|NFIA	0.3298461	2.981962e-06	0.019
hsa-miR-424|PIAS1	-0.2819862	7.41642e-05	0.004
hsa-miR-424|PLAG1	-0.08404762	0.5056381	0.269
hsa-miR-424|WEE1	0.07528708	0.5511396	0.093
hsa-miR-429|ERBB2IP	0.2310334	0.06407663	0.235
hsa-miR-429|KLHL20	-0.1821107	0.1465274	0.06
hsa-miR-429|PTPRD	-0.3587767	0.003337518	0.001
hsa-miR-429|RERE	0.2133191	0.08797777	0.564
hsa-miR-429|ZEB1	-0.3305143	0.007166393	0.009
hsa-miR-429|ZEB2	0.03756734	0.7663858	0.245
hsa-miR-433|GRB2	0.07722375	0.5979158	0.059
hsa-miR-449a|WISP2	-0.04650151	0.6442482	0.268
hsa-miR-451|CAB39	-0.02430098	0.7379458	0.037
hsa-miR-451|MIF	-0.1470045	0.04187896	0.038
hsa-miR-485-3p|NTRK3	-0.08146478	0.5188495	0.109
hsa-miR-486|FOXO1	-0.2491617	0.0842663	0.118
hsa-miR-491|TAF10	0.2260942	0.1182629	0.093
hsa-miR-494|PTEN	0.1169182	0.3536559	0.06
hsa-miR-503|ANLN	0.454511	0.001035447	0.082
hsa-miR-503|ATF6	0.2494466	0.08390038	0.111
hsa-miR-503|CCND1	-0.09742772	0.4659883	0.078
hsa-miR-503|CCNE1	-0.06379429	0.63554	0.058
hsa-miR-503|CCNE2	-0.1313445	0.2969886	0.168
hsa-miR-503|CCNF	0.2931199	0.01780444	0.076
hsa-miR-503|CDC25A	0.2178256	0.08131817	0.022
hsa-miR-503|CDKN1A	0.1091622	0.4552781	0.048
hsa-miR-503|CHEK1	0.2388407	0.05535702	0.179
hsa-miR-503|EIF2C1	0.1013131	0.4885172	0.207
hsa-miR-503|WEE1	-0.197922	0.1728141	0.19
hsa-miR-512-5p|MCL1	-0.09116643	0.533291	0.093
hsa-miR-516a-5p|KLK10	0.0968207	0.3354523	0.079
hsa-miR-519d|CDKN1A	-0.7749736	6.339812e-11	0.017
hsa-miR-520h|CDKN1A	-0.7829142	2.998039e-11	0.012
hsa-miR-520h|SMAD6	-0.04062731	0.7816542	0.341
hsa-miR-559|MTA1	-0.1434681	0.3253855	0.007
hsa-miR-7|EGFR	-0.001571122	0.9900897	0.001
hsa-miR-7|IRS1	-0.4301619	0.0003483637	0.131
hsa-miR-7|IRS2	-0.4690742	8.101943e-05	0.022
hsa-miR-7|PAK1	0.3237832	0.02323855	0.088
hsa-miR-7|RAF1	0.03751578	0.7980117	0.027
hsa-miR-7|SNCA	0.2952452	0.03944405	0.071
hsa-miR-92b|CDKN1C	0.3429291	0.005166281	0.181
hsa-miR-93|CDKN1A	-0.6951722	2.980356e-08	0.004
hsa-miR-93|MAPK9	0.3685167	1.446439e-07	0.01
hsa-miR-93|PCAF	-0.2742624	0.0001183331	0.001
hsa-miR-93|TP53INP1	-0.1667968	0.1881501	0.065
hsa-miR-96|ADCY6	0.4393938	1.818479e-10	0.018
hsa-miR-96|AQP5	0.002899795	0.9770401	0.151
hsa-miR-96|CDKN1A	-0.6627125	2.131955e-07	0.008
hsa-miR-96|CELSR2	0.2840491	0.0479327	0.072
hsa-miR-96|FOXO1	0.458442	5.707158e-11	0.001
hsa-miR-96|IRS1	-0.1065605	0.1412652	0.035

Table S2.Results obtained for the test on experimentally validated targets

hsa-miR-96|MITF	0.1383353	0.055681	0.208
hsa-miR-96|MYRIP	-0.1403145	0.05223732	0.008
hsa-miR-96|ODF2	-0.587397	9.125955e-06	0.003
hsa-miR-96|RYK	0.05376723	0.7136786	0.011
hsa-miR-98|E2F2	0.4671765	0.0007139184	0.027
hsa-miR-98|HMGA2	-0.4918842	0.0003312756	0.121
hsa-miR-98|MYC	-0.3619929	0.01059243	0.051
hsa-miR-99a|FGFR3	-0.2318209	0.001214646	0.009
hsa-miR-99a|IGF1R	0.1108882	0.3791958	0.13
hsa-miR-9|BACE1	-0.24934	0.0004872965	0.016
hsa-miR-9|BCL6	-0.09479417	0.3480055	0.06
hsa-miR-9|CDH1	0.1534705	0.1517663	0.022
hsa-miR-9|ETS1	-0.2442359	0.000640206	0.009
hsa-miR-9|FOXO1	-0.3192012	6.400257e-06	0.056
hsa-miR-9|MMP13	0.08214996	0.4140987	0.147
hsa-miR-9|NFKB1	-0.4746489	0.0005694184	0.004
hsa-miR-9|ONECUT2	0.2036257	0.004613694	0.001
hsa-miR-9|POU2F2	-0.4176189	0.002830167	0.013
hsa-miR-9|PRDM1	-0.06254635	0.3887687	0.115
hsa-miR-9|RAB34	-0.6513947	4.006499e-07	0.001
hsa-miR-9*|RCOR1	-0.1142264	0.1297812	0.024
hsa-miR-9|REST	-0.135335	0.06125764	0.104
