# Supplementary material for: Antagonism Pattern Detection between MicroRNA and Target Expression in Ewing’s Sarcoma
Source: PLoS One. 2012 Jul 25;7(7):e41770. doi: 10.1371/journal.pone.0041770 (PMC3404966; doi:10.1371/journal.pone.0041770)
Supplement: Table S3 — (A) List of miRNA hubs in the antagonism based network of Ewing’s sarcoma. (B) List of target hubs in the antagonism based network of Ewing’s sarcoma. (PDF) [file pone.0041770.s005.pdf]

Table S3A

Antagonism_miRNA_hub	Degree_connectivity
hsa-miR-342	1255
hsa-miR-146b	1203
hsa-miR-15a	1123
hsa-miR-324-3p	1040
hsa-miR-18a	1038
hsa-miR-486	1036
hsa-miR-328	1036
hsa-miR-582	1003
hsa-miR-25	986
hsa-miR-20b	984
hsa-miR-574	979
hsa-miR-363	978
hsa-miR-425-5p	961
HS_301	947
hsa-miR-192	937
hsa-mir-454-3p	925
hsa-miR-652	921
hsa-miR-92	915
hsa-miR-26a	915
hsa-miR-96	902
hsa-miR-125a	886
hsa-miR-30b	884
hsa-let-7i	883
hsa-miR-210	840
hsa-miR-93	837
hsa-miR-221	837
hsa-miR-339	836
hsa-miR-16	835
hsa-miR-423	834
hsa-miR-142-3p	815
hsa-miR-222	811
hsa-miR-484	804
hsa-miR-15b	801
hsa-miR-30e-5p	790
hsa-miR-594	788
hsa-miR-145	787
hsa-miR-28	781
hsa-miR-143	781
hsa-miR-194	778
hsa-miR-30d	776
hsa-miR-191	776
hsa-miR-29c	775
hsa-miR-518a	773
hsa-miR-148a	768
hsa-miR-148b	757
hsa-miR-768-3p	749
hsa-miR-576	742
HS_100	740
hsa-miR-185	738
hsa-miR-193a	734
hsa-miR-223	731

hsa-miR-598	728
hsa-miR-130b	728
hsa-miR-22	724
hsa-miR-199b	720
hsa-miR-183	720
hsa-miR-550	713
hsa-miR-30e-3p	702
hsa-let-7d	697
hsa-miR-19a	696
hsa-miR-106b	691
hsa-let-7f	690
hsa-miR-31	687
hsa-miR-99b	685
hsa-miR-19b	679
hsa-miR-374	677
hsa-miR-296	675
hsa-miR-193b	675
hsa-miR-130a	674
hsa-miR-323	663
hsa-miR-361	657
hsa-miR-30c	653
hsa-miR-186	651
hsa-miR-20a	648
hsa-miR-326	641
HS_192.1	641
hsa-miR-7	629
hsa-miR-18a*	620
hsa-miR-199a	619
hsa-miR-125b	618
hsa-miR-182	616
hsa-miR-320	615

Table S3B

Antagonism_Target_hub	Degree_connectivity
HFE	88
PDLIM5	80
PTGER3	78
CFLAR	71
SULF1	70
ZFR	69
MPZL1	69
MAP4	68
GM2A	66
CTSB	65
MCAM	63
GTSE1	63
TPM1	62
SFRS14	62
PATZ1	62
TCF3	61
QKI	61
PAFAH1B1	61
MARCKS	61
THBS1	60
PBXIP1	60
EXOC7	60
CD44	59
ATG5	59
UBE2J1	58
SMARCA4	58
RUNX1	58
LAMA2	58
ITGB5	58
EPB41L3	58
GOSR2	57
CYLD	57
PIAS1	56
MAX	56
APLP2	56
TRO	55
TMF1	55
PPIC	55
PITPNA	55
CLN5	55
CALD1	55
SOCS5	54
PDE4DIP	54
PAPSS2	54
ESR1	54
EGFR	54
SYNJ2	53
S100A11	53
LOX	53
GRB10	53
VDR	52

RIOK3	52
PTPN11	52
PDLIM7	52
NFIB	52
LAMA4	52
IL6ST	52
GNL3L	52
FGFR2	52
DAB2	52
CNOT4	52
SEC14L1	51
SCD	51
PALLD	51
EIF5B	51
DR1	51
CYP1B1	51
CSNK1A1	51
BUB1	51
SS18	50
SPTBN1	50
SMAD3	50
SDC2	50
KRIT1	50
COL1A1	50
CD58	50
CCPG1	50
BCLAF1	50
ABCF2	50
YWHAZ	49
TTC3	49
TRIM33	49
TIA1	49
RRBP1	49
RBM25	49
PCMT1	49
PAM	49
MINK1	49
KPNA1	49
JAG1	49
HEY1	49
GRN	49
CTBP2	49
COL1A2	49
VAMP2	48
UTX	48
TRAK1	48
SSX2IP	48
RFC1	48
RAB31	48
PPAP2A	48
OMD	48
MTAP	48
MR1	48

	Table S3B
MAP1LC3B	48
GALNT1	48
DZIP3	48
CDKN1C	48
ZNF313	47
ZMYM2	47
WNK1	47
TCF7L2	47
SOX4	47
PSME3	47
PKNOX1	47
MAP2K5	47
COL3A1	47
CLCN3	47
ANK3	47
UBE2N	46
TRIM2	46
TNPO1	46
TJP1	46
SP100	46
SIP1	46
PRKAR1A	46
PBX1	46
NUMA1	46
LGALS8	46
FGFR1	46
DPYSL4	46
COL6A1	46
CMKLR1	46
CD84	46
CD59	46
AKT3	46
ABCA1	46
VIL2	45
TMEM47	45
SENP6	45
RAB1A	45
PUM1	45
PROSC	45
PPIL2	45
PHF3	45
PCBP2	45
PAPOLA	45
NRG1	45
NPM1	45
NFATC1	45
H2AFY	45
EMP1	45
AHNAK	45
TOR1AIP1	44
THY1	44
THBD	44
SRP72	44

SMTN	44
SMARCA2	44
PTP4A1	44
NIPBL	44
NBN	44
NAP1L1	44
MUC1	44
LYN	44
LHFPL2	44
LAMP2	44
GPM6B	44
FER1L3	44
EML1	44
ATP2A2	44
ASAH1	44
ALDH3A2	44
WDFY3	43
USP34	43
UPF3A	43
TRAM2	43
SYNCRIP	43
SLC24A1	43
SEMA3F	43
QRSL1	43
PPIG	43
PGK1	43
PEBP1	43
PDCD6	43
NSFL1C	43
NDRG2	43
MED6	43
LAMP1	43
HADHA	43
GLS	43
EPHB2	43
DYNC2LI1	43
DNAJB6	43
COL5A3	43
COL5A2	43
CEP57	43
ARL4C	43
ARF4	43
API5	43
YIPF6	42
SLC7A8	42
PKNOX2	42
PCM1	42
NCLN	42
MCF2L	42
LMNA	42
LAMC1	42
ITSN1	42
IL13RA1	42

HLA-DRB1	42
HLA-C	42
CSE1L	42
ASF1A	42
APOE	42
ANGPTL2	42
ALDH6A1	42
WWTR1	41
TIMP3	41
SCP2	41
RUNX1T1	41
PTPRN2	41
PRSS23	41
PBX2	41
NRIP1	41
MLH3	41
MET	41
MACF1	41
HNRPD	41
HEXIM1	41
HEMK1	41
GSN	41
GNAS	41
GGA2	41
GART	41
FYN	41
FKBP1A	41
ENDOD1	41
DLG3	41
CUL5	41
COL4A2	41
CHMP2B	41
CDC73	41
BGN	41
AUTS2	41
ALMS1	41
ADD3	41
ZMYM6	40
WDR59	40
TNXB	40
SUPT7L	40
SLC29A1	40
SLC11A2	40
SAT1	40
PPP2R1B	40
PLXND1	40
PHACTR2	40
PARVA	40
NPAS2	40
NOL3	40
NFYC	40
MTMR1	40
MLL	40

Table S3B	
LPP	40
KIAA0494	40
KDELR3	40
ICAM1	40
HNRPDL	40
HMGB1	40
HLA-DQB1	40
GNA11	40
FEM1B	40
DPT	40
CYB561	40
BRD2	40
ASPH	40
ARF6	40
ANK2	40
ACLY	40
UROD	39
TM4SF1	39
SPARC	39
SIRT3	39
SERINC3	39
SENP3	39
RYBP	39
RPN2	39
RERE	39
RBPMS	39
PTBP1	39
PECAM1	39
OGT	39
NUFIP1	39
MINA	39
ME1	39
LRRFIP1	39
HIST1H2BD	39
GULP1	39
GOLGA2	39
GALNT10	39
FAS	39
DST	39
DDX19A	39
DAZAP2	39
CDYL	39
CD164	39
BAT2D1	39
ATP5L	39
AKAP1	39
WDR61	38
WASL	38
UBAP2L	38
TNS1	38
TM2D1	38
STAT1	38
SSR1	38

SNX13	38
SMARCA1	38
SLC35E1	38
SHMT2	38
SFRS11	38
SCAMP1	38
RRAS2	38
PTPRF	38
PTAFR	38
PICALM	38
PCSK5	38
PAK2	38
OBSL1	38
NR2F2	38
NCOA2	38
MYH10	38
MCM4	38
LPL	38
KLHL24	38
KCTD12	38
HEG1	38
GIMAP5	38
FAP	38
EXOSC7	38
ETV5	38
EPN2	38
ENTPD1	38
ELOVL2	38
ELK1	38
EIF4E2	38
DBT	38
CX3CL1	38
CUTL1	38
CPD	38
COL5A1	38
COL11A1	38
CES2	38
CDK10	38
CDC42EP1	38
CALU	38
ATP2B4	38
AGA	38
ABHD2	38
WSB1	37
WIPI1	37
WBP4	37
VAMP3	37
UBXD2	37
U2AF2	37
TTLL5	37
TRIO	37
TPD52	37
TOMM40	37

TNS3	37
TNFRSF10B	37
SPTAN1	37
SP110	37
SMARCC1	37
SELPLG	37
RHOQ	37
REEP5	37
RBM8A	37
RAB33A	37
PPP3CB	37
PPP3CA	37
PPP2R4	37
PKN2	37
PIGV	37
PEX3	37
PEA15	37
PDE4A	37
PDCD4	37
PCOLCE	37
PAFAH2	37
NUAK1	37
NEDD9	37
MPHOSPH9	37
IGF2BP3	37
HSPG2	37
HRK	37
HMGCS1	37
GTF2F1	37
FGF1	37
ENC1	37
EML3	37
EEF1D	37
DKK3	37
DDAH2	37
DCN	37
CUGBP1	37
CEP63	37
CEACAM1	37
ATP6V1D	37
ATP5S	37
ARIH1	37
ADCY9	37
VEZF1	36
TBXA2R	36
STK38L	36
SSH3	36
SNX1	36
SLC6A8	36
SFRP1	36
RNF24	36
RNF128	36
PRKCH	36

PRKCDBP	36
PRDM2	36
PLAU	36
PDGFB	36
NR3C1	36
MYO1B	36
METTL7A	36
MEF2C	36
MBNL1	36
MBD2	36
MAF	36
LDLR	36
JTB	36
HTATIP	36
ENSA	36
DDX18	36
CSRP2	36
CNN3	36
CLU	36
CHD4	36
CASP1	36
CA12	36
ATP2C1	36
ARF3	36
YTHDC1	35
YME1L1	35
TSR1	35
TMEM118	35
TGOLN2	35
TFAM	35
TEGT	35
TCF25	35
TCEA2	35
STAB1	35
SMYD2	35
SMARCA5	35
SMAD7	35
SKP1A	35
SAP18	35
RHEB	35
RECQL	35
RASGRP2	35
PTN	35
PRPF4B	35
PLAUR	35
P4HA2	35
NFYA	35
MZF1	35
MAFG	35
LSM5	35
LEPR	35
LAPTM5	35
KCNJ5	35

ILF3	35
HRBL	35
HNRPH3	35
GPR116	35
GOLGB1	35
GARNL1	35
FBLN1	35
FAM13A1	35
DSTN	35
DOPEY1	35
DLG1	35
DIMT1L	35
CTTN	35
CTNND1	35
CPT1A	35
CPSF6	35
CD200	35
CAMK2G	35
ANXA4	35
AKAP13	35
WWOX	34
UBE4B	34
UBE2D2	34
TROVE2	34
TNKS	34
TLE2	34
TBC1D9B	34
STOM	34
STC2	34
SMARCD1	34
SFRS2B	34
SFPQ	34
SET	34
SEMA6A	34
SASH1	34
RTN4	34
RPS6KA2	34
RNF10	34
RBM5	34
PTPRD	34
PSPC1	34
PSG9	34
PLOD2	34
PIP5K1A	34
OAZ2	34
NOL5A	34
NCOA4	34
MTCP1	34
MBNL2	34
LAPTM4B	34
KLHDC3	34
KLF9	34
KDELR2	34

ITM2A	34
IQGAP1	34
IL8	34
HPCAL1	34
HLA-F	34
HLA-E	34
GRSF1	34
GOLGA1	34
FBXO38	34
FBXO28	34
ENPP2	34
EIF1AY	34
DTX3	34
DKC1	34
CYBB	34
CUL4B	34
CREB1	34
CPNE3	34
CIB2	34
CDH1	34
CD40	34
CALCA	34
APC	34
AGTRL1	34
ADARB1	34
ADAM12	34
ZNHIT4	33
ZNF710	33
ZFP36L2	33
ZFP161	33
YY1	33
WTAP	33
WDR48	33
WDR23	33
VPS13D	33
TGFBR2	33
TFPI	33
SYT11	33
SVEP1	33
SLC16A3	33
SLC16A1	33
SKP2	33
SF3A1	33
SCARB2	33
RUFY3	33
RPL22	33
RBM12B	33
RAD23B	33
RAD17	33
PTRF	33
PLEC1	33
PGRMC1	33
PDGFRA	33

OPTN	33
NFATC2IP	33
NF1	33
NAV3	33
MYO6	33
MYH9	33
MPDZ	33
MLLT10	33
MKI67	33
MGEA5	33
MCM3AP	33
MAPK14	33
MAP4K5	33
MAGI1	33
KIAA0090	33
KDELC1	33
KCNJ8	33
ITGBL1	33
IGFBP7	33
HRH1	33
HAPLN1	33
GPSM2	33
GPR107	33
GLUL	33
FXR1	33
FTL	33
FN1	33
ESPL1	33
ELMO2	33
EDG4	33
CYP2C9	33
CPT2	33
CHN2	33
CHN1	33
CEACAM21	33
CDV3	33
CD46	33
CAST	33
CASP10	33
CAMSAP1	33
CAMK2B	33
ASXL1	33
ARMC8	33
ARFIP1	33
APP	33
APBA2	33
ALDH7A1	33
AKAP11	33
AIF1	33
ADAMTS2	33
ZNF536	32
ZNF337	32
ZNF224	32

WHSC1	32
VAMP1	32
UBE3A	32
TMCO1	32
THBS2	32
SYNGR1	32
STX16	32
STAT6	32
SFRS10	32
SFI1	32
SCARB1	32
RXRB	32
RGS3	32
RASA1	32
PTPN22	32
PRMT2	32
PPP2R5C	32
PPP1CB	32
PHF20	32
PDGFA	32
OTUB1	32
OPCML	32
NOTCH3	32
NEK3	32
NAB2	32
MRPS31	32
MEG3	32
MEF2A	32
LTBP1	32
LST1	32
KYNU	32
KIAA1704	32
KCNQ1	32
ITM2B	32
IFRD1	32
HNMT	32
HLA-B	32
GPC1	32
GGA1	32
GATA2	32
GAS7	32
FOLR2	32
ETV1	32
ENO1	32
EEF1E1	32
DYRK2	32
DDX28	32
CSDE1	32
COL6A2	32
COL14A1	32
CLTC	32
CLEC4A	32
CKAP4	32

Table S3B

CENTD3	32
CD93	32
BUB3	32
BRAP	32
BCAP29	32
BAZ2A	32
BAT1	32
ATG12	32
ARHGAP25	32
AQP1	32
APPBP2	32
APH1B	32
AP2A2	32
ANXA7	32
ACTR3	32
ACTN1	32
ACSL3	32
ACOX1	32
ZNF667	31
ZBTB20	31
VGLL4	31
ULK2	31
TRIM23	31
TRAM1	31
TP53I3	31
TOM1L1	31
TMEM66	31
TMED9	31
TLE1	31
TFAP2A	31
TCP1	31
TBX2	31
TBL1X	31
TAX1BP1	31
SVIL	31
STARD8	31
SRPX2	31
SPAG9	31
SOX9	31
SOX11	31
SLC7A6	31
SLC28A1	31
SFRS5	31
SERBP1	31
SEC63	31
SEC24D	31
S100A4	31
RNF13	31
RFK	31
RAP2C	31
RAMP3	31
RAD50	31
RABL4	31

RABGGTB	31
RABGAP1	31
PWP1	31
PRUNE	31
PPFIA1	31
PLXNC1	31
PGF	31
PCSK6	31
PARVB	31
NOL7	31
MXI1	31
MTMR2	31
MMP2	31
MED8	31
MCL1	31
MAP3K5	31
MAG	31
LRRFIP2	31
LPHN1	31
LIMK2	31
KIAA1033	31
KIAA0652	31
JUN	31
HLF	31
HLA-G	31
HIVEP2	31
GNB1	31
GLP1R	31
FUS	31
FOLH1	31
FBXO21	31
ENG	31
EIF4B	31
EGR1	31
DNAJC4	31
DCTD	31
CPE	31
CHST3	31
ARL1	31
ARFGEF1	31
APOL2	31
ZNF395	30
ZFP36L1	30
WAPAL	30
UMPS	30
UBR2	30
UBE2I	30
TXNIP	30
TSPAN15	30
TP53AP1	30
TMEM4	30
TMEM111	30
TLOC1	30

TGFBI	30
TFG	30
TEAD4	30
TAX1BP3	30
TAF6L	30
TACC1	30
STX12	30
SQLE	30
SPON1	30
SPN	30
SPG7	30
SOS1	30
SMCHD1	30
SLC6A2	30
SLC39A1	30
SLC1A4	30
SLC15A2	30
SFRS2IP	30
SERPINB6	30
SEPHS1	30
SEC31A	30
SAMD4A	30
RNASET2	30
RBL2	30
RARA	30
PVR	30
PTPRC	30
PTPN12	30
PPM1F	30
PPIB	30
PPAP2B	30
PLEKHA5	30
PHLDA1	30
PHKB	30
PHF11	30
PAX3	30
OSBPL3	30
OCRL	30
NQO1	30
NOTCH2	30
NEK11	30
NCOR1	30
MIA3	30
MAP4K4	30
LIMS1	30
KRT19	30
KRAS	30
KIAA0859	30
JAKMIP2	30
IVD	30
ITSN2	30
ITGA6	30
IRS2	30

IL1RL1	30
IL17RC	30
IGFBP5	30
IGF1	30
IAPP	30
HSPA4	30
HSP90B1	30
HLA-DRA	30
HINT1	30
GYG1	30
GTF2I	30
GPR172A	30
GNS	30
GDPD5	30
FSCN1	30
FLI1	30
FHL1	30
FGF18	30
EPOR	30
EIF5	30
EIF1	30
DZIP1	30
DUSP6	30
CYFIP2	30
CXCR4	30
CUL2	30
CTDSP2	30
CPSF1	30
CHST5	30
CEACAM6	30
CDC2L5	30
CCND2	30
BIRC5	30
BCL2	30
AURKA	30
AP2S1	30
ADAM22	30
ABCG1	30
ZNF223	29
UBE2H	29
UBE2B	29
TRAFD1	29
TPP2	29
TMEM97	29
TMEM1	29
THRA	29
TFRC	29
TAPBP	29
SUB1	29
STK24	29
SNAP23	29
SLCO2B1	29
SLC25A36	29

SFRS8	29
RNF41	29
RGS5	29
RARB	29
RANBP9	29
PUM2	29
PREPL	29
POLG	29
PMS2L11	29
PLEKHC1	29
PIGL	29
PHTF1	29
PDK2	29
PASK	29
OLFML2B	29
NKTR	29
NEK1	29
NDUFA5	29
MME	29
LUM	29
LRDD	29
KIF20A	29
KIAA1539	29
JMJD2B	29
IGHD	29
IGF2	29
HTRA1	29
HNRPH1	29
HIST1H2AJ	29
HEPH	29
GTPBP4	29
GPX3	29
GPC4	29
GCDH	29
GAS6	29
FBXO11	29
EZH1	29
EDG2	29
EBAG9	29
DICER1	29
DDR1	29
DCUN1D4	29
CYP3A4	29
CUGBP2	29
CSDA	29
COPB2	29
COL4A1	29
COL18A1	29
CIRBP	29
CEP152	29
CDH5	29
CD55	29
CD300A	29

CAPN3	29
BNIP3	29
B3GAT3	29
ATP8A2	29
ATF7IP	29
ARTN	29
APBB2	29
AP2B1	29
AHCYL1	29
ABHD6	29
ABCC6	29
ZNF638	28
ZNF609	28
ZNF451	28
ZNF292	28
YLPM1	28
WIZ	28
WDR45	28
VAMP4	28
TXN	28
TTLL3	28
TREX1	28
TRADD	28
TPI1	28
TNPO2	28
TMEM80	28
TDRD3	28
TCIRG1	28
TBL1XR1	28
TBC1D12	28
SULT1A1	28
SUCLG2	28
STAG2	28
SPTLC1	28
SORBS3	28
SNRK	28
SLIT3	28
SLC35E2	28
SH3BP2	28
SFRS7	28
SFRS3	28
RUNX3	28
RSL1D1	28
RPS6KB1	28
RPA1	28
RNASE4	28
RFX5	28
RANBP5	28
RAD23A	28
RAB11FIP2	28
PSMA7	28
PSIP1	28
PSD3	28

PPP3CC	28
PPIF	28
PPFIBP1	28
PPFIA3	28
PNPLA4	28
PLXNA2	28
PLA2G4A	28
PDK3	28
PDGFRL	28
PCGF2	28
PARP8	28
OSBPL10	28
OLFM1	28
OAS2	28
NUDT4	28
NTRK2	28
NT5E	28
NR4A1	28
NNT	28
NEK2	28
NCK1	28
MGP	28
MFAP5	28
MARS	28
MAPT	28
MAPRE2	28
MAOA	28
MAGI2	28
LSM14A	28
LRPPRC	28
LOXL2	28
LEF1	28
LCMT2	28
KLHL20	28
JAG2	28
IVNS1ABP	28
IFT122	28
HNRPA3	28
HMGCR	28
HBS1L	28
GOLGA8B	28
GNPTAB	28
GNG11	28
GNAI1	28
GLYAT	28
GCLC	28
GATAD1	28
GAPVD1	28
FBN1	28
EXOSC2	28
EPB41L2	28
ELN	28
EHD2	28

DLGAP1	28
DLG2	28
CYB5A	28
CTDSPL	28
CLTA	28
CLASP2	28
CFDP1	28
CDC27	28
CD63	28
CCNG2	28
CALML4	28
BTN3A1	28
AZIN1	28
ASMTL	28
ARHGEF15	28
ARHGEF10	28
ANK1	28
ALDH5A1	28
AK2	28
ABCC10	28
ABAT	28
ZNF587	27
ZNF45	27
ZNF3	27
ZDHHC17	27
WIPI2	27
WEE1	27
VDAC3	27
VCAM1	27
USP3	27
UPK1B	27
UNC84A	27
UBOX5	27
UBE3B	27
TTK	27
TRAK2	27
TNIP2	27
TNFAIP1	27
TMEM43	27
TMED5	27
TMCC1	27
TGIF2	27
STS	27
STOML1	27
SSBP2	27
SPATS2	27
SPATA5L1	27
SPAST	27
SLC39A8	27
SLC16A5	27
SKAP2	27
SH3BP5	27
SGSH	27

SERINC5	27
SEMA3C	27
SEC24A	27
SCG5	27
SATL1	27
SART3	27
SAR1A	27
SALL2	27
RSF1	27
RRN3	27
RHOBTB3	27
RAB5A	27
RAB11FIP3	27
PSME4	27
PRKCA	27
PPP1R10	27
PLAGL1	27
PGRMC2	27
PGLS	27
PEX10	27
PDIA6	27
PABPN1	27
PA2G4	27
NUP50	27
NFATC3	27
NCOA1	27
NADK	27
MSR1	27
MRS2L	27
MICAL2	27
MATR3	27
LYST	27
LTBP4	27
LMCD1	27
LIAS	27
LDB3	27
LARP5	27
KSR1	27
KPNB1	27
KCTD2	27
ITPR2	27
ITGB3	27
ITGB1	27
INTS7	27
INHBC	27
IFI16	27
HOMER3	27
HD	27
GSTA1	27
GRIN1	27
GDF10	27
GAS2L1	27
FZD2	27

Table S3B	
FXR2	27
FOXN2	27
FNDC3A	27
FKBP1B	27
FARP1	27
FAM8A1	27
EXT2	27
EPHB4	27
EIF4G3	27
EIF2S1	27
DPYD	27
DOK5	27
DNAJB9	27
DGUOK	27
DERL1	27
DDX3Y	27
DCPS	27
CYP3A5	27
CYP2E1	27
CTSS	27
CSF1	27
CRKRS	27
CRK	27
CDH11	27
CD151	27
CCND1	27
CAV2	27
CASP8	27
CASP4	27
CACNA1G	27
BNIP3L	27
BCL11A	27
BAT3	27
B4GALT5	27
B4GALNT1	27
ATM	27
ARPC2	27
ARL6IP5	27
ANP32A	27
ALS2CR8	27
ALK	27
AKAP8L	27
ADCY3	27
ADA	27
ACCN2	27
ABI1	27
ABCD4	27
ZNF354A	26
ZNF131	26
YWHAQ	26
XRCC4	26
WWP1	26
WDR68	26

WDR19	26
WDR1	26
TOB2	26
TGM4	26
TCF12	26
TBC1D17	26
TAOK2	26
TANK	26
TAF9	26
TACC2	26
SYNPO	26
SYDE1	26
STK17A	26
STC1	26
SPTLC2	26
SNCA	26
SLC4A7	26
SLC35D1	26
SLC33A1	26
SLC23A2	26
SLC19A1	26
SLC16A7	26
SGCE	26
SFXN3	26
SEZ6L	26
SEC23B	26
RMND5A	26
RING1	26
RIN3	26
RFTN1	26
PTPN1	26
PSCD4	26
PRKACB	26
PPP1R2	26
PPP1R12A	26
POU6F1	26
PODXL	26
PNPLA2	26
PLXNA1	26
PLA2G6	26
PKP4	26
PINK1	26
PIGO	26
PHKA1	26
PEX11A	26
PDE4C	26
PDE3B	26
PDE1C	26
OPA1	26
NXN	26
NUDT13	26
NRP1	26
NNMT	26

NFIC	26
NFATC4	26
NEK9	26
NCOA3	26
MYL4	26
MYCBP	26
MTUS1	26
MTSS1	26
MTMR4	26
MTMR11	26
MRPL9	26
MMP14	26
MIPEP	26
MFN1	26
MBD4	26
MAST2	26
MAPK8IP3	26
MAP3K7IP2	26
MAP1A	26
MAGED1	26
KLF6	26
KLF12	26
KIAA1467	26
KIAA0913	26
KIAA0368	26
KAL1	26
IPO7	26
IMPDH2	26
IMPACT	26
ICA1	26
HOXB7	26
GPR177	26
GOLGA4	26
GNG7	26
GHITM	26
GABRR1	26
FOSL2	26
FMR1	26
FLOT1	26
FLNA	26
FASTK	26
FAM69A	26
EXOSC4	26
ETF1	26
EPS15	26
EPHA5	26
EED	26
DPF3	26
DOCK6	26
DIP2C	26
DDX17	26
DAG1	26
CTSC	26

CTNNA1	26
CRKL	26
COX11	26
COL4A6	26
CITED2	26
CHST2	26
CHMP4A	26
CDH2	26
CDC42BPA	26
CD47	26
CCNA1	26
CC2D1A	26
CBFA2T2	26
CAV1	26
BCL2L1	26
ATP6AP2	26
ATG4A	26
ARIH2	26
AGPAT1	26
ADD2	26
ADAM19	26
ACSM3	26
ACRV1	26
ZMYM4	25
ZFAND6	25
ZBTB1	25
WDR42A	25
VCP	25
TXNDC4	25
TTC27	25
TSPAN4	25
TRAF3IP3	25
TPM4	25
TMEM9B	25
TMCO6	25
TCF4	25
TBCE	25
TBC1D2B	25
STAT2	25
SRPK2	25
SRI	25
SRD5A1	25
SPSB3	25
SOX18	25
SOX15	25
SOD2	25
SNRPB	25
SMC4	25
SLC35A3	25
SLC35A2	25
SLC11A1	25
SLAMF8	25
SIRPA	25

SHC1	25
SH2B1	25
SFTPC	25
SATB2	25
S100A13	25
RTF1	25
RPS6KA3	25
ROD1	25
RNASE1	25
RHOD	25
RER1	25
RB1CC1	25
RARG	25
RAP1GDS1	25
PSMC2	25
PRKAR2A	25
PRKAB1	25
PRDX2	25
PQBP1	25
PPP2CB	25
PPFIBP2	25
PPBP	25
POLDIP3	25
PLSCR1	25
PDZRN3	25
PCSK7	25
ORC5L	25
ODZ4	25
NTRK3	25
NRF1	25
NGLY1	25
NDST1	25
NCAM1	25
MTRF1L	25
MTMR9	25
MSH3	25
MKRN1	25
METTL3	25
METAP2	25
MCF2	25
LSS	25
LRP12	25
LOH11CR2A	25
LARP1	25
L3MBTL	25
KMO	25
KIAA1109	25
KIAA0226	25
KCNQ2	25
KATNB1	25
IPO4	25
INPP1	25
IFT88	25

HSDL2	25
HOXB6	25
HIST1H2BK	25
HIPK3	25
HERC4	25
HDHD1A	25
GTF2IRD1	25
GNB5	25
GNAI3	25
GMFG	25
GLRX	25
GJA4	25
GFPT1	25
GDF11	25
FKBP4	25
FKBP11	25
FGFR1OP	25
FAM117A	25
EML2	25
ELOVL4	25
ELK4	25
EDNRB	25
EDA	25
DUOX1	25
DNAJB4	25
DFFA	25
DDX3X	25
DBI	25
CTBS	25
CTBP1	25
CREBBP	25
CORO1C	25
COL15A1	25
COL13A1	25
CIZ1	25
CFH	25
CEP164	25
CENPF	25
CENPA	25
CDR2L	25
CCDC93	25
CAP1	25
CA2	25
C1QTNF1	25
C1QB	25
BLVRA	25
ATRNL1	25
ATP6V1C1	25
ATP6V1A	25
ATP5C1	25
ATP2B1	25
ARHGEF12	25
ARHGAP29	25

ARHGAP1	25
ANP32E	25
ANGPT1	25
ANAPC5	25
AMD1	25
ACACB	25
ABI2	25
ZNF93	24
ZNF500	24
ZNF467	24
YES1	24
VPS24	24
USP9X	24
USP21	24
UNC13B	24
UBE2L3	24
UBE2G1	24
TSFM	24
TRIM38	24
TRIM3	24
TRIM10	24
TOM1	24
TNFRSF21	24
TMPO	24
TCEB1	24
TBC1D4	24
TBC1D22A	24
TAPBPL	24
TAP2	24
SUZ12P	24
SUPT3H	24
STCH	24
SSX3	24
SPRY1	24
SNRPA1	24
SMG7	24
SLC39A6	24
SLC38A2	24
SLC38A1	24
SLC25A23	24
SLC13A3	24
SIGLEC1	24
SFRS1	24
SF3B3	24
SETX	24
SERTAD2	24
RSBN1	24
RREB1	24
RPL31	24
ROBO1	24
RNASE6	24
RGS4	24
RDH8	24

RBM12	24
RBCK1	24
RAB7L1	24
PSMF1	24
PRR5	24
PRDX6	24
PPP4R2	24
PPP2CA	24
PNRC1	24
PNN	24
PMP22	24
PLS3	24
PLEKHB2	24
PIK3R1	24
PIK3CD	24
PCSK2	24
PARD3	24
PAPOLG	24
OXTR	24
OBSCN	24
NUSAP1	24
NPHP4	24
NOLC1	24
NCBP2	24
NAPA	24
MYO1D	24
MXRA5	24
MUT	24
MTDH	24
MRPS18B	24
MPI	24
LYPLA1	24
LRFN3	24
LMBRD1	24
LGR5	24
LDHB	24
LASS6	24
KLHL9	24
KIAA0372	24
KIAA0182	24
KIAA0101	24
KCNG1	24
INPP5A	24
IMPAD1	24
IL1B	24
HMGCL	24
HLA-DPA1	24
HEXA	24
HERPUD1	24
HAP1	24
GPR137	24
GPHN	24
GLG1	24

GJA1	24
GEMIN4	24
GATM	24
FRYL	24
EP300	24
EHMT2	24
EEF1G	24
EDNRA	24
ECD	24
DOK4	24
DLAT	24
DIAPH2	24
DDEF1	24
DAP	24
CYB5B	24
CSNK1E	24
CREBL2	24
CRAT	24
COX5B	24
COX15	24
CLTB	24
CLCC1	24
CHERP	24
CEP350	24
CELSR2	24
CEACAM7	24
CDK2	24
CDC42EP3	24
CCDC6	24
CARS	24
CARD10	24
CAMK1G	24
CACNA1A	24
BRF2	24
BMP6	24
BMP1	24
BCL10	24
AYTL2	24
ATP13A3	24
ATN1	24
ATF2	24
ASPN	24
ARHGDIA	24
AMFR	24
ALDOA	24
ACTB	24
A2M	24
ZNF518	23
ZDHHC3	23
ZDHHC11	23
YIPF3	23
WDTC1	23
UTY	23

USP24	23
USP14	23
UBXD7	23
UBL3	23
UBE2E1	23
UBE1L	23
TSPAN31	23
TRIM9	23
TNRC4	23
TNC	23
TMEM30A	23
TLE3	23
THUMPD1	23
TBK1	23
TAF1	23
SYT5	23
ST7	23
SSH1	23
SNX3	23
SNX10	23
SMC1A	23
SLIT2	23
SLC7A1	23
SH3GL3	23
SFXN1	23
SEMA4F	23
SARDH	23
RYK	23
RPS4Y1	23
RIMS3	23
RDX	23
RDH11	23
RBM23	23
RAPGEF5	23
RAD21	23
RABGAP1L	23
RAB26	23
PROS1	23
PRCP	23
POT1	23
POLD2	23
POGZ	23
PLXDC1	23
PLK4	23
PLD1	23
PHLPPL	23
PEX16	23
PDPN	23
PDLIM2	23
PDIA2	23
PDCL	23
PDCD2	23
PCDH9	23

PCBP4	23
OAS1	23
NUDT21	23
NUBPL	23
NSDHL	23
NR2C1	23
NOV	23
NEU1	23
NASP	23
MUC3B	23
MTA1	23
MT1M	23
MS4A1	23
MRP63	23
MMP9	23
MED12	23
MCFD2	23
MAN2A2	23
MADD	23
LRRC1	23
KRR1	23
KIAA1305	23
KIAA0692	23
JMJD3	23
ITCH	23
IL27RA	23
IKBKE	23
IGFBP4	23
HYAL1	23
HMG2L1	23
GUF1	23
GRK5	23
GPRC5B	23
GLIPR1	23
GGPS1	23
GALC	23
FYCO1	23
FRZB	23
FNDC3B	23
FBXW11	23
FBXO3	23
FAM86C	23
F12	23
EWSR1	23
EPB41L1	23
EPAS1	23
ENOSF1	23
EIF4G1	23
EHD4	23
EFS	23
DSCR3	23
DNAJC10	23
DNAJA1	23

DGCR8	23
CYP2A6	23
CXCL2	23
CUL3	23
CTAGE5	23
CRHR1	23
COL16A1	23
COG5	23
CNOT2	23
CHD9	23
CHD3	23
CDH8	23
CDC42EP4	23
CDC34	23
CAMSAP1L1	23
CACNA1I	23
C1QA	23
BTG1	23
BRWD1	23
BCL3	23
ATXN10	23
ATXN1	23
ATP9A	23
ATP1B3	23
ATF5	23
ARMET	23
ARHGEF4	23
APOBEC3B	23
ALDH2	23
ALDH18A1	23
AGPAT7	23
AEBP1	23
ADNP	23
ZNF576	22
ZNF167	22
ZNF132	22
ZMYM5	22
ZFAND5	22
ZBTB7A	22
ZBTB16	22
WDR79	22
VEZT	22
UTS2	22
UBE2O	22
UBE1L2	22
TUBB2C	22
TTC33	22
TSTA3	22
TSPO	22
TSPAN8	22
TSN	22
TRIP13	22
TRAPPC3	22

TOMM70A	22
TNFSF10	22
TIMELESS	22
TFDP1	22
TDG	22
TBC1D8	22
TARDBP	22
TAF9B	22
SYT1	22
SYPL1	22
SUPT6H	22
STX7	22
STAM2	22
STAG1	22
SRRM2	22
SNTB2	22
SNN	22
SLC30A1	22
SKIL	22
SHQ1	22
SH3BGRL	22
SEMA4C	22
SCHIP1	22
SAMM50	22
RRAGD	22
RNF6	22
RNF14	22
RNF103	22
RNASEH1	22
RETSAT	22
RCN3	22
RCBTB2	22
RBM3	22
RBBP6	22
RARRES2	22
RANBP1	22
RALBP1	22
RALB	22
RAC2	22
PVRL1	22
PURA	22
PTPRA	22
PTK2B	22
PPP2R3A	22
PLK2	22
PLEK	22
PKM2	22
PIK3CA	22
PIAS2	22
PHF16	22
PFDN5	22
PEX19	22
PEX1	22

PCNX	22
PBX3	22
PAICS	22
OLFML1	22
OGFR	22
NUP210	22
NUP188	22
NMT2	22
NMD3	22
NFKBIA	22
NEO1	22
NCR3	22
NAB1	22
MYO1C	22
MXD3	22
MT1X	22
MRC2	22
MMRN2	22
MGAT1	22
MFAP2	22
MAPK13	22
MAP4K1	22
LEPROTL1	22
KRT17	22
KIF2A	22
KIAA0232	22
KIAA0040	22
KCNMA1	22
KCNH2	22
IRAK1	22
INSIG1	22
IL2RB	22
IGSF3	22
IGFBP3	22
IGF1R	22
HSPD1	22
HSBP1	22
HNF4A	22
HMGA1	22
HIPK2	22
HIPK1	22
HCFC1R1	22
GTPBP2	22
GOLGA5	22
GLT25D2	22
GJB3	22
GIT2	22
GAPDHS	22
GALNT2	22
FZD1	22
FTSJ3	22
FTH1	22
FRMD4A	22

FLRT2	22
FKBP8	22
FCGR2C	22
FCGR1A	22
FBXL11	22
FANCA	22
FAM46A	22
F8	22
EVC	22
EP400	22
ELOVL1	22
EI24	22
DOK1	22
DNAJB14	22
DIO2	22
DHX15	22
DDEFL1	22
DALRD3	22
CSNK2A2	22
CRYBB2	22
COLQ	22
COL21A1	22
CMTM6	22
CMAH	22
CLEC3B	22
CDC40	22
CDC25B	22
CD74	22
CARD8	22
BMP8A	22
BHLHB2	22
BCAT1	22
ATP5G3	22
ATF7IP2	22
ARMCX2	22
APTX	22
AP1S1	22
AKR7A2	22
AFF2	22
ADAM17	22
ACTR2	22
ACTN2	22
ACOT9	22
ABCB9	22
ABCA2	22
ZXDC	21
ZNF589	21
ZNF544	21
ZNF289	21
ZNF274	21
ZNF217	21
ZNF177	21
ZGPAT	21

ZFP2	21
ZC3HAV1	21
WSB2	21
WDR13	21
WAC	21
VIM	21
VAMP5	21
USP11	21
UGCG	21
TYMS	21
TXNDC1	21
TUBB3	21
TUBB2B	21
TRPV5	21
TRPC1	21
TRMT12	21
TREM2	21
TPM2	21
TOR1A	21
TNFRSF1A	21
TMEM176B	21
TMEM16K	21
TMEM131	21
TMEM100	21
TKT	21
TGFA	21
TFEB	21
TFAP2B	21
SYK	21
STX2	21
SSX2	21
SPHK1	21
SPCS3	21
SOX13	21
SNCAIP	21
SNAP25	21
SMPD1	21
SMOX	21
SMC3	21
SMC2	21
SLITRK5	21
SLC2A10	21
SLA	21
SKAP1	21
SIRT5	21
SIRT1	21
SFRS9	21
SFRS6	21
SFRS2	21
SF1	21
SERPINB1	21
SAV1	21
S100A6	21

RSU1	21
RNH1	21
RHOH	21
RHOBTB2	21
RGS2	21
RGS16	21
RFC5	21
RBKS	21
RANGAP1	21
RAB11A	21
PTX3	21
PTPN4	21
PTP4A3	21
PTP4A2	21
PTGS1	21
PTCH1	21
PSMB1	21
PRELP	21
PPT1	21
PPP2R5A	21
PPM1B	21
POMP	21
PODXL2	21
PMAIP1	21
PLEKHG3	21
PLAGL2	21
PIP5K1B	21
PHKA2	21
PEX5	21
PELO	21
PEF1	21
PDXK	21
PDLIM1	21
PDGFRB	21
PDE6B	21
PCTK1	21
PCNXL2	21
PCDHB6	21
PCDH7	21
PAWR	21
PAPPA2	21
PAK3	21
PACRG	21
ORC4L	21
NUDT6	21
NTS	21
NDUFB2	21
NARG2	21
MYST4	21
MYH11	21
MXRA8	21
MRPL2	21
MLF1IP	21

MGLL	21
MGAT2	21
MDH2	21
MDC1	21
MAZ	21
MASP2	21
LRP1	21
LCP1	21
LANCL1	21
KIF2C	21
KIAA0562	21
KIAA0423	21
KIAA0280	21
KIAA0240	21
KHDRBS1	21
JUND	21
JAM3	21
ITGAV	21
ISG20	21
IL1RAP	21
IL1R1	21
IL15RA	21
IKBKB	21
IFNGR1	21
IFITM1	21
ICAM3	21
HTATSF1	21
HPX	21
HOXA10	21
HLA-DPB1	21
HLA-DMB	21
HIGD1A	21
HHLA3	21
HDAC7A	21
H2AFX	21
GYG2	21
GUCY1A3	21
GRK6	21
GPR56	21
GPR153	21
GPI	21
GLT8D2	21
GCH1	21
GAS1	21
GABARAPL2	21
FZR1	21
FRY	21
FOXJ3	21
FAM89B	21
FAM20B	21
FAM120A	21
F2RL1	21
EXTL3	21

EPS8	21
ELK3	21
ELF4	21
EIF2S2	21
EFNA1	21
ECM2	21
ECM1	21
E2F3	21
DVL2	21
DRD2	21
DPYSL3	21
DPY19L4	21
DPP4	21
DLC1	21
DISC1	21
DHTKD1	21
DHPS	21
DFNB31	21
DDHD2	21
DAPK1	21
CSTF2T	21
COQ7	21
COBLL1	21
CLIC3	21
CLDN5	21
CHRNA3	21
CHAF1A	21
CENTD1	21
CDC25A	21
CD99	21
CD34	21
CCR1	21
CCL18	21
CCDC69	21
CASP7	21
CASK	21
CAPZB	21
CAPN2	21
BTF3	21
BRIP1	21
BMPR1A	21
ATP9B	21
ATP1B1	21
ARVCF	21
AKAP9	21
AES	21
ADAM10	21
ACOT7	21
ACBD4	21
ACACA	21
ABCG2	21
ABCC1	21
ZNF197	20

ZNF155	20
ZFHX4	20
ZC3H13	20
ZBTB48	20
YTHDC2	20
XPNPEP1	20
XBP1	20
WNT6	20
WDR74	20
VPS35	20
VEGFC	20
USP33	20
USP25	20
UPF3B	20
UNC84B	20
TUSC3	20
TULP4	20
TSPAN6	20
TSEN2	20
TRIP4	20
TNFAIP3	20
TMSB10	20
TMCC2	20
TLK2	20
TGM2	20
TERF1	20
TCOF1	20
TCF7L1	20
TBL2	20
TAGLN2	20
SRPX	20
SRPK1	20
SOX2	20
SMPDL3A	20
SMARCC2	20
SMAD1	20
SLCO2A1	20
SLC25A6	20
SLC25A44	20
SHANK2	20
SETD2	20
SERPING1	20
SCYL3	20
SCYE1	20
SCRN3	20
RNPS1	20
RND3	20
RLF	20
RIPK5	20
RGS6	20
RFC3	20
RCC1	20
RBBP5	20

RANBP10	20
RAI2	20
PVRL3	20
PTPRM	20
PTPRK	20
PTGER4	20
PTCRA	20
PSMD9	20
PSMD12	20
PRNP	20
PRKD3	20
PRKCZ	20
PRKCD	20
PRKACA	20
PPP1R3D	20
PPP1R3C	20
PPME1	20
POU3F1	20
PON2	20
POLR3C	20
POLR2K	20
POLR2E	20
PET112L	20
PDE8A	20
PDE4D	20
PCLO	20
PARP3	20
PAIP1	20
OSBPL2	20
OSBPL1A	20
OLFML2A	20
NUTF2	20
NPAL3	20
NIPSNAP1	20
NFYB	20
NFRKB	20
NFE2L1	20
NBPF10	20
NARF	20
NAGA	20
MYBL2	20
MT1F	20
MSH6	20
MPPED2	20
MOSC2	20
MON1B	20
MMP11	20
MLF1	20
MFNG	20
MFN2	20
MEGF9	20
ME2	20
MAST4	20

MAFB	20
LXN	20
LUC7L2	20
LTK	20
LRRC47	20
LRP10	20
LPIN2	20
LAT	20
LAIR1	20
KRT18	20
KLK2	20
KIF3B	20
KIAA1199	20
KIAA0776	20
KCNS3	20
KCNK1	20
JOSD1	20
ITPK1	20
ITGA4	20
ITGA2	20
IRF5	20
IRF3	20
INTS6	20
INHBA	20
IGHG1	20
IGF2R	20
IFT57	20
IFITM2	20
ICK	20
HYI	20
HTATIP2	20
HOXD3	20
HMGN4	20
HMG20B	20
HIP2	20
HIP1R	20
HIP1	20
HDLBP	20
HDDC2	20
H2AFV	20
GMPPB	20
GLT25D1	20
GGTLA1	20
GGCX	20
GCAT	20
FIP1L1	20
FAT	20
EVI5	20
EPM2AIP1	20
EPHB1	20
EMP3	20
ELF2	20
EHBP1	20

EFHD1	20
ECT2	20
ECH1	20
DUT	20
DUSP3	20
DNAJB5	20
DMD	20
DLX4	20
DHRS2	20
DENR	20
DENND4C	20
DCAKD	20
DBN1	20
CXCL12	20
CXCL10	20
CTPS2	20
CSK	20
CRIP2	20
CREB3L1	20
COL6A3	20
CHPT1	20
CFI	20
CDK2AP1	20
CDC5L	20
CD53	20
CCT6A	20
CASP9	20
CAPNS1	20
CAND1	20
CALCRL	20
BTD	20
BMPR2	20
BLZF1	20
BAG2	20
BACH2	20
B2M	20
AVPI1	20
ARSA	20
ARHGEF9	20
ARHGAP24	20
APOM	20
ANXA11	20
ANPEP	20
ANKRD28	20
ANKRD26	20
ANKRD12	20
ANGEL2	20
ALG5	20
ADRA1D	20
ADIPOR1	20
ADCY2	20
ACP6	20
ACBD3	20

ZNF79	19
ZNF665	19
ZNF573	19
ZNF434	19
ZNF423	19
ZNF335	19
ZNF330	19
ZNF211	19
ZMAT4	19
ZCCHC2	19
ZC3H14	19
YWHAB	19
WDR33	19
WBP11	19
USP46	19
USP4	19
USP22	19
USP15	19
USP1	19
UBXD6	19
UBE2D3	19
TXNDC13	19
TUBD1	19
TST	19
TSPAN7	19
TRAF4	19
TNIK	19
TMEM176A	19
TMEM127	19
TMED2	19
TMED10	19
TK2	19
TES	19
TCF20	19
TAT	19
TAF1A	19
SURF1	19
SUPT4H1	19
STIP1	19
ST3GAL6	19
SSPN	19
SRR	19
SREBF2	19
SNX2	19
SNX19	19
SLCO3A1	19
SLC7A11	19
SLC39A9	19
SLC30A5	19
SLC16A2	19
SLC10A3	19
SIX2	19
SIRT2	19

SIM2	19
SIL1	19
SFRS15	19
SFN	19
SERINC1	19
SEPP1	19
SEL1L	19
SEC22A	19
SDHB	19
SAP30	19
SAFB	19
RXRA	19
RRM2	19
RPL41	19
RPL36	19
RPH3AL	19
ROCK1	19
RNF11	19
RHO	19
RFX3	19
RECQL5	19
RBMX2	19
RBM34	19
RASL12	19
RASGRP3	19
RAB14	19
PTPRE	19
PTPN2	19
PTPN18	19
PTGDS	19
PSMD11	19
PRR7	19
PRKCSH	19
PPP2R1A	19
PPBPL2	19
POU2F1	19
POLQ	19
PMM2	19
PLSCR3	19
PLEKHF2	19
PLCB1	19
PLAT	19
PJA2	19
PIN4	19
PHLDA3	19
PHF2	19
PER2	19
PER1	19
PDE4B	19
PDE2A	19
PCYOX1	19
PCDHGA3	19
PCCA	19

P4HB	19
OXR1	19
OSBPL8	19
ORMDL2	19
OLFML3	19
NUP98	19
NUP54	19
NUCB1	19
NSUN5B	19
NRL	19
NR6A1	19
NR4A3	19
NPY5R	19
NPTN	19
NPEPL1	19
NFASC	19
NET1	19
NENF	19
NDUFB8	19
NAGK	19
MYCBP2	19
MXD4	19
MRPL15	19
MPP2	19
MORC2	19
MOCS2	19
MFHAS1	19
MEIS2	19
MEGF8	19
MBTPS1	19
MBD5	19
MASP1	19
MARK2	19
MAPRE3	19
MANSC1	19
MANEA	19
MAN2B2	19
MAN1B1	19
MAN1A2	19
MAGED2	19
M6PRBP1	19
LTBP2	19
LRRC23	19
LMO4	19
LEPREL1	19
LCP2	19
LBR	19
KPNA3	19
KLHL7	19
KIAA1609	19
KIAA0974	19
KIAA0513	19
KCND1	19

ISL1	19
IFI44	19
ICOSLG	19
HOXD13	19
HOXB5	19
HK2	19
HIBCH	19
HDAC4	19
HBP1	19
H6PD	19
GTPBP1	19
GTF3C2	19
GSPT1	19
GSK3A	19
GRK4	19
GPX5	19
GPR124	19
GP9	19
GORASP2	19
GORASP1	19
GOLPH3L	19
GNG4	19
GINS4	19
GFOD2	19
GABBR2	19
GAB2	19
FXYD5	19
FTO	19
FSTL1	19
FBXO17	19
FBXL5	19
FASTKD2	19
FAM3C	19
EIF2B1	19
EGR2	19
EDN2	19
ECHDC3	19
DYSF	19
DYRK1A	19
DYNC1LI2	19
DLG5	19
DIRAS3	19
DGCR2	19
DCX	19
DCLRE1C	19
DCHS1	19
CXCR7	19
CRTC1	19
COX7C	19
COPZ2	19
COPS8	19
COL10A1	19
CNOT8	19

CHRDL1	19
CHIC2	19
CEP76	19
CDS2	19
CDK7	19
CDC42EP2	19
CDC2L6	19
CDC25C	19
CCNT2	19
CCNI	19
CCNF	19
CCNA2	19
CCDC71	19
CCDC48	19
CBX4	19
CAMTA2	19
CALM3	19
CACNB3	19
BUB1B	19
BTG2	19
BRCA1	19
BCL2L13	19
B4GALT1	19
AVIL	19
ASIP	19
ARAF	19
AP3S1	19
AMPD1	19
ALAS2	19
AKAP7	19
AK5	19
AFF1	19
ADSL	19
ADH5	19
ADAMDEC1	19
ACVR1B	19
ACP1	19
ACCN1	19
ABL1	19
ABHD5	19
AASDHPPT	19
ZNF672	18
ZNF43	18
ZNF384	18
ZNF281	18
ZNF232	18
ZNF193	18
ZNF134	18
ZKSCAN1	18
ZIC3	18
ZFX	18
ZC3H7B	18
ZC3H3	18

ZBTB24	18
YKT6	18
WDR62	18
WBP2	18
VPS13A	18
USF2	18
UGCGL1	18
TYROBP	18
TYRO3	18
TXNDC9	18
TXNDC5	18
TWSG1	18
TUBGCP5	18
TSPY1	18
TSNAXIP1	18
TSC22D1	18
TRIM68	18
TRIM29	18
TRIM14	18
TRAPPC6A	18
TRAP1	18
TPP1	18
TPMT	18
TP53	18
TNK1	18
TNFRSF25	18
TMOD3	18
TMEM63A	18
TMEM41B	18
TMEM123	18
TLL2	18
TIMM17A	18
TIE1	18
TDRKH	18
TCFL5	18
TCEAL4	18
TBC1D15	18
SULT4A1	18
STXBP6	18
STMN1	18
STK10	18
STAU2	18
ST6GAL1	18
SSB	18
SPEN	18
SPDEF	18
SPCS2	18
SPARCL1	18
SPAG5	18
SNW1	18
SLC7A7	18
SLC4A4	18
SLC43A3	18

SLC39A14	18
SLC25A37	18
SLC22A4	18
SLC22A17	18
SIN3B	18
SH3TC1	18
SGCB	18
SFTPB	18
SF3A2	18
SETMAR	18
SESN1	18
SDCBP	18
SCYL2	18
SCLY	18
SCAMP2	18
SBF1	18
SATB1	18
S100A10	18
RWDD3	18
RRAS	18
RPS6KA5	18
RPL5	18
RPL37	18
RNF8	18
RNF139	18
RHOT2	18
RHOC	18
REPIN1	18
RBM13	18
RASL11B	18
RASAL1	18
RARRES1	18
RAI14	18
RAE1	18
RAD51C	18
RAD51AP1	18
RABIF	18
RABEP1	18
RAB40B	18
RAB22A	18
RAB21	18
RAB20	18
QDPR	18
PYHIN1	18
PTPN7	18
PTGIS	18
PTBP2	18
PSCD3	18
PRKD1	18
PRKCB1	18
PPP4C	18
POLR1D	18
POLG2	18

PLVAP	18
PLGLA1	18
PLEKHQ1	18
PLCB4	18
PLA2G4B	18
PKN1	18
PIWIL2	18
PIGP	18
PHF20L1	18
PFTK1	18
PFKFB3	18
PEX7	18
PEX14	18
PDZD3	18
PDE9A	18
PDCD10	18
PCP4	18
PAPPA	18
OSGEPL1	18
OGN	18
OGG1	18
NUP88	18
NUCKS1	18
NR1I2	18
NPFF	18
NPC2	18
NLGN4X	18
NKX3-1	18
NGFRAP1	18
NFE2L2	18
NAPG	18
NAGLU	18
MYO15B	18
MYH2	18
MVK	18
MTFR1	18
MTF2	18
MS4A6A	18
MRPS30	18
MNAT1	18
MLXIP	18
MGAT5	18
MERTK	18
MEOX2	18
MEF2D	18
MCM7	18
MAPKAPK2	18
MAP3K4	18
MAP3K12	18
MAP2K4	18
MAK10	18
LZTS1	18
LY86	18

LSG1	18
LRRC17	18
LRCH4	18
LITAF	18
LIMK1	18
LDLRAP1	18
LARP4	18
LAMA5	18
KRT35	18
KLK13	18
KLHL21	18
KLF4	18
KIAA1598	18
KIAA0174	18
KIAA0141	18
KIAA0100	18
KBTBD10	18
JAK1	18
ITPR3	18
ITPR1	18
ITGAM	18
ITFG1	18
IER5	18
HSF2	18
HRASLS	18
HOXD4	18
HK1	18
HIST1H2AC	18
HIF1AN	18
HDAC6	18
HADH	18
GTF3A	18
GSTM1	18
GRLF1	18
GNG12	18
GLB1	18
GHR	18
GBP2	18
GABBR1	18
FXYD6	18
FUBP1	18
FOXM1	18
FEZ1	18
FCGR2A	18
FBXO9	18
FAM38B	18
FAHD2A	18
ETHE1	18
ERP29	18
ERF	18
EPHB3	18
EIF4E	18
EDEM1	18

E2F8	18
DUSP11	18
DTX4	18
DTL	18
DOCK4	18
DNMT1	18
DND1	18
DNAJC9	18
DDX27	18
DDX25	18
DDOST	18
DARS2	18
DAK	18
CTSO	18
CSTF1	18
CSNK1G3	18
CRIM1	18
CORO2A	18
CORO1A	18
COPS5	18
COMT	18
COL8A2	18
CNOT3	18
CLEC11A	18
CLDN10	18
CHMP2A	18
CETN2	18
CERK	18
CEP290	18
CEBPD	18
CDKN2B	18
CDC6	18
CDC2	18
CD97	18
CD82	18
CD3D	18
CCT2	18
CCNJL	18
CASP6	18
CAMK2N1	18
CALCOCO1	18
C1QBP	18
BTN3A3	18
BTBD14B	18
BMP2K	18
BLM	18
BDH1	18
BCL7A	18
BCKDHB	18
BCAS3	18
BAIAP2	18
BAG3	18
BACE1	18

AXIN1	18
AUH	18
ATXN7	18
ATP5E	18
ASPM	18
ASAHL	18
ARSE	18
ARRB2	18
ARHGEF11	18
ARHGEF10L	18
ARFIP2	18
APOL1	18
APBA3	18
ANKRD15	18
AMT	18
AMPD2	18
ALCAM	18
AKR1C2	18
ADCY7	18
ADAT1	18
ACOX3	18
ACOT11	18
ABCE1	18
ABCC3	18
AAK1	18
ZZEF1	17
ZWINT	17
ZNF350	17
ZNF266	17
ZNF146	17
ZFP37	17
ZCCHC4	17
ZBED1	17
YIPF4	17
YIPF1	17
YBX1	17
XPNPEP2	17
WFDC1	17
WDR5B	17
VASP	17
VAPB	17
UNC45A	17
UCHL1	17
UBE2G2	17
TUBB6	17
TSPYL1	17
TRRAP	17
TRPS1	17
TRPM3	17
TRIM24	17
TRAF3	17
TPBG	17
TOMM20	17

TNK2	17
TNFAIP8	17
TMEM33	17
TMEM2	17
TMEM106C	17
TM7SF3	17
TLR5	17
TIAL1	17
TGFB1I1	17
TENC1	17
TBC1D9	17
TBC1D5	17
TAGLN	17
SYNJ1	17
SUMO3	17
ST3GAL2	17
SPAG16	17
SOX10	17
SORBS2	17
SON	17
SNRP70	17
SMURF2	17
SLC38A3	17
SLC22A18	17
SLC19A2	17
SHOX2	17
SETD3	17
SERPINB2	17
SEMA4A	17
SCRN1	17
SCPEP1	17
SCAMP3	17
SBNO1	17
S100PBP	17
RSRC1	17
RPP38	17
RNMTL1	17
RNF122	17
RIMS1	17
RIMBP2	17
RHBDD3	17
RFC2	17
RCHY1	17
RBP4	17
RBM7	17
RBM19	17
RAB4A	17
QTRT1	17
PXDN	17
PTGES	17
PSG3	17
PSAP	17
PRX	17

PRODH	17
PRC1	17
PQLC3	17
PPP5C	17
PPP1R7	17
PPIA	17
POP1	17
PNMA2	17
PLCL2	17
PLCG2	17
PITPNC1	17
PILRA	17
PIK3R4	17
PHF15	17
PEX12	17
PDHB	17
PDE1A	17
PDCD6IP	17
PCDH12	17
PCBD1	17
P2RX4	17
OVGP1	17
OSTM1	17
OPRS1	17
NUP43	17
NUP107	17
NSUN5	17
NRBP1	17
NRBF2	17
NR2F6	17
NPR1	17
NP	17
NOS1AP	17
NONO	17
NMT1	17
NME5	17
NLE1	17
NINJ2	17
NEIL3	17
NDUFA6	17
NDST2	17
NCKAP1L	17
NCAM2	17
MYO9B	17
MYO5C	17
MYLIP	17
MUC7	17
MTRF1	17
MT1G	17
MSN	17
MRPL13	17
MPDU1	17
MEA1	17

MDFIC	17
MAPKAPK3	17
MAPK8	17
MAP3K6	17
MAN2A1	17
MALT1	17
LSM4	17
LSM1	17
LRRC15	17
LEMD3	17
LCAT	17
LAMB2	17
LAMB1	17
KLK3	17
KLF11	17
KIAA0355	17
KIAA0247	17
KCTD5	17
KCNJ2	17
ITM2C	17
ITIH4	17
ITGAX	17
ITGA7	17
IRF4	17
INTS8	17
ING2	17
IL17B	17
IFT20	17
IFNA10	17
IER2	17
HTR3A	17
HSPE1	17
HSP90AB1	17
HSD17B8	17
HS2ST1	17
HPCAL4	17
HOXD9	17
HMGN3	17
HLA-DMA	17
HLA-A	17
HIC2	17
HES1	17
HERC1	17
HCK	17
H2BFS	17
H2AFZ	17
GUCY1B3	17
GSTO1	17
GRPEL1	17
GRIK5	17
GPR4	17
GOLGA8A	17
GNE	17

GLT8D1	17
GLS2	17
GLI3	17
GGA3	17
GCNT2	17
GBP1	17
GABARAP	17
G6PC3	17
FNBP1L	17
FNBP1	17
FER	17
FEN1	17
FEM1C	17
FCMD	17
FCER1G	17
FAT4	17
FASLG	17
FAM98A	17
FAM62A	17
FAM49A	17
FAM107A	17
FAIM	17
FADS3	17
EPHX2	17
EPHA3	17
EMCN	17
ELTD1	17
EIF2B5	17
EHBP1L1	17
EDG7	17
DPH5	17
DOCK9	17
DNAJC17	17
DMWD	17
DLG4	17
DLEC1	17
DKK2	17
DIAPH1	17
DHRS4	17
DDX49	17
DDX24	17
DDT	17
DDB2	17
DBF4	17
DAAM2	17
CYR61	17
CYP19A1	17
CXADR	17
CUL4A	17
CTSK	17
CTSF	17
CTNNAL1	17
CSF2	17

CRISP1	17
CRELD1	17
CPZ	17
CMPK	17
CLSTN2	17
CLPTM1	17
CKAP2	17
CIAPIN1	17
CHFR	17
CEP170	17
CDKN2C	17
CDKN1B	17
CDK5R1	17
CDH15	17
CDC7	17
CD83	17
CD4	17
CCNB2	17
CCHCR1	17
CCDC47	17
CBX8	17
CBX1	17
CBLL1	17
CBFB	17
CASP2	17
CARHSP1	17
CAPN7	17
CAPN6	17
CACNA2D2	17
C1S	17
BTN3A2	17
BTN2A2	17
BTK	17
BRD4	17
BRCA2	17
BOLA1	17
BMPER	17
BMP4	17
BMP2	17
BLK	17
BHLHB3	17
BCCIP	17
BCAT2	17
BAZ1A	17
BAHCC1	17
BACE2	17
B3GNTL1	17
B3GALNT1	17
ATP8A1	17
ATP6V1E1	17
ATP10D	17
ASTN2	17
ASRGL1	17

ASCC3	17
ARSB	17
ARMCX5	17
ARL2BP	17
ARHGAP28	17
ARC	17
AOX1	17
ANP32B	17
ANKRD27	17
ANKRD25	17
ANKRD17	17
AMOTL2	17
ALPL	17
ALDH4A1	17
ALDH3B1	17
ALDH1L1	17
AIP	17
ADIPOR2	17
ACYP2	17
ACTR1A	17
ACTN4	17
ACOX2	17
ACAA1	17
ZNF76	16
ZNF506	16
ZNF430	16
ZNF410	16
ZNF282	16
ZNF26	16
ZNF135	16
ZNF124	16
ZFYVE9	16
ZCCHC14	16
YARS2	16
WHSC2	16
WFS1	16
WDR78	16
WARS	16
VWF	16
VRK1	16
VPS8	16
VPS41	16
VPS39	16
VAV2	16
VARS	16
UTP6	16
USP6	16
USP48	16
USP12	16
UQCRC2	16
UPF1	16
UBXD8	16
UBAP2	16

TXNRD2	16
TUBG2	16
TTRAP	16
TTLL1	16
TSPYL4	16
TRMT1	16
TRIOBP	16
TRIM8	16
TRIM66	16
TRIB3	16
TPST2	16
TPCN1	16
TNIP1	16
TNFAIP6	16
TMEM92	16
TMEM59	16
TMEM109	16
TM2D3	16
TH1L	16
TGFB3	16
TGDS	16
TFAP4	16
TESK2	16
TCF7	16
TCERG1	16
TBX5	16
TAZ	16
TARBP2	16
TAF4	16
TAF12	16
SYNGR2	16
SYCP1	16
SUOX	16
SULT1A2	16
SUHW4	16
STAT5B	16
STAP2	16
STAC	16
SSTR2	16
SSFA2	16
SSBP1	16
SRBD1	16
SPR	16
SOD1	16
SNX11	16
SNRPB2	16
SNAPC1	16
SNAI2	16
SMYD5	16
SMARCE1	16
SMAD2	16
SLC6A15	16
SLC28A2	16

SLC27A3	16
SLC24A6	16
SLC1A5	16
SLC17A7	16
SLC16A4	16
SGK	16
SFRP4	16
SETBP1	16
SERPINE1	16
SCG2	16
SCFD1	16
SAR1B	16
SAPS3	16
RUFY1	16
RPS9	16
RPP21	16
RPL4	16
RPL13A	16
RPL13	16
ROR1	16
ROCK2	16
ROBO3	16
RNF7	16
RNF123	16
RHAG	16
RGS1	16
RET	16
RBM38	16
RBM16	16
RASSF7	16
RASGRF1	16
RANBP2	16
RABGGTA	16
RAB27A	16
QTRTD1	16
PTTG1IP	16
PTPLAD1	16
PTPLA	16
PTMA	16
PSMD7	16
PSMA2	16
PSCD1	16
PRR11	16
PROX1	16
PRMT5	16
PRKCQ	16
PRG2	16
PRDM10	16
PQLC1	16
PPP2R2B	16
PPAT	16
POU2F2	16
POLS	16

POLL	16
PLSCR4	16
PLEKHJ1	16
PLA2G4C	16
PITRM1	16
PIP5K1C	16
PIK3C3	16
PIGT	16
PHTF2	16
PHLDA2	16
PFDN2	16
PCMTD2	16
PCDHB11	16
OTUD3	16
NSUN3	16
NPTXR	16
NME4	16
NIT1	16
NCDN	16
NCAPH2	16
NAV2	16
NARS	16
NADSYN1	16
MYST1	16
MYO1A	16
MYL9	16
MYL1	16
MTO1	16
MTIF2	16
MSRB2	16
MSL2L1	16
MRPS14	16
MRPL49	16
MN1	16
MMP24	16
MLYCD	16
MLC1	16
MKKS	16
MARK4	16
MAP9	16
MAP3K8	16
MAN1A1	16
MAEA	16
MAD1L1	16
LY6H	16
LTBP3	16
LRRC37A	16
LRBA	16
LPXN	16
LPGAT1	16
LMAN1	16
LIMD1	16
LIG4	16

LGR4	16
LGALS1	16
LAT2	16
LARP7	16
KPNA4	16
KLHL2	16
KLHL18	16
KIR3DL2	16
KIAA1641	16
KIAA0922	16
KIAA0831	16
KIAA0323	16
KIAA0241	16
KHSRP	16
KDR	16
KCNN3	16
KCNMB4	16
JRKL	16
ITGA5	16
IQSEC1	16
IQGAP2	16
IL5RA	16
IL21R	16
IL1RN	16
IGFBP6	16
IGBP1	16
IFT81	16
IFI6	16
IFI27	16
IBTK	16
HYAL2	16
HSD17B4	16
HR	16
HOXA7	16
HOMER1	16
HEY2	16
HCLS1	16
HCCS	16
H3F3B	16
GRAMD3	16
GPR45	16
GPR23	16
GPLD1	16
GPKOW	16
GPD1L	16
GNG5	16
GNAQ	16
GMPR2	16
GMCL1	16
GIPC1	16
GIMAP6	16
GFRA2	16
GFOD1	16

GATA3	16
GAD2	16
FZD7	16
FZD4	16
FUZ	16
FUT8	16
FUT6	16
FNTA	16
FNDC4	16
FLAD1	16
FKBP14	16
FGL1	16
FECH	16
FDX1	16
FBXO5	16
FBXO42	16
FBXL4	16
FBXL18	16
FBLN2	16
FAM60A	16
FAM5B	16
FAM53C	16
FAM3A	16
FAM35A	16
FAM18B	16
FAM119B	16
FAM102A	16
FAIM2	16
FAH	16
EYA2	16
EXTL2	16
EXOD1	16
ETNK2	16
ETNK1	16
ESR2	16
EPHX1	16
EPB49	16
ENTPD7	16
ENTPD4	16
EMG1	16
ELL2	16
ELF1	16
ELAVL1	16
EIF4EBP1	16
EIF2C4	16
EGFL6	16
EFNB2	16
EDC4	16
E2F4	16
E2F1	16
DYNLT3	16
DUSP22	16
DUSP1	16

DTNA	16
DOCK2	16
DNAJC7	16
DMXL2	16
DMXL1	16
DLL3	16
DLG7	16
DHX38	16
DHCR7	16
DEGS1	16
DDX23	16
DBP	16
DAPK3	16
CYP51A1	16
CYP46A1	16
CTSZ	16
CTSL2	16
CTGF	16
CST3	16
CSPG5	16
CSF1R	16
CREM	16
CREBL1	16
CPSF3L	16
CPM	16
COX7A1	16
COX4NB	16
COL7A1	16
COL2A1	16
CLSTN3	16
CLIC4	16
CLCN4	16
CLASP1	16
CIC	16
CGGBP1	16
CFTR	16
CEP250	16
CENPE	16
CDR2	16
CDO1	16
CDKN1A	16
CD6	16
CD48	16
CD180	16
CCDC102B	16
CBX7	16
CADPS2	16
CACYBP	16
CACNA1H	16
CACNA1B	16
CA8	16
C1QL1	16
BRD7	16

BEX1	16
BCAM	16
BAI2	16
ATP6V0C	16
ATP5I	16
ATP5B	16
ATP1B2	16
ATP10A	16
ATF7	16
ASPHD1	16
ASCC3L1	16
ARMCX1	16
ARHGEF3	16
ARHGEF17	16
AQP6	16
APLP1	16
APBA1	16
APAF1	16
AP1G2	16
AOAH	16
ANKRD10	16
ANGPT2	16
AMPD3	16
ALPPL2	16
ADD1	16
ADAM9	16
ACTA2	16
ACAA2	16
ABR	16
