# Supplementary material for: Antagonism Pattern Detection between MicroRNA and Target Expression in Ewing’s Sarcoma
Source: PLoS One. 2012 Jul 25;7(7):e41770. doi: 10.1371/journal.pone.0041770 (PMC3404966; doi:10.1371/journal.pone.0041770)
Supplement: Table S4 — P-values obtained for the seed enrichment analysis of the antagonism based network. (PDF) [file pone.0041770.s006.pdf]

TABLE S4								
**Seed length**	**Positive correlation seed count**	**Negative correlation seed count**	**Antagonism pattern seed count**	**Mean randomized pairs seed count**	**Stdev randomized pairs seed count**	**p-value positive correlation**	**p-value negative correlation**	**p-value antagonism pattern**
8-mer	362	415	383	313	20	0.01	1.30E-007	1.93E-004
7-mer	1517	1588	1520	1249	41	1.04E-011	0	1.92E-011
6-mer	7270	7449	7180	5907	137	0	0	0
tot	9149	9452	9083	7469	198	0	0	2.22E-016
