# Supplementary material for: Antagonism Pattern Detection between MicroRNA and Target Expression in Ewing’s Sarcoma
Source: PLoS One. 2012 Jul 25;7(7):e41770. doi: 10.1371/journal.pone.0041770 (PMC3404966; doi:10.1371/journal.pone.0041770)
Supplement: Table S5 — (A) GO analysis results for antagonism based target sets. (B) GO analysis results for target sets based on both antagonism and miRNA seed occurrence. (C) GO analysis results for correlation based target sets. (D) GO analysis results for target sets based on both correlation and miRNA seed occurrence. (PDF) [file pone.0041770.s007.pdf]

TABLE S5A	Gene Ontology Analysis of antagonism based target genes			
	**GO term description**	**# Total annotated genes**	**# Antagonism linked genes**	**PV ($-log_{10}$)**
**hsa-miR-1**				
	sarcomere	14	6	8.44
	myofibril	19	6	7.50
	contractile fiber part	23	6	6.95
	contractile fiber	25	6	6.71
	striated muscle contraction	14	5	6.62
	structural constituent of muscle	33	6	5.95
**hsa-miR-127**				
	M phase of mitotic cell cycle	85	15	4.88
	mitosis	82	14	4.43
**hsa-miR-133a**				
	sarcomere	14	5	5.82
	myofibril	19	5	5.09
	structural constituent of muscle	33	6	5.01
	contractil fiber part	23	5	4.65
	contractil fiber	25	5	4.46
**hsa-miR-142-3p**				
	cellular protein complex disassembly	13	8	5.17
	protein complex disassembly	14	8	4.84
	macromolecular complex disassembly	15	8	4.55
**hsa-miR-142-5p**				
	RNA processing	174	26	4.39
	cellular protein complex disassembly	13	6	4.11
**hsa-miR-143**				
	RNA processing	174	36	5.40
**hsa-miR-148b**				
	collagen	23	312	6.80
	proteinaceous extracellular matrix	98	25	5.85
	extracellular matrix	100	25	5.67
	extracellular matrix part	57	17	5.14
**hsa-miR-155**				
	protein ubiquitination	40	8	4.07
**hsa-miR-186**				
	collagen	23	9	4.50
**hsa-miR-206**				
	sarcomere	14	6	7.27
	myofibril	19	6	6.35
	structural constituent of muscle	33	7	6.03
	contractil fiber part	23	6	5.80
	contractil fiber	25	6	5.57
	straited muscle contraction	14	4	4.18
**hsa-miR-222**				
	collagen	23	10	4.59

TABLE S5A	(continue 2/2) - Gene Ontology Analysis of antagonism based target genes			
	**GO term description**	**# Total annotated genes**	**# Antagonism linked genes**	**PV $(-log_{10})$**
**hsa-miR-29b**				
	cell cycle process	193	33	6.83
	cell cycle phase	170	29	6.95
	mitotic cell cycle	153	26	5.45
	M phase of mitotic cell cycle	85	18	5.31
	cell cycle	315	41	5.08
	M phase	114	21	5.07
	mitosis	82	16	4.32
**hsa-miR-30b**				
	collagen	23	10	4.26
**hsa-miR-30d**				
	proteinaceous extracellular matrix	98	25	5.65
	extracellular matrix	100	25	5.48
	extracellular matrix part	57	17	4.99
	collagen	23	10	4.75
**hsa-miR-324-3p**				
	collagen	23	12	5.30
	extracellular matrix part	57	19	4.45
	transmembrane receptor protein kinase activity	51	17	4.04
**hsa-miR-328**				
	collagen	23	11	4.46
**hsa-miR-329**				
	collagen	23	10	5.92
**hsa-miR-361**				
	collagen	23	9	4.47
**hsa-miR-369-3p**				
	mitotic spindle organization and biogenesis	10	5	4.46
	spindle organization and biogenesis	11	5	4.42
**hsa-miR-379**				
	extracellular matrix part	57	13	4.91
**hsa-miR-410**				
	extracellular matrix	100	19	4.89
	proteinaceous extracellular matrix	98	18	4.45
	collagen	23	8	4.29
**hsa-miR-512-3p**				
	striated muscle contraction	14	5	4.11
**hsa-miR-532**				
	microtubule	32	9	4.46
**hsa-miR-582**				
	RNA splicing	92	28	5.69
**hsa-miR-655**				
	regulation of anatomical structure morphogenesis	26	8	4.55
**hsa-miR-656**				
	phospholipid transporter activity	12	5	4.84
**hsa-miR-92b**				
	collagen	23	8	4.01

TABLE S5B	Gene Ontology analysis of target genes based on both antagonism and miRNA seed occurrence			
	GO term description	# Total annotated genes	# Antagonism linked genes	PV $(-log_{10})$
**hsa-miR-1**				
	sarcomere	14	4	5.82
	myofibril	19	4	5.24
	contractile fiber part	23	4	4.89
	contractile fiber	25	4	4.74
**hsa-miR-130a**				
	lysosomal membrane	10	5	4.36
	vacuolar membrane	11	5	4.11
**hsa-miR-142-5p**				
	mRNA processing	74	12	4.41
	RNA processing	174	19	4.09
	RNA splicing	92	13	4.09
**hsa-miR-148b**				
	collagen	23	9	5.48
	proteinaceous extracellular matrix	98	18	4.83
	extracellular matrix	100	18	4.70
**hsa-miR-155**				
	small conjugating protein binding	12	4	4.02
**hsa-miR-186**				
	collagen	23	8	4.41
**hsa-miR-192**				
	protein serine threonine phosphatase complex	10	5	4.07
**hsa-miR-30d**				
	proteinaceous extracellular matrix	98	18	4.34
	collagen	23	8	4.23
	extracellular matrix	100	18	4.22
**hsa-miR-494**				
	collagen	23	7	4.49
**hsa-miR-532**				
	microtubule	32	7	4.45

TABLE S5C	Gene Ontology Analysis of correlation based target genes			
	**GO term description**	**# Total annotated genes**	**# Correlation linked genes**	**PV** $(-log_{10})$
**hsa-miR-1**				
	structural constituent of muscle	33	9	12.86
	striated muscle contraction	14	7	12.27
	regulation of muscle contraction	19	7	11.12
	contractile fiber part	23	7	10.44
	contractile fiber	25	7	10.15
	sarcomere	14	6	10.05
	myofibril	19	6	9.10
	regulation of multicellular organism process	151	10	7.88
	system process	563	15	6.35
	actin cytoskeleton	129	8	6.14
	structural molecule activity	244	10	5.92
	regulation of heart contraction	25	4	4.91
	cytoskeleton	368	10	4.34
**hsa-miR-126**				
	transmembrane receptor protein kinase activity	51	7	6.94
	transmembrane receptor protein tyrosine kinase activity	43	6	6.05
	protein kinase activity	63	6	5.06
	transmembrane receptor activity	419	12	4.08
**hsa-miR-126***				
	extracellular matrix part	57	5	4.95
	cell cell adhesion	86	5	4.08
**hsa-miR-130b**				
	cell cycle	315	29	14.46
	cell cycle process	193	23	13.84
	mitotic cell cycle	153	20	12.82
	cell cycle phase	170	20	11.94
	regulation of cell cycle	182	18	9.50
	M phase	114	14	8.70
	mitosis	82	12	8.41
	M phase of mitotic cell cycle	85	12	8.22
	chromosome	124	14	8.22
	DNA metabolic process	257	19	7.88
	cell cycle checkpoint	48	9	7.40
	DNA replication	102	12	7.31
	spindle	39	8	6.95
	regulation of mitosis	41	8	6.77
	chromosomal part	96	11	6.62
	response to DNA demage stimulus	162	12	5.13
	mitotic spindle organization and biogenesis	10	4	4.96
	microtubule cytoskeleton organization and biogenesis	35	6	4.85
	spindle organization and biogenesis	11	4	4.77
	microtubule cytoskeleton	152	11	4.64
	DNA integrity checkpoint	24	5	4.55
	response to endogenous stimulus	200	12	4.21
	interphase	68	7	4.09
	intracellular non membrane bound organelle	632	23	4.02
	non membrane bound organelle	632	23	4.02

TABLE S5C	(continue 2/5) - Gene Ontology Analysis of correlation based target genes			
	GO term description	# Total annotated genes	# Correlation linked genes	PV $(-log_{10})$
**hsa-miR-133a**				
	structural constituent of muscle	33	11	16.00
	regulation of muscle contraction	19	8	12.67
	contractile fiber part	23	8	11.87
	sarcomere	14	7	11.79
	striated muscle contraction	14	7	11.79
	contractile fiber	25	8	11.53
	myofibril	19	7	10.63
	regulation of multicellular organism process	151	11	8.34
	system process	563	17	6./93
	cytoskeleton	368	12	5.26
	regulation of heart contraction	25	4	4.65
**hsa-miR-143**				
	collagen	23	6	7.13
	proteinaceous extracellular matrix	98	8	5.27
	extracellular matrix	100	8	5.20
	extracellular matrix part	57	6	4.67
**hsa-miR-145**				
	proteinaceous extracellular matrix	98	20	11.39
	extracellular matrix	100	20	11.22
	extracellular matrix part	57	13	8.20
	collagen	23	9	8.12
	extracellular region part	339	30	7.36
	extracellular region	448	35	7.16
**hsa-miR-152**				
	collagen	23	11	13.83
	extracellular matrix part	57	12	10.14
	proteinaceous extracellular matrix	98	12	7.32
	extracellular matrix	100	12	7.22
	extracellular region part	339	18	5.11
	extracellular region	448	20	4.52
**hsa-miR-17-5p**				
	mitotic cell cycle	153	8	6.37
	cell cycle phase	170	8	6.03
	cell cycle	315	10	5.89
	cell cycle process	193	8	5.61
	spindle	39	4	4.52
	mitosis	82	5	4.43
	M phase of mitotic cell cycle	85	5	4.36
**hsa-miR-193a**				
	collagen	23	5	4.54

TABLE S5C	(continue 3/5) - Gene Ontology Analysis of correlation based target genes			
	GO term description	# Total annotated genes	# Correlation linked genes	PV ($-log_{10}$)
**hsa-miR-199b**				
	collagen	23	11	10.22
	extracellular matrix part	57	15	9.34
	proteinaceous extracellular matrix	98	19	9.20
	extracellular matrix	100	19	9.05
	extracellular region part	339	34	7.85
	extracellular region	448	40	7.72
	organ development	572	38	4.24
**hsa-miR-206**				
	structural constituent of muscle	33	8	11.40
	striated muscle contraction	14	6	10.34
	regulation of muscle contraction	19	6	9.40
	contractile fiber part	23	6	8.83
	contractile fiber	25	6	8.59
	sarcomere	14	5	8.19
	myofibril	19	5	7.44
	regulation of multicellular organism process	151	9	7.15
	system process	563	15	7.05
	structural molecule activity	244	9	5.39
	actin cytoskeleton	129	7	5.35
	regulation of heart contraction	25	4	5.10
	muscle development	93	6	5.07
**hsa-miR-223**				
	response to external stimulus	313	12	5.99
	response to wounding	191	9	5.28
	plasma membrane	1429	23	4.52
	locomotory behaviour	97	6	4.31
**hsa-miR-30b**				
	extracellular matrix part	57	4	5.87
	extracellular matrix structural constituent	27	3	5.07
	proteinaceous extracellular matrix	98	4	4.92
	extracellular matrix	100	4	4.88
	basement membrane	37	3	4.65
**hsa-miR-30d**				
	collagen	23	10	11.31
	proteinaceous extracellular matrix	98	15	9.35
	extracellular matrix part	57	12	9.23
	extracellular matrix	100	15	9.22
	extracellular region part	339	24	7.57
	extracellular region	448	25	5.88
	organ development	572	26	4.45

TABLE S5C	(continue 4/5) - Gene Ontology Analysis of correlation based target genes			
	GO term description	# Total annotated genes	# Correlation linked genes	PV $(-log_{10})$
**hsa-miR-324-3p**				
	extracellular matrix part	57	11	6.51
	collagen	23	7	5.75
	proteinaceous extracellular matrix	98	13	5.63
	extracellular matrix	100	13	5.53
	extracellular region part	339	23	4.30
**hsa-miR-328**				
	collagen	23	6	5.54
	extracellular matrix part	57	8	5.05
**hsa-miR-374**				
	extracellular matrix structural constituent	27	3	4.78
**hsa-miR-378**				
	structural constituent of muscle	33	7	9.49
	myofibril	19	6	9.33
	regulation of muscle contraction	19	6	9.33
	contractile fiber part	23	6	8.77
	contractile fiber	25	6	8.53
	sarcomere	14	5	8.14
	striated muscle contraction	14	4	6.14
	regulation of multicellular organism process	151	7	4.84
	actin cytoskeleton	129	6	4.21
**hsa-miR-484**				
	M phase of mitotic cell cycle	85	10	10.45
	cell cycle process	193	13	10.44
	mitotic cell cycle	153	12	10.41
	cell cycle phase	170	12	9.87
	M phase	114	10	9.17
	mitosis	82	9	9.15
	cell cycle	315	14	8.84
	mitotic sister chromatide segregation	16	3	4.02
**hsa-miR-499**				
	structural constituent of muscle	33	10	14.33
	regulation of muscle contraction	19	8	12.87
	contractile fiber part	23	7	10.13
	contractile fiber	25	7	9.84
	sarcomere	14	6	9.78
	striated muscle contraction	14	6	9.78
	myofibril	19	6	8.84
	actin cytoskeleton	129	10	8.11
	regulation of multicellular organism process	151	10	7.45
	system process	563	16	6.52
	cytoskeleton	368	12	5.51
	structural molecule activity	244	10	5.51

TABLE S5C	(continue 5/5) - Gene Ontology Analysis of correlation based target genes			
	GO term description	# Total annotated genes	# Correlation linked genes	PV $(-log_{10})$
**hsa-miR-574**				
	extracellular matrix part	57	12	7.64
	collagen	23	8	7.11
	proteinaceous extracellular matrix	98	14	6.57
	extracellular matrix	100	14	6.46
	extracellular region part	339	23	4.48
	transmembrane receptor protein kinase activity	51	8	4.28
**hsa-miR-594**				
	collagen	23	5	4.82
	extracellular matrix part	57	7	4.82
**hsa-miR-663**				
	collagen	23	4	4.25
	extracellular matrix part	57	6	4.85
**hsa-miR-9**				
	cell cycle process	193	17	12.22
	cell cycle phase	170	16	11.95
	mitotic cell cycle	153	15	11.46
	mitosis	82	11	9.95
	M phase of mitotic cell cycle	85	11	9.78
	M phase	114	12	9.57
	cell cycle	315	17	8.83
	mitotic sister chromatide segregation	16	5	6.70
	sister chromatide segregation	17	5	6.55
	chromosome segregation	32	6	6.51
	chromosome	124	8	4.88
	chromosomal part	96	7	4.67
	chromosome condensation	10	3	4.11
**hsa-miR-9***				
	mitosis	82	8	9.14
	M phase of mitotic cell cycle	85	8	9.01
	M phase	114	8	7.99
	cell cycle process	193	9	7.41
	mitotic cell cycle	153	8	6.98
	cell cycle phase	170	8	6.63
	cell cycle	315	9	5.60
	microtubule cytoskeleton	152	6	4.60
	mitotic sister chromatide segregation	16	3	4.51
	sister chromatide segregation	17	3	4.43
**hsa-miR-93**				
	cell cycle phase	170	9	7.52
	cell cycle process	193	9	7.04
	M phase	114	7	6.34
	mitotic cell cycle	153	7	5.47
	cell cycle	315	9	5.26
	mitosis	82	5	4.61
	M phase of mitotic cell cycle	85	5	4.53

TABLE S5D	Gene Ontology analysis of target genes based on both correlation and miRNA seed occurrence			
	GO term description	# Total annotated genes	# Correlation linked genes	PV $(-log_{10})$
**hsa-miR-1**				
	sarcomere	14	4	8.45
	myofibril	19	4	7.86
	contractile fiber part	23	4	7.50
	contractile fiber	25	4	7.35
	actin cytoskeleton	129	4	4.45
**hsa-miR-133a**				
	sarcomere	14	3	5.68
	myofibril	19	3	5.24
	contractile fiber part	23	3	4.99
	contractile fiber	25	3	4.88
	actin cytoskeleton	129	4	4.06
**hsa-miR-145**				
	proteinaceous extracellular matrix	98	10	4.83
	extracellular matrix	100	10	4.75
**hsa-miR-152**				
	collagen	23	8	10.78
	extracellular matrix part	57	9	8.70
	proteinaceous extracellular matrix	98	9	6.59
	extracellular matrix	100	9	6.52
	extracellular region	448	14	4.27
	extracellular region part	339	12	4.23
**hsa-miR-17-5p**				
	mitotic cell cycle	153	6	5.44
	cell cycle phase	170	6	5.17
	cell cycle process	193	6	4.86
	mitosis	82	4	4.09
	M phase of mitotic cell cycle	85	4	4.03
**hsa-miR-193a**				
	collagen	23	5	5.67
**hsa-miR-199b**				
	collagen	23	8	7.94
	proteinaceous extracellular matrix	98	14	7.91
	extracellular matrix	100	14	7.79
	extracellular matrix part	57	11	7.72
	extracellular region part	339	20	4.62
**hsa-miR-206**				
	sarcomere	14	3	6.08
	myofibril	19	3	5.66
	contractile fiber part	23	3	5.40
	contractile fiber	25	3	5.28
	intracellular non membrane bound organelle	632	7	5.09
	non membrane bound organelle	632	7	5.09
	skeletal muscle development	31	3	4.99
	striated muscle development	40	3	4.66
	actin cytoskeleton	129	4	4.60
	cytoskeleton protein binding	159	4	4.24

TABLE S5D	(continue 2/2) - Gene Ontology analysis of target genes based on both correlation and miRNA seed occurrence			
	GO term description	# Total annotated genes	# Correlation linked genes	PV $(-log_{10})$
**hsa-miR-30d**				
	collagen	23	8	9.30
	extracellular matrix part	57	9	7.08
	proteinaceous extracellular matrix	98	11	6.96
	extracellular matrix	100	11	6.87
	extracellular region part	339	19	6.66
	extracellular region	448	20	5.43
**hsa-miR-374**				
	extracellular matrix structural constituent	27	3	5.59
**hsa-miR-378**				
	structural constituent of muscle	33	5	7.74
	myofibril	19	4	6.85
	contractile fiber part	23	4	6.50
	contractile fiber	25	4	6.35
	sarcomere	14	3	5.24
	striated muscle contraction	14	3	5.24
	regulation of muscle contraction	19	3	4.82
**hsa-miR-484**				
	cell cycle process	193	9	7.96
	mitotic cell cycle	153	8	7.46
	cell cycle	315	10	7.25
	cell cycle phase	170	8	7.11
	mitosis	82	6	6.51
	M phase of mitotic cell cycle	85	6	6.42
	M phase	114	6	5.67
**hsa-miR-499**				
	sarcomere	14	3	5.59
	striated muscle contraction	14	3	5.59
	myofibril	19	3	5.17
	contractile fiber part	23	3	4.91
	contractile fiber	25	3	4.80
**hsa-miR-9**				
	cell cycle process	193	7	4.65
**hsa-miR-93**				
	cell cycle phase	170	7	6.20
	cell cycle process	193	7	5.83
	mitotic cell cycle	153	6	5.22
	M phase	114	5	4.63
	cell cycle	315	7	4.43
