# Supplementary material for: Antagonism Pattern Detection between MicroRNA and Target Expression in Ewing’s Sarcoma
Source: PLoS One. 2012 Jul 25;7(7):e41770. doi: 10.1371/journal.pone.0041770 (PMC3404966; doi:10.1371/journal.pone.0041770)
Supplement: Table S7 — List of miRNA hubs in common between the antagonism-based network and the correlation-based network. (PDF) [file pone.0041770.s009.pdf]

**TABLE S7**	**List of common miRNA hubs between antagonism-based network and correlation-based network**	
	**Antagonism connectivity**	**Correlation connectivity**
hsa-miR-324-3p	160	271
hsa-miR-328	127	208
hsa-miR-146b	121	82
hsa-miR-125a	116	145
hsa-miR-574	108	73
hsa-miR-342	104	71
hsa-miR-594	93	169
hsa-miR-221	80	102
hsa-miR-486	75	69
hsa-miR-143	73	54
hsa-miR-92	72	126
hsa-miR-145	69	95
hsa-miR-423	66	76
hsa-miR-30d	65	236
hsa-miR-130b	62	70
hsa-miR-191	60	88
hsa-miR-222	59	101
hsa-miR-30b	55	55
hsa-miR-148b	55	78
hsa-miR-193a	54	95
hsa-miR-210	51	130
